# Identification of ARUK2002821 as an isoform-selective PI5P4Kα inhibitor†

**DOI:** 10.1039/d3md00039g

**Published:** 2023-04-17

**Authors:** Henriëtte M. G. Willems, Simon Edwards, Helen K. Boffey, Stephen J. Chawner, Christopher Green, Tamara Romero, David Winpenny, John Skidmore, Jonathan H. Clarke, Stephen P. Andrews

**Affiliations:** a The ALBORADA Drug Discovery Institute, University of Cambridge Island Research Building, Cambridge Biomedical Campus, Hills Road Cambridge CB2 0AH UK spa26@cam.ac.uk

## Abstract

The phosphatidylinositol 5-phosphate 4-kinases (PI5P4Ks) play a central role in regulating cell signalling pathways and, as such, have become therapeutic targets for diseases such as cancer, neurodegeneration and immunological disorders. Many of the PI5P4Kα inhibitors that have been reported to date have suffered from poor selectivity and/or potency and the availability of better tool molecules would facilitate biological exploration. Herein we report a novel PI5P4Kα inhibitor chemotype that was identified through virtual screening. The series was optimised to deliver ARUK2002821 (36), a potent PI5P4Kα inhibitor (pIC_50_ = 8.0) which is selective *vs.* other PI5P4K isoforms and has broad selectivity against lipid and protein kinases. ADMET and target engagement data are provided for this tool molecule and others in the series, as well as an X-ray structure of 36 solved in complex with its PI5P4Kα target.

## Abstract 2

The PI5P4Ks play a central role in regulating cell signalling pathways. ARUK2002821 is reported as a potent and selective PI5P4Kα inhibitor (pIC_50_ = 8.0). ADMET and target engagement data are provided, with a ligand-protein X-ray structure, optimised to deliver ARUK2002821.

## Graphical abstract

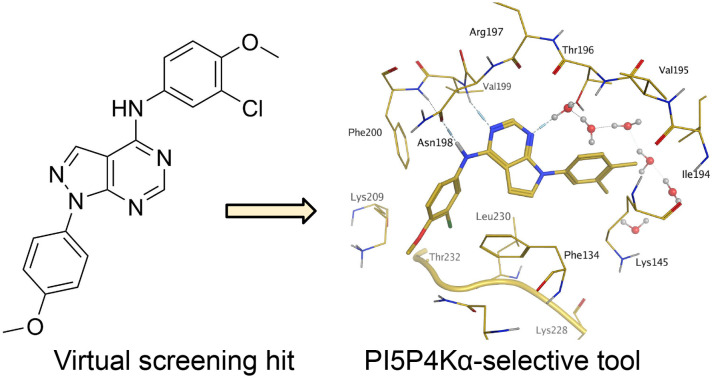

## Introduction

Phosphatidylinositol and the phosphorylated derivatives of this amphiphilic membrane phospholipid have integral roles in many cellular processes that are mediated through cell signalling and control of membrane dynamics.^1^ Phosphoinositide species are defined by the accumulation of phosphate groups onto the cyclic *myo*-inositol ring that constitutes the accessible hydrophilic headgroup when the lipid is integrated into a cellular membrane. The seven discovered phosphoinositide species (phosphorylated on positions 3–5 of the 6-carbon ring of this phosphatidylinositol headgroup) have been suggested to have mediator roles in a wide variety of cellular metabolic processes such as channel regulation, membrane trafficking, proliferation and cell death/stress responses such as autophagy and apoptosis.^1–4^ As a consequence, the roles of phosphoinositides in human diseases have received much recent attention and are well documented in developmental disorders (including channelopathies and ciliopathy syndromes), neurodegeneration and cancer.^5–9^

Mammals express three isoforms (α, β and γ) of phosphatidylinositol 5-phosphate 4-kinases (PI5P4Ks), the enzymes that generate phosphatidylinositol bisphosphate (PIP_2_) from phosphatidylinositol phosphate (PIP), principally *via* the conversion of PI(5)*P* to PI(4,5)*P*_2_. Variation in catalytic site and putative G-loop sequences may account for the large differences in intrinsic *in vitro* kinase activity between the isoforms (PI5P4Kα being the most active isoform).^10^ The PI5P4Ks have established links to cancer,^9,11^ specific associations suggest that inhibition of PI5P4Kα may be beneficial in p53 mutant breast cancer^12,13^ and acute myeloid leukaemia^14^ where *PIP4K2A* has been identified as a cytogenetic risk factor.^15,16^ This association potentially reflects the role of PI5P4Kα in cell proliferation and the higher levels of expression in hematopoietic cells.^17^

**Fig. 1:**
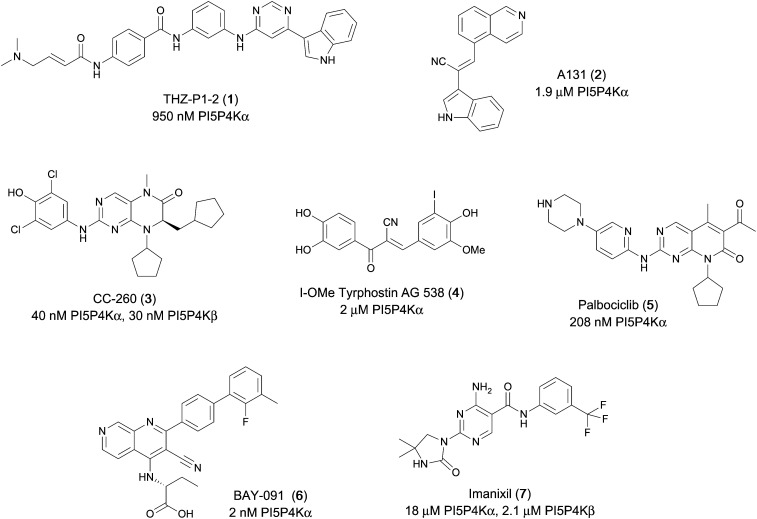
Published PI5P4Kα inhibitors with their reported activity values.

Various PI5P4K inhibitors have been reported to have a therapeutic effect in oncology settings.^18,19^ Covalent pan-PI5P4K inhibitor THZ-P1-2 (1) was shown to have anti-proliferative activity in acute myeloid and lymphoblastic leukaemias (AML/ALL)^19–22^ and the small molecule A131 (2), which was shown to target mitotic pathways and PI5P4Ks, was effective in cancer cell-specific lethality^18^ (Fig. 1). Compound CC260 (3), with dual activity at PI5P4Kα and PI5P4Kβ,^13^ reduced proliferation in p53 null cancer cell lines in the presence of stress caused by nutrient depletion, in concordance with earlier findings.^12^ More specifically, selective inhibition of PI5P4Kα may also have positive therapeutic effects, as demonstrated by the genetic depletion of PI5P4Kα in p53 null THP-1 cells which resulted in the inhibition of proliferation and also prevented AML development in xenografts.^14^

There have been other reports of small molecule PI5P4Kα inhibitors, such as the tyrphostins, with I-OMe-tyrphostin AG538 (4) having the highest reported activity.^23^ The CDK4/6 inhibitor, palbociclib (5) has also been reported to be a PI5P4Kα inhibitor,^24^ however, this compound was found to be inactive in ADP-Glo assays for all 3 isoforms of PI5P4K in our laboratory.^25^ BAY-091 (6) was recently identified as a selective PI5P4Kα chemical probe following HTS of 3.9 million compounds at Bayer^26^ but the compound did not show anti-proliferative activity in p53-null cells. Imanixil (7) was developed by Sanofi-Aventis who subsequently characterised it as a PI5P4Kβ inhibitor with some PI5P4Kα activity.^27^

## Results

As part of our investigations we were interested in identifying inhibitors for each of the PI5P4K isoforms, α, β and γ. We have previously described efforts to improve the PI5P4Kγ inhibitor, NIH12848,^28^ and a virtual screening approach to identify novel PI5P4Kγ inhibitors.^25^ Owing to the high sequence similarity between the PI5P4K isoforms, a single virtual screening approach was adopted for PI5P4Kα and PI5P4Kγ.^25^ To identify biologically-active PI5P4Kα inhibitors, the 960 virtual screening hits previously described were screened in an ADP-Glo kinase functional assay using 4 as a positive control. This identified 9 compounds with pIC_50_s > 5 against PI5P4Kα, which are shown in Table 1. From this set, 8 had the highest activity (pIC_50_ = 6.4) and had an active near neighbour (9).

**Table 1:** PI5P4Kα and PI5P4Kγ+ pIC_50_s of the top virtual screening hits

		Inhibition of PI5P4K (ADP-Glo)			Physicochemical properties	
		PI5P4Kα pIC_50_	PI5P4Kα LE	PI5P4Kγ+ pIC_50_	MW	*X* log *P*
8	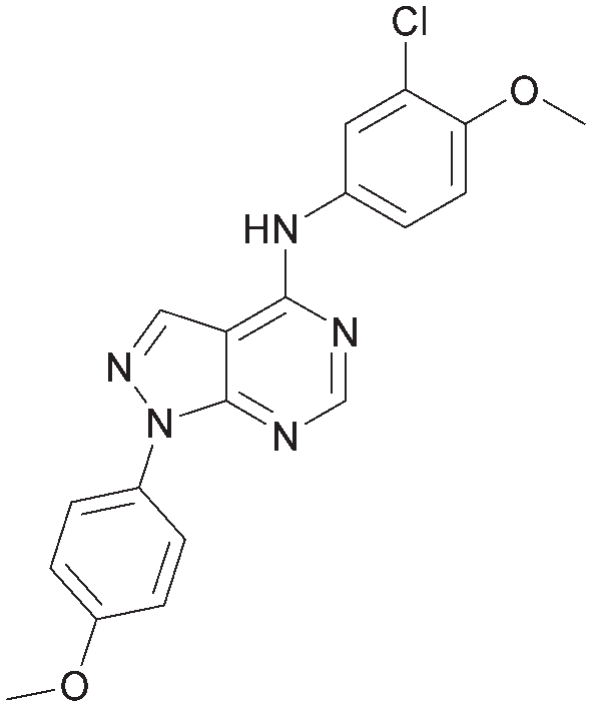	6.4	0.33	4.9	382	4.2
9	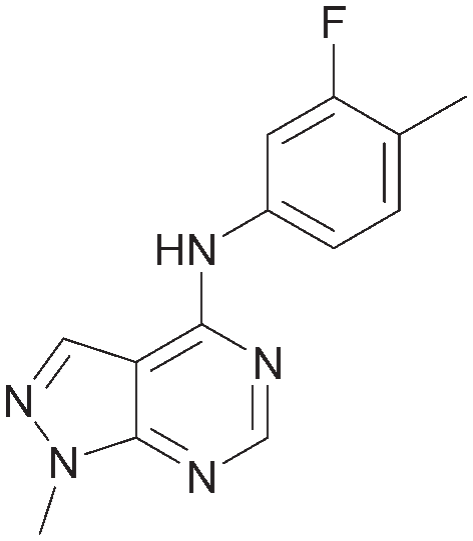	5.2	0.38	<4.3	257	2.4
10	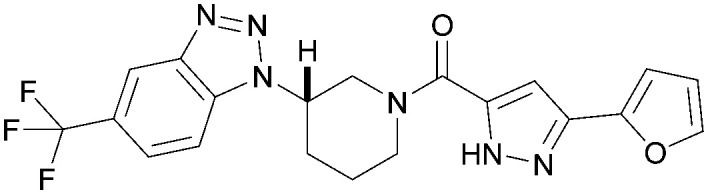	5.7	0.26	ND	430	4.6
11	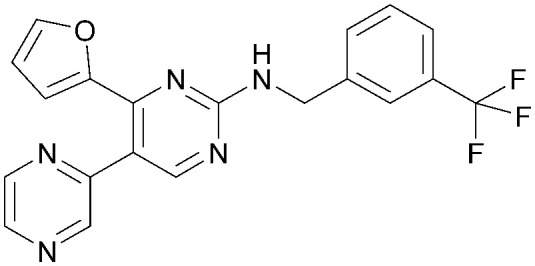	5.4	0.26	4.9	397	3.4
12	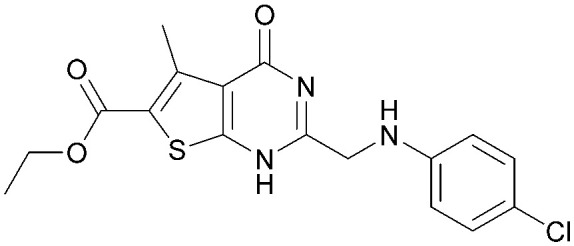	5.1	0.28	<4.3	378	3.8
13	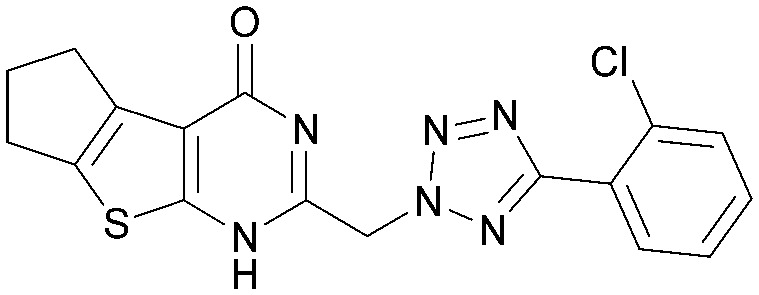	5.6	0.30	4.8	385	3.5
14	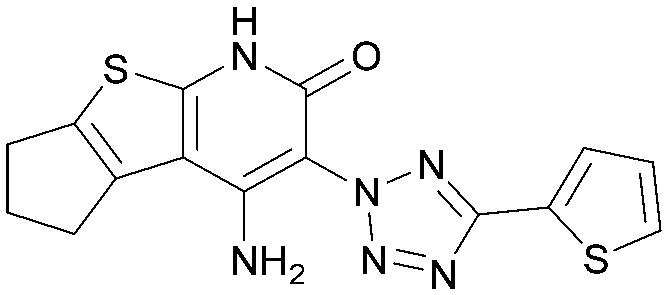	5.4	0.32	4.7	356	2.5
15	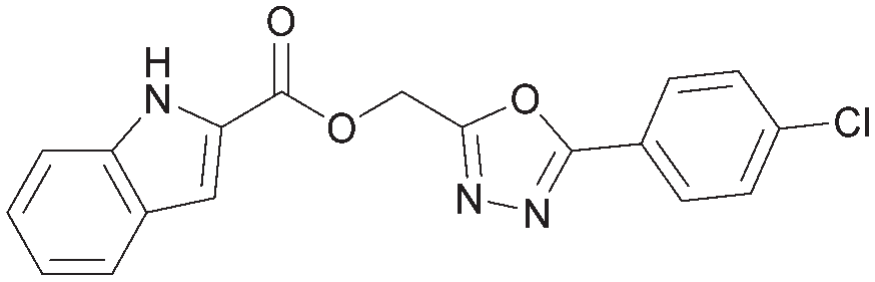	5.3	0.30	<4.3	354	5.5
16	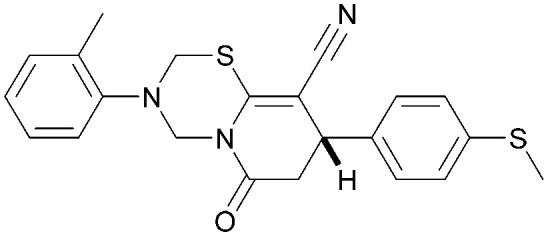	5.5	0.27	<4.3	408	5.4

These hits were followed up with analogue purchases where possible, with compounds being selected by substructure, Tanimoto similarity or shape similarity. In total, 60 analogues were purchased for 10, but only two were weakly active (Table S1†), and 42 analogues of 11 were purchased, 2 of which showed pIC_50_s > 5, but none were more potent than 11 (Table S2†). None of the 24 analogues purchased for the 12/13/14 cluster showed activity and no suitable commercial analogues of 15 or 16 were identified. In contrast, several purchased analogues of the 8 series showed PI5P4Kα activity, with two showing increased potency. This series was therefore selected for further optimisation. PI5P4Kα and PI5P4Kγ+ data for all purchased analogues of 8 is available in ESI† (Table S3).

To further probe the utility of compound 7 as a hit for development, the compound was synthesised then screened in-house using previously described ADP-Glo assays for PI5P4Kα, β and γ+.^28^ The compound was found to be inactive in all cases in our hands. The chemical structure was verified by NMR, LCMS and small molecule X-ray crystallographic analysis and found to be consistent with the reported structure.^27^ Further, compound 7 was screened externally against two commercially-available lipid kinase assay panels and was found to have essentially no activity (see ESI† Tables S4–S9 for further details on the screening and small molecule X-ray crystal structure). This chemotype was not pursued for further development.

Analogues of 8 were purchased or synthesised then screened against PI5P4Kα in iterations in order to understand the SAR of the series, with PI5P4Kγ+ being used as a control for isoform selectivity (Table 2). In general, this chemotype showed little or no measurable inhibition of PI5P4Kγ+ activity. Lipophilic substituents on both aryl rings, R^1^ and R^2^, were generally preferred for PI5P4Kα activity and aryl group R^2^ showed a preference for a lipophilic substituent at position 4 of the ring, such as methoxy (8) or methyl (17) *vs.* unsubstituted (18). An electron-withdrawing chloro group at the 4-position did not work well (19) unless combined with a substituent at the 2-position (20) which is postulated to induce a conformational twist, as discussed in further detail below. Similarly, chloro was not tolerated at the 3-position (21) unless there was an *ortho* substituent to elicit the conformational twist (compare 22 with a para group in 23). Conversely, an electron-donating methyl group was tolerated at position 3 or 4 without a conformational lock (24 and 17). These subtle differences in chloro *vs.* methyl are discussed in detail below. The combination of 4-methyl with a second substituent worked well in the cases of 25 and 26. There were early indications that an aromatic heterocycle may be tolerated at position R^2^ in some cases (compare 27 and 28) but replacing this aryl group with a small aliphatic group ablated activity (29). A small lipophilic group was not tolerated at position R^1^ (30) and deletion of the 3-Cl or 4-OMe from the R^1^ aryl ring caused a significant drop in activity (compare 31 with 25 and 32 with 26). It was desirable to introduce a more polar substituent on the R^1^ aryl ring or heteroatoms within the ring to modulate physicochemical properties and early attempts to do so suggested this might be possible with the correct combination and orientation of substituents (compare 33, 34 and 35).

**Table 2:** SAR exploration at positions R^1^ and R^2^

	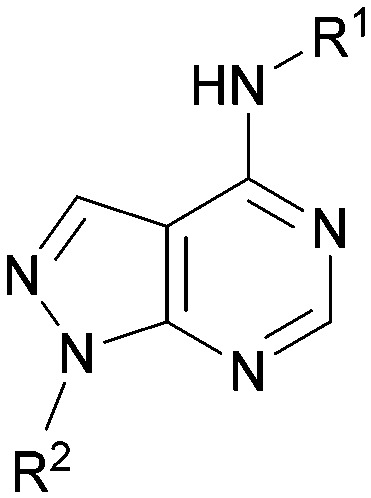		Inhibition of PI5P4K (ADP-Glo)			Physicochemical properties	
	R^1^	R^2^	PI5P4Kα pIC_50_	PI5P4Kα LE	PI5P4Kγ+ pIC_50_	MW	*X* log *P*
8	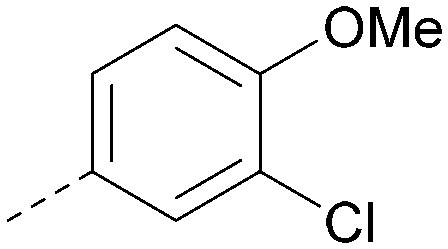	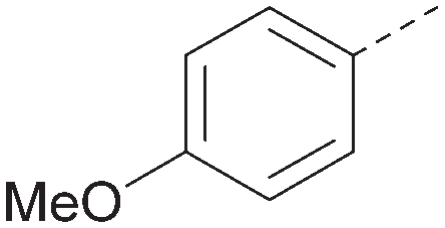	6.4	0.33	4.9	382	4.2
17	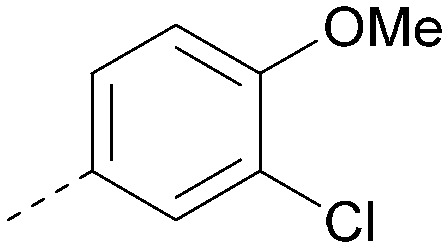	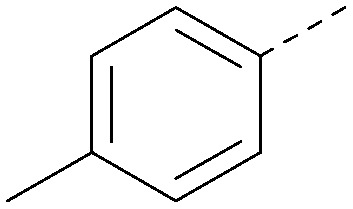	6.9	0.37	<4.3	366	4.5
18	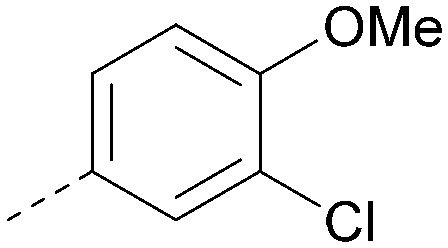	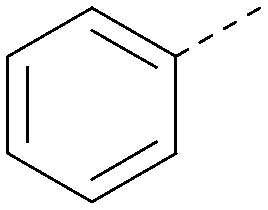	5.9	0.33	<4.3	352	4.2
19	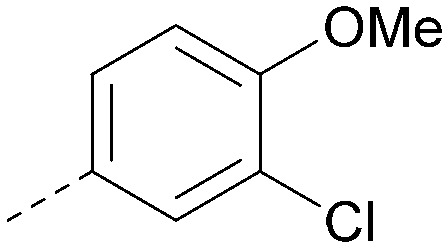	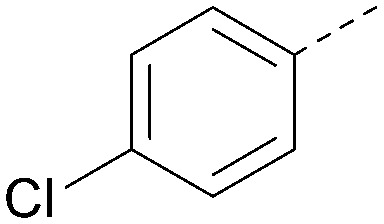	<4.3	NA	<4.9	386	4.8
20	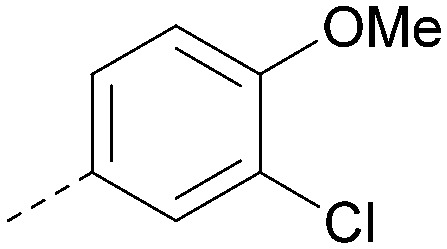	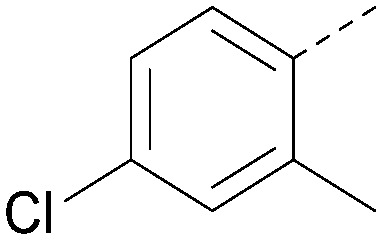	6.6	0.34	<4.6	400	5.1
21	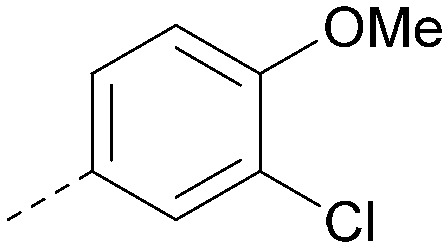	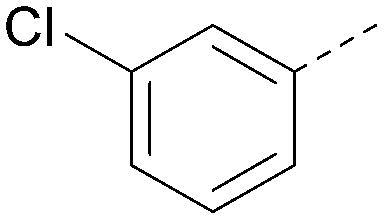	<4.7	NA	<4.3	386	4.8
22	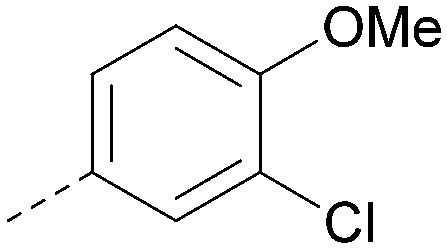	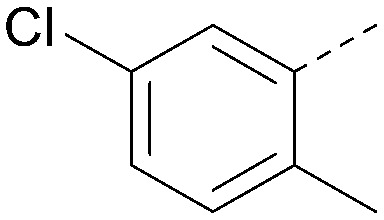	5.6	0.29	<4.5	400	5.1
23	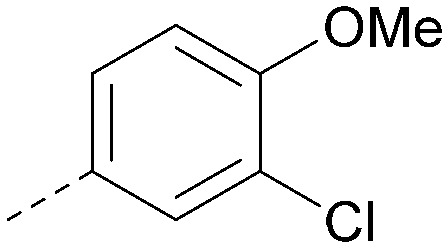	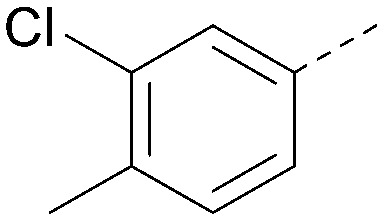	<4.3	NA	<4.3	400	5.1
24	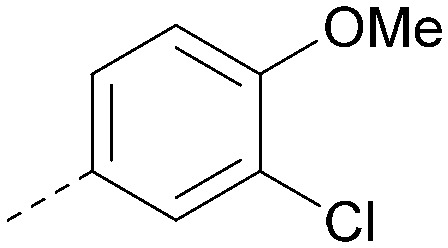	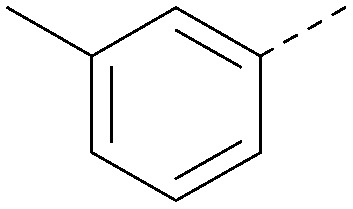	6.3	0.34	<4.3	366	4.5
25	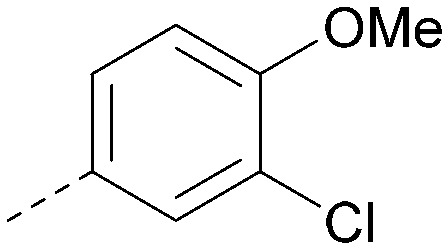	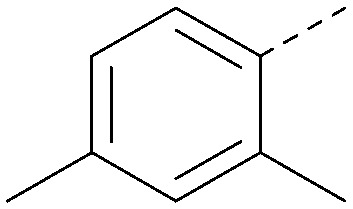	7.0	0.36	<4.3	380	4.8
26	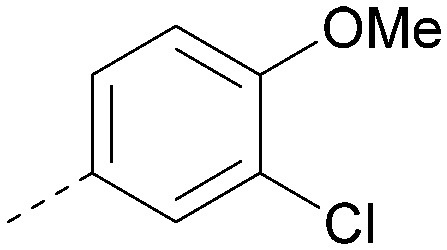	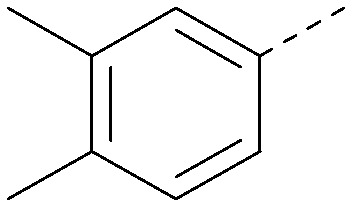	7.1	0.37	<4.3	380	4.8
27	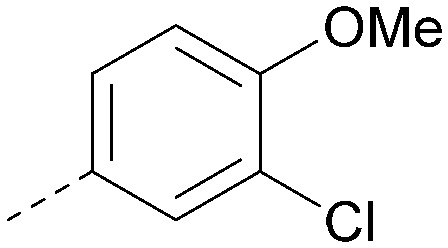	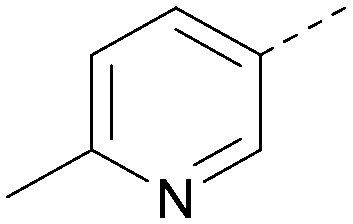	5.7	0.31	<4.3	367	3.1
28	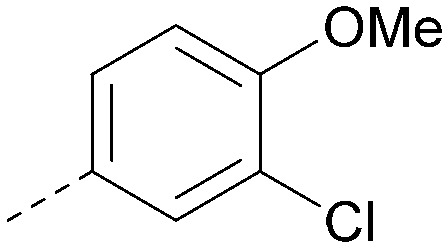	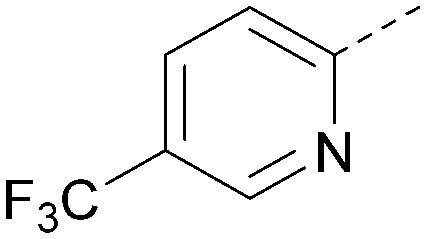	<4.3	NA	<4.3	421	4.3
29	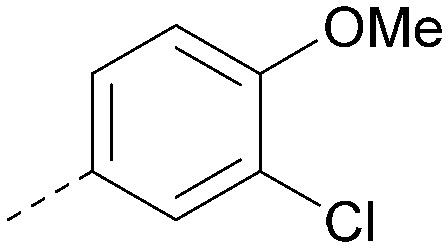	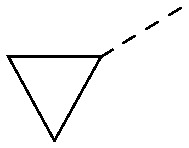	<4.6	NA	<4.7	316	3.2
30	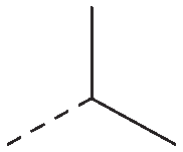	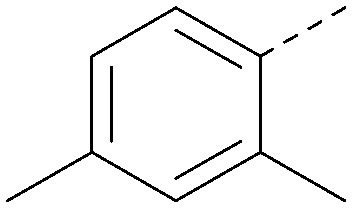	4.8	0.25	<4.2	281	3.4
31	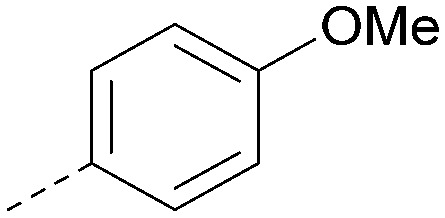	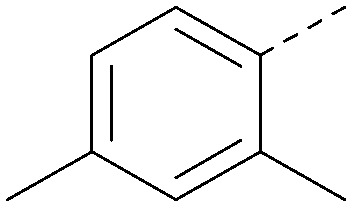	5.9	0.32	<4.3	345	4.2
32	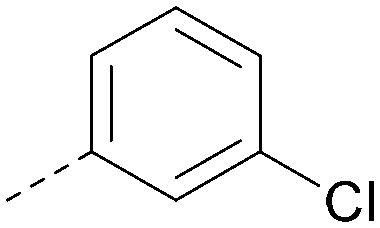	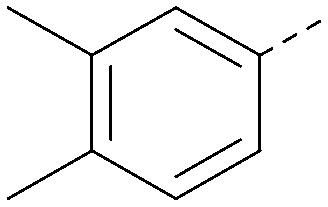	<4.3	0.24	<4.3	350	4.8
33	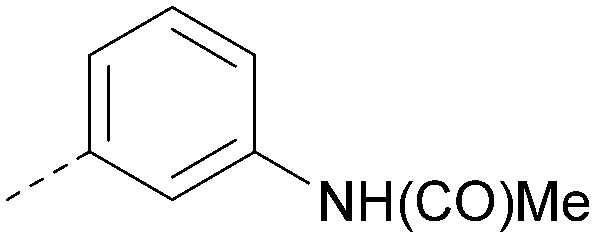	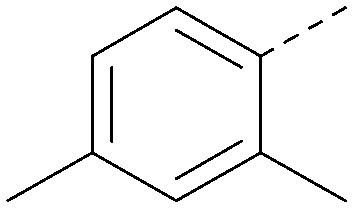	5.9	0.30	<4.7	372	3.9
34	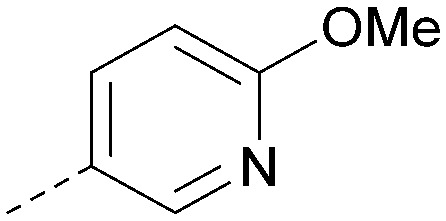	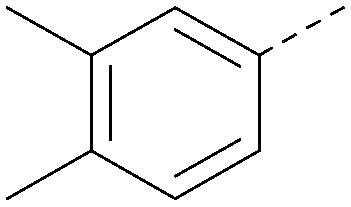	6.5	0.35	<4.3	346	3.3
35	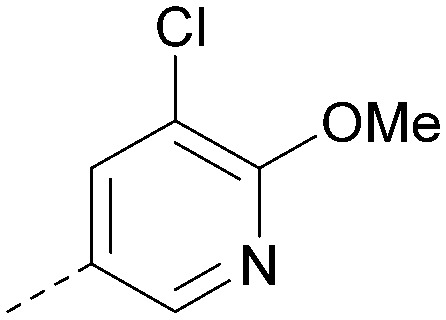	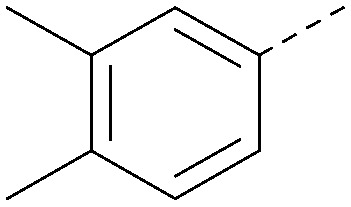	<4.3	0.22	<4.3	381	3.9

A number of analogues with variations to the bicyclic core was synthesised whilst keeping the R^1^ and R^2^ substituents fixed to provide matched pairs with 26 (Table 3). Replacement of the ring N by CH at position 8, to generate a pyrrolopyrimidine, proved the most successful strategy for increasing binding affinity and cell potency (36) whereas replacement of the ring N by CH at positions 1 or 3 led to complete loss of activity (37 and 38). The pyrrolopyrimidinone derivative, 39, showed a marked reduction in *X* log *P* compared to 36 and was equipotent to compound 26. Methylation at the 7-position was not tolerated (40).

**Table 3:** Variation of the core

	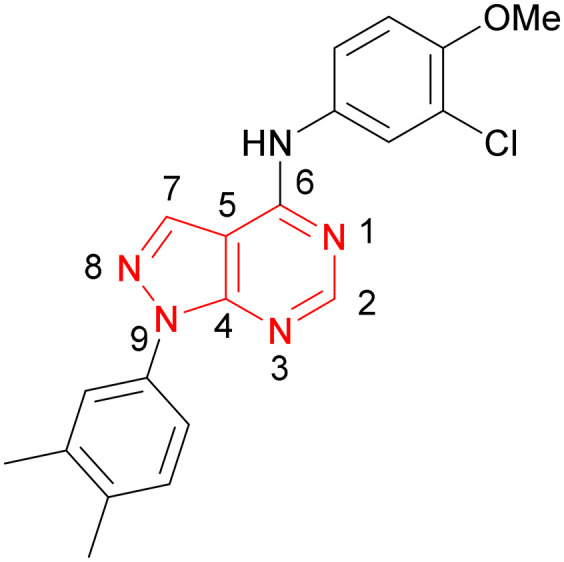	Inhibition of PI5P4K (ADP-Glo)			Physicochemical properties	
		PI5P4Kα pIC_50_	PI5P4Kα LE	PI5P4Kγ+ pIC_50_	MW	*X* log *P*
26	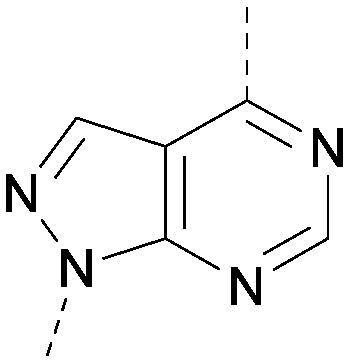	7.1	0.37	<4.7	380	4.8
36	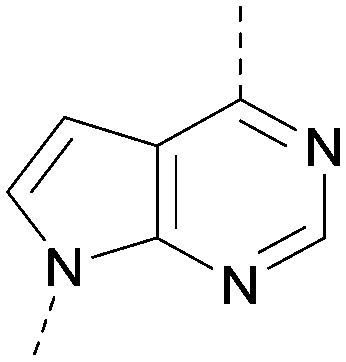	8.0	0.42	<4.3	379	5.8
37	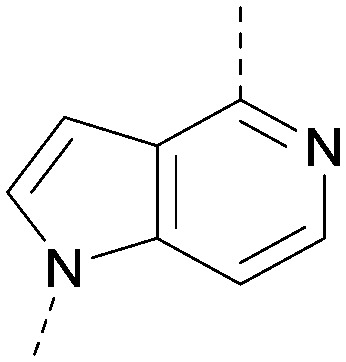	<4.6	NA	<4.4	378	6.3
38	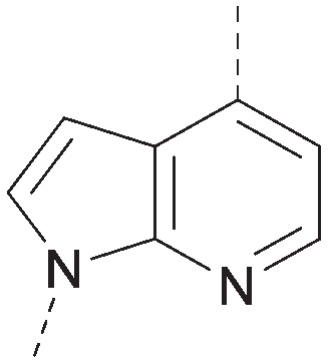	<4.3	NA	<4.5	378	6.0
39	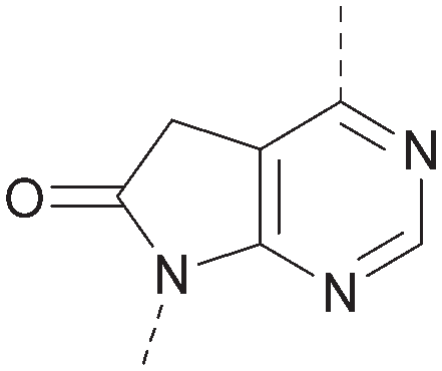	6.9	0.35	<4.6	395	4.9
40	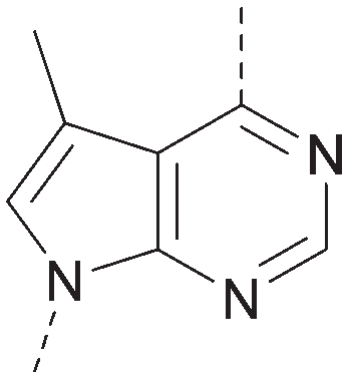	<4.3	NA	<4.3	393	6.1

Using the pyrrolopyrimidine template of 36 as a new lead, further analogues were synthesised. As with 25*vs.*26, 2,4-dimethylphenyl on the pyrrolopyrimidine core was slightly less active than 3,4-dimethyl (compare 36 and 41) and the 4-OMe matched pair with 8 also tracks well (42; Table 4). A primary aim was to improve molecular properties, particularly reducing lipophilicity. To this end, polar substituents and heteroaromatic rings were trialled in different positions for R^1^ and R^2^. Acetamide at the meta position (43) showed a marked drop in *X* log *P* compared to 36 but with a drop in activity and the para acetamide 44 showed a further drop in activity. Pyridyl derivative 45 showed a good balance of activity and *X* log *P*, whereas isoxazole 46, which had a favourable *X* log *P*, lost considerable potency. At position R^2^, a more polar aniline substituent was somewhat tolerated (47) whereas a small aliphatic group was again not tolerated (compare 48 and 29), nor were 5-membered heterocycles in different configurations (49, 50).

**Table 4:** SAR for varying R^1^ and R^2^ on the pyrrolopyrimidine core

	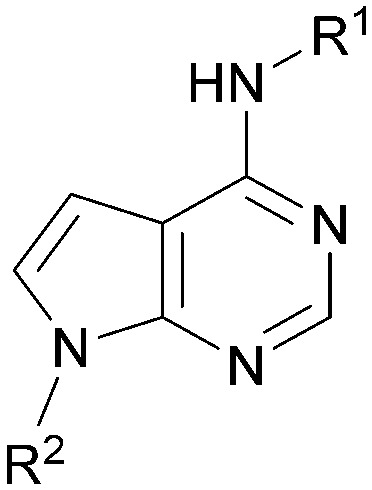		Inhibition of PI5P4K (ADP-Glo)			Physicochemical properties	
	R^1^	R^2^	PI5P4Kα pIC_50_	PI5P4Kα LE	PI5P4Kγ+ pIC_50_	MW	*X* log *P*
36	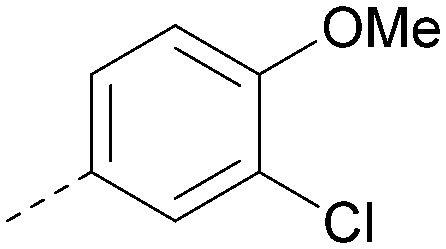	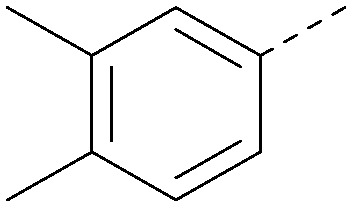	8.0	0.42	4.3	379	5.8
41	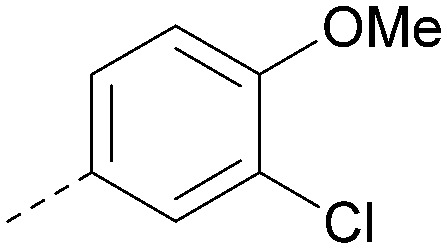	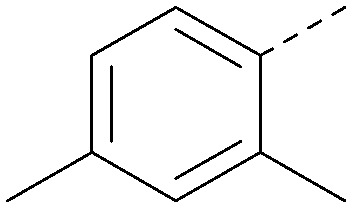	7.7	0.40	<4.5	379	5.8
42	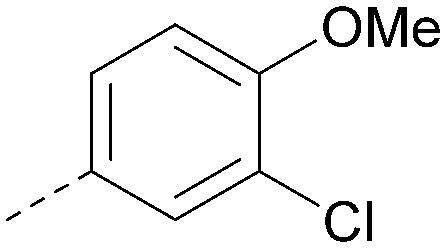	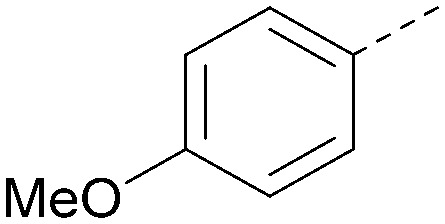	7.4	0.39	<4.5	381	5.2
43	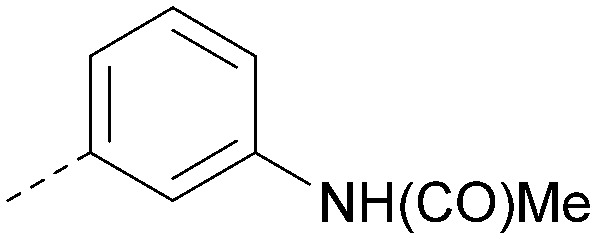	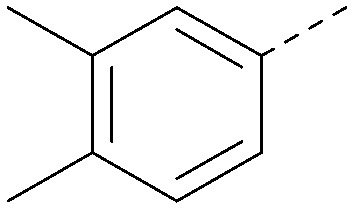	6.8	0.34	4.5	371	4.9
44	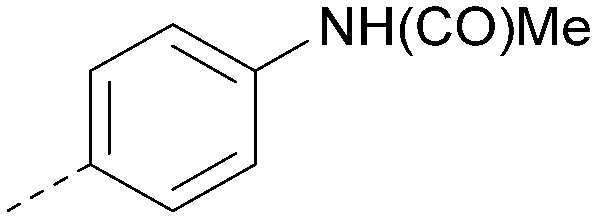	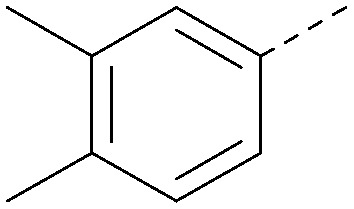	6.0	0.30	4.5	371	4.9
45	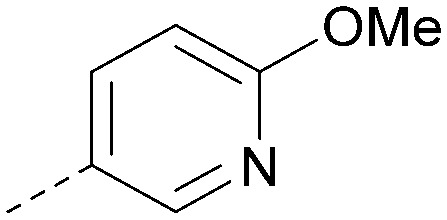	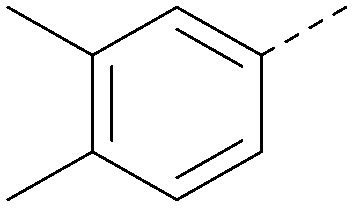	7.2	0.39	4.4	345	4.3
46	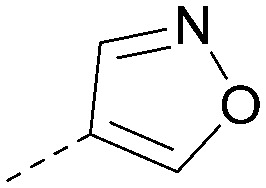	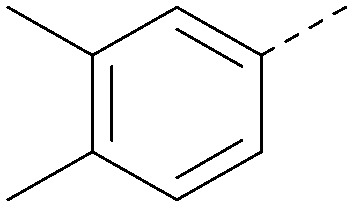	5.5	0.34	<4.5	305	3.3
47	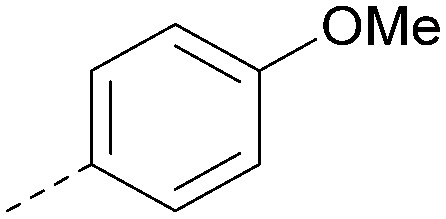	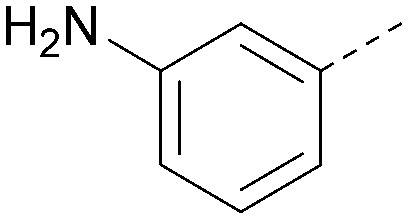	6.2	0.35	4.8	331	3.9
48	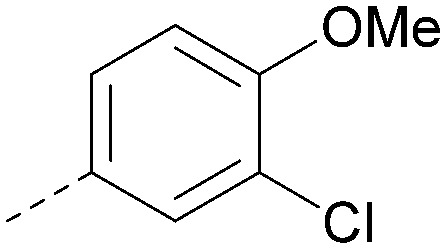	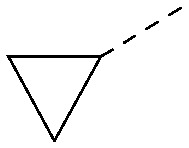	5.0	0.32	<4.3	315	4.2
49	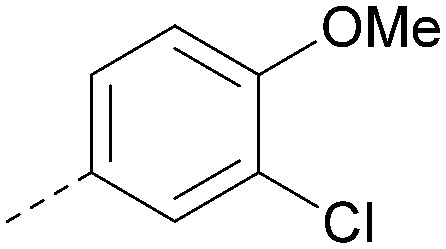	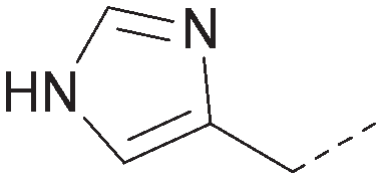	5.6	0.31	<4.8	355	3.9
50	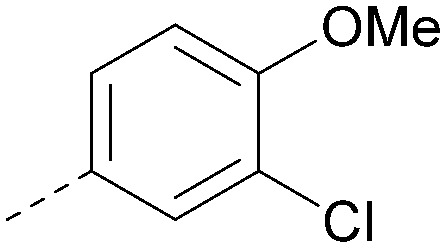	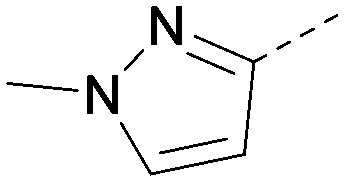	<5.0	NA	<4.5	355	4.3

A set of compounds from this series spanning different cores and R^1^ and R^2^ groups that were beneficial for potency and/or favourably modified physicochemical properties was profiled in further assays including PI5P4K isoform selectivity, a PI5P4Kα cellular assay to demonstrate target engagement and simple ADMET assays including permeability, solubility and microsomal stability (Table 5). In general, compounds from this series did not inhibit PI5P4Kγ+ or PI5P4Kβ and were able to engage PI5P4Kα in cells as measured using an InCELL Pulse thermostabilisation assay (DiscoverX). These compounds displayed moderate permeability scores and showed no efflux flags in MDCK-MDR1 cells, although solubility was generally low. Stability in mouse liver microsomes was moderate to low (Table 5).

**Table 5:** Target engagement and *in vitro* ADME data for selected compounds^c^

	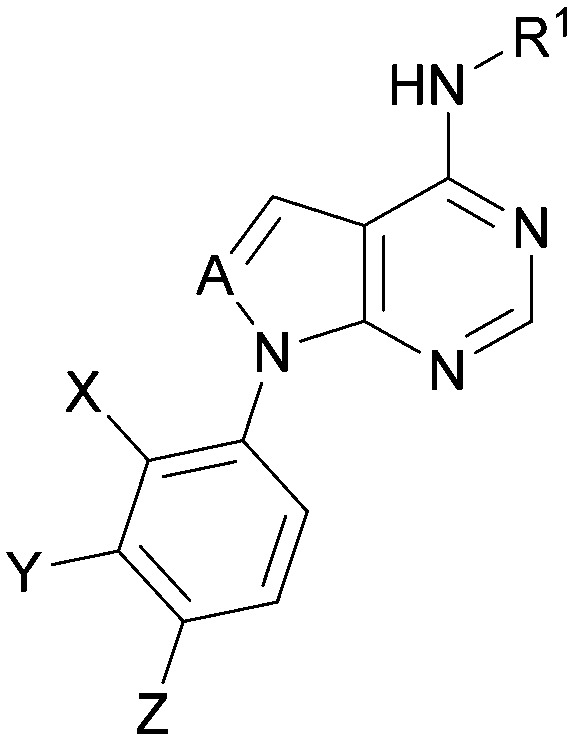					Inhibition of PI5P4K (ADP-Glo)			Cellular target engagement (InCELL pulse)	*In vitro* ADMET^a^			
	R^1^	X	Y	Z	A	PI5P4Kγ+ pIC_50_	PI5P4Kβ pIC_50_	PI5P4Kα pIC_50_	PI5P4Kα pIC_50_	*P*_app_ (A2B, 10^−6^ cm s^−1^)	ER	Solubility (μM)	MLM *t*_1/2_ (min)
20	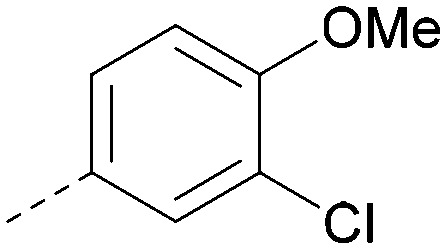	Me	H	Cl	N	<4.6	<4.6	6.6	6.4	6.4	0.7	10	26
ARUK2002821(36)	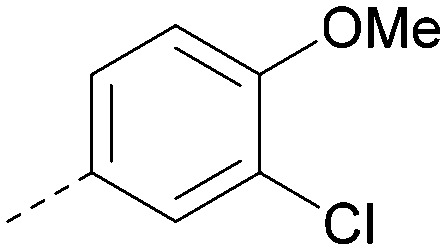	H	Me	Me	CH	<4.3	ND^b^	8.0	6.6	0.2	0.8	3	11
45	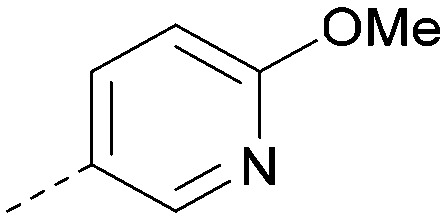	H	Me	Me	CH	<4.4	<4.6	7.2	<4.6	12	0.6	3	11
43	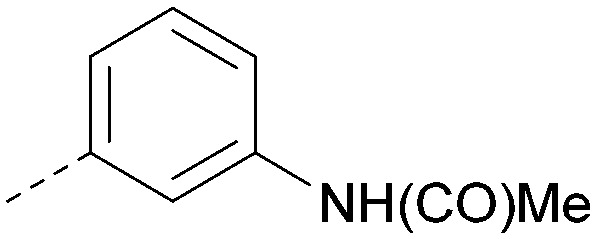	H	Me	Me	CH	<4.5	<4.6	6.8	5.7	14	0.9	3	24
39	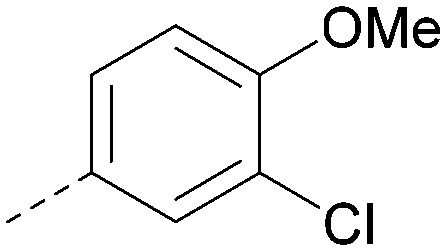	H	Me	Me	C(OH)	<4.6	ND	6.9	ND	17	0.7	1	16

a Experiments were performed at Cyprotex (see ESI†). *P*_app_ is apparent permeability and ER is efflux ratio, these were measured in MDCK-MDR1 cells. Solubility was determined at pH 7.4 in aqueous buffer. MLM *t*_1/2_ is the half-life of compound when incubated with mouse liver microsomes.

b 36 showed 4% inhibition when screened in this assay at a single concentration of 100 μM.

c ND = not determined.

Compound 36 (ARUK2002821) was identified as a promising lead in this chemical series, with good potency, selectivity *vs.* PI5P4Kβ and PI5P4Kγ+ and active cellular target engagement. To assess wider kinase selectivity, 36 was profiled against 140 protein kinases and 15 lipid kinases. When 36 was tested at 1 μM in the lipid kinase screen the only target with <50% residual activity was PI5P4Kα (15.6%) and the considerable drop to the next most inhibited target indicated high specificity (SPHK2; 81.7% residual activity; Table S10†). In the protein kinase screen no targets were inhibited by >50% at 1 μM; the lowest residual activities observed were for ERK1 and JAK3 (62 and 63%, respectively; Table S11†). Full data are provided in the ESI.†

**Fig. 2:**
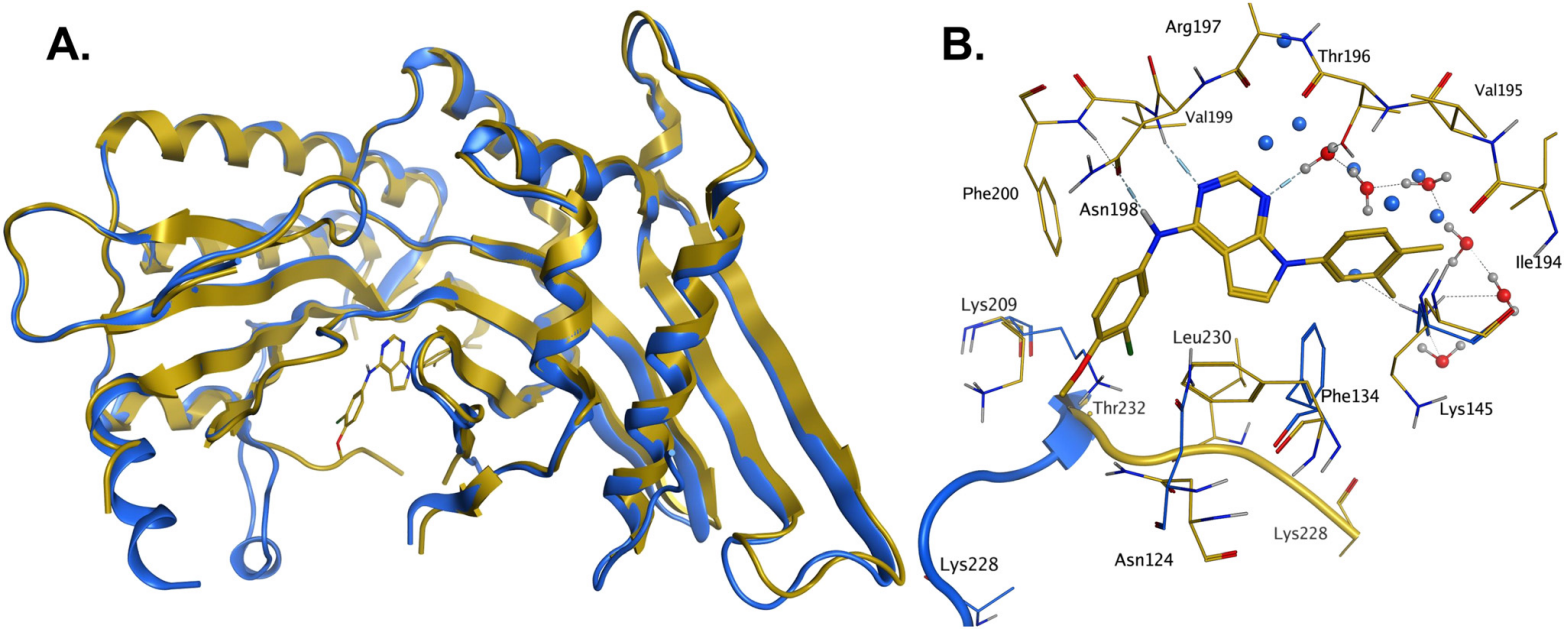
A: Crystal structure comparison of apo PI5P4Kα (pdb 2YBX; blue) and PI5P4Kα in complex with compound 36 (pdb 8C8C; gold). B: View of the active site of PI5P4Kα in complex with compound 36 (gold carbons). Residues of the apo structure 2YBX that are in a different orientation in the ligand-bound structure are shown in blue, and the Lys228–Thr232 loop segment of the apo structure as a blue ribbon. 2YBX waters are shown as blue spheres, and 8C8C waters with red oxygen atoms.

In order to solve a structure of a co-complex of PI5P4Kα with a ligand from this series, a truncated version of human PI5P4Kα was cloned into a bacterial expression vector. Using this approach, a PI5P4Kα protein construct comprising residues Asp35 to Thr406, with the region between residues 308–314 deleted, was successfully co-crystallised with 36 at a 2.1 Å resolution (Fig. 2, pdb 8C8C).

There is one PI5P4Kα chain in the asymmetric unit. This forms a dimer interface with a symmetry-related chain, which is similar to that seen in the published unliganded structure of PI5P4Kα (pdb 2YBX). Overall, the protein structure with 36 bound shows a high degree of similarity with that of the apo structure (Fig. 2A). However, the Lys228–Thr232 loop segment has a different orientation in the 36 complex, resulting in Leu230 becoming part of the binding pocket. In addition to the Lys228–Thr232 loop, four residues in the binding pocket are significantly re-oriented compared with apo structure 2YBX: Asn124, Phe134, Lys145 and Lys209 (Fig. 2B). The re-orientation of these residues upon ligand binding is also seen in several other crystal structures that have become available in the public domain since we solved this structure, *e.g.* pdb 6OSP and 6UX9.^19,21^ The latter structures differ, though, in that they do not show the re-positioning of the ‘activation loop’ that results in Leu230 becoming part of the binding pocket. There are four regions of significant disorder in the 36-bound structure: loop regions Ser126–Ala132, Thr216–Ala227, Glu289–Pro336 and Lys369–Ser386.

**Fig. 3:**
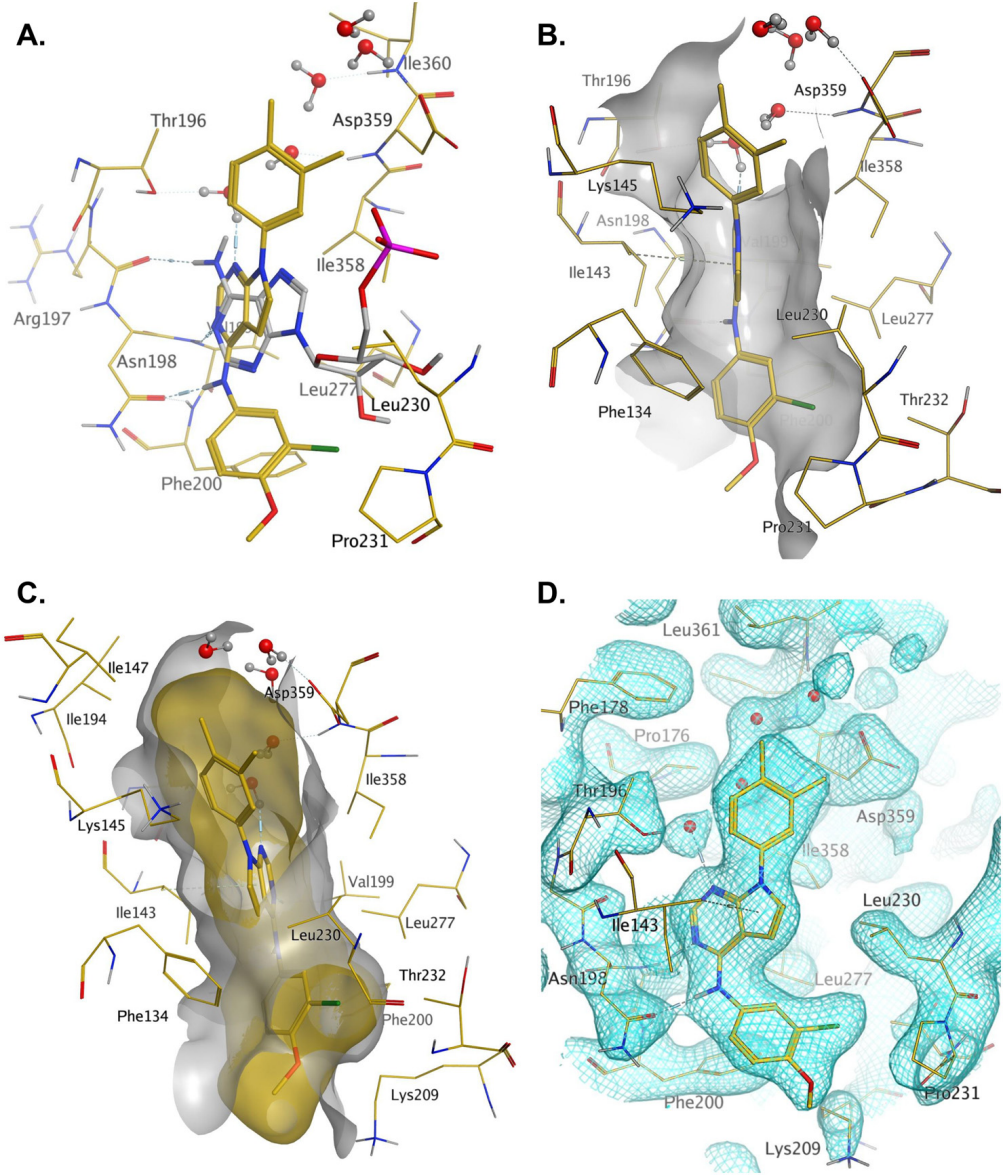
A: AMP co-factor of PI5P4Kβ (3X01, grey) superposed onto chain A of the 36–PI5P4Kα complex (pdb 8C8C, gold carbons); B: binding pocket in chain A of the 36–PI5P4Kα highlighting the hydrophobic cleft; C: binding pocket of the 36–PI5P4Kα complex with ligand and protein molecular surfaces; D: electron density at 1*σ* for protein residues and 36 in the binding pocket.

Compound 36 binds to the same pocket occupied by AMP in the PI5P4Kβ crystal structure (pdb 3X01; Fig. 3A). The binding pockets of the PI5P4Kβ crystal structure and the 36–PI5P4Kα structure are almost identical near the hinge, but the area where the sugar moiety binds in PI5P4Kβ is occupied by Leu230 in the 36–PI5P4Kα complex. 36 binds deeply in the hydrophobic cleft that forms the ATP binding site in lipid kinases. In particular, the compound binds in the largely hydrophobic pocket formed by Phe134, Ile143, Phe200, Leu230, Leu277 and Ile358 (Fig. 3A and B). The NH linker in 36 forms a hydrogen bond with the side-chain oxygen acceptor of Asn198, while N1 (see heterocycle numbering in Table 3) of compound 36's pyrrolopyrimidine forms a hydrogen bond with the main-chain nitrogen of Val199 (Fig. 2B). Furthermore, N3 of the pyrrolopyrimidine is seen to make a hydrogen bond interaction with an ordered water in the binding site. This water is positioned by further hydrogen bonding to the side-chain oxygen acceptor of Thr196 (Fig. 2B and 3A). This results in a repositioning of the water network at the top of the binding pocket, when compared with the apo structure 2YBX (Fig. 2B). As mentioned above, the side-chains of Phe134, Asn124, Lys145 and Lys209 have been re-orientated compared with the apo structure (Fig. 2B). These side-chain re-orientations result in a tight fit of the aniline aromatic ring, with the chlorine contacting the lipophilic part of the Lys209 and Thr232 side chains, as well as Leu230 and Leu277 (Fig. 3C). The methoxy group of 36 is solvent-accessible whereas the dimethylphenyl group partially fills a lipophilic pocket, with the remaining space of the pocket being occupied by a network of water molecules. The 4-methyl group makes contact with the side chains of Ile147, Ile194 and Lys145, whereas the 3-methyl group mainly interacts with Asp359. All atoms of the ligand are visible in the electron density (Fig. 3D).

## Discussion and conclusions

**Fig. 4:**
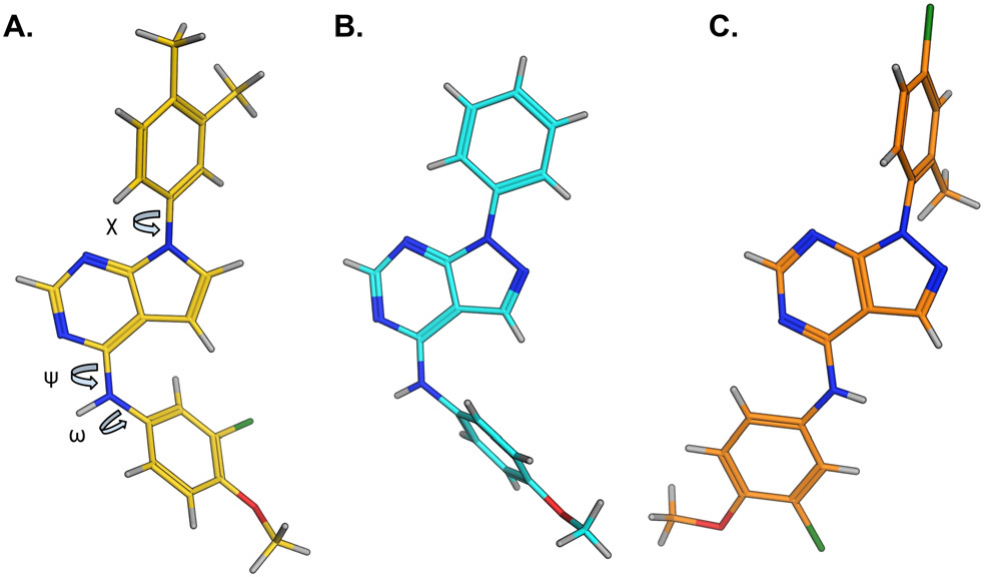
A) Conformation of 36 as bound in complex with PI5P4Kα (pdb 8C8C); B) X-ray conformation of a related compound (CSD: ETUWAU); C) small molecule X-ray structure of compound 20 (CSD: 223787). Key dihedral angles are indicated with Greek letters.

The effects of the modifications to the bicyclic core (Table 3) are generally explained very well by the crystal structure of the 36–PI5P4Kα complex. Both N1 and N3 are involved in hydrogen bonding, so changing either nitrogen to a carbon leads to loss of potency (38 and 37 respectively, *vs.*36). N8, on the other hand, is in a very lipophilic environment created by the Leu230 and Phe134 side-chains. Therefore, substituting this nitrogen for a carbon increases potency (26*vs.*36). The C7 methyl substitution of 40 prevents the ligand from adopting the binding conformation, so leads to loss of potency. The importance of the *meta*-Cl substitution for potency, evident from the data in Tables 1 and 2, arises from the interaction with Leu230, Thr232 and Lys209 in a very lipophilic pocket just large enough to fit the chlorine atom (Fig. 3C). However, 32 and 35 are inactive despite having the R^1^*meta*-Cl substituent. QM calculations suggest that these ligands favour a conformation where R^1^ and the pyrimidine core are in plane rather than at the 45° torsion angle (Fig. 4A: dihedral *ω*) seen for 36 in the complex. In this planar conformation the R^1^ group has rotated 180° around the NH–pyrimidine bond (dihedral *ψ*), so that the NH is no longer available for hinge-binding (as in Fig. 4C). QM calculations indicate that ligands with electron-donating substituents on R^1^ tend to favour the binding conformation seen for 36, and those with electron-withdrawing substituents favour the planar conformation (Fig. 4C) due to increased conjugation between the ring systems. A small molecule crystal structure of 20 shows that this compound adopts a non-hinge-binding conformation in its crystal form, whereas a similar structure in the CSD without the chlorines adopts the hinge-binding conformation (CSD-ETUWAU;^29^Fig. 4B). It is likely that conformational preference for a particular *ω* angle also plays a role. *Ab initio* optimization of 36 in the bound conformation changed *ω* angle from −45 to −60 degrees. This optimized ligand still fits well in the binding site. However, starting from the crystal structure conformation, compounds 32 and 35 optimized to *ω* angles of −83 and −96 degrees, respectively, which results in a clash with Phe134 when placed back in the binding site (see ESI† page S50). It appears that R^1^ requires a delicate balance between substituents that make hydrophobic contacts and those that support the binding conformation.

The R^2^ SAR in Table 2 shows that a *para*-methyl substitution is favourable, and the 8C8C structure shows that this methyl group makes hydrophobic contacts with the side-chains of Ile147, Ile194 and Leu361 (Fig. 3). Other *para* substituents appear not to be tolerated, *e.g.* the *para* chlorine of 19 and CF_3_ of 28. Given that the *p*-Cl group is tolerated on R^2^ of 20, where there is also an *ortho* substituent on the ring, the issue with the *p*-Cl group is likely to be again one of conformation of the ligand. The torsion angle between the core and R^2^ ring (Fig. 4A: dihedral *χ*) is 55° in 36 in complex with PI5P4Kα, while small molecule crystallography suggests that the lowest energy conformation for an unsubstituted phenyl at R^2^ is in plane with the core ring system (CSD-ETUWAU, Fig. 4B). *Ortho* substitution creates a favourable twist, whereas electron withdrawing groups and ring nitrogen atoms increase conjugation and thereby make this twist less energetically favourable.

In summary, a new PI5P4Kα-selective chemotype has been identified through virtual screening and optimised *via* iterations of design and synthesis to provide potent tool molecules to further probe the biology of this important target. ARUK2002821 (36) is a potent PI5P4Kα inhibitor (pIC_50_ = 8.0) which is selective *vs.* PI5P4Kβ and PI5P4Kγ and engages the target, in cells, at sub-micromolar concentrations. ADMET data has been provided for this tool molecule and others in the series, as well as an X-ray structure solved with this molecule as a complex with its PI5P4Kα target, so that researchers in the field of PI5P4K biology may have useful tools for further investigation.

## Experimental details

### Biochemical assays

Assays to determine kinase activity of PI5P4Ks in the presence of inhibitors using an ADP-Glo assay (Promega) were performed as described previously.^28^ Recombinant PI5P4K protein was prepared as described previously.^10^*E. coli* BL21(DE3) clones harbouring PI5P4Kα (*PIP4K2A*; UniGene 138363) or PI5P4Kβ (*PIP4K2B*; UniGene 171988), cloned into pGEX6P plasmid (Cytiva), were used to overexpress these proteins. Cultures were induced with 0.4 mM IPTG overnight and probe-sonicated in the presence of protease inhibitors. GST fusion protein was harvested using a GSTrap FF affinity column (Cytiva) and the GST tag removed *in situ* with 50 U of PreScission protease (Cytiva) for 4 h at 4 °C. The cleaved protein was further purified by size-exclusion chromatography (ÄKTA Pure, Cytiva). PI5P4Kγ+ protein (a genetically modified chimera of PI5P4Kγ with a specific activity close to that of the active PI5P4Kα isoform^10^) was generated from a pGEX6P construct harbouring PIP4K2C (UniGene 6280511) with a number of PI5P4Kα-like mutations (insertion of three amino acids (QAR) at 139 plus an additional 11 amino acid mutations: S132L, E133P, S134N, E135D, G136S, D141G, G142A, E156T, N198G, E199G and D200E) using the same protein purification method. The protein purity was confirmed by sodium dodecyl sulfate–polyacrylamide gel electrophoresis, and the concentration was determined by colorimetric assay (Bio-Rad).

Binding of compounds to PI5P4Kα in intact cells was assessed using an InCELL Pulse thermal stabilisation assay (DiscoverX). PI5P4Kα (*PIP4K2A*; UniGene 138363) was cloned into the pICP vector (DiscoverX) to allow overexpression of the ePL-tagged target. Hek293 cells stably expressing ePL-tagged PI5P4Kα were incubated with 25 nl of test compound in 100% DMSO in a black skirted PCR plate for 60 minutes at 38 °C. After incubation for 3 minutes at 46 °C, followed by cooling for 3 minutes at room temperature, 12 μl of EA-3 reagent (prepared as per the manufactures guidelines) was added to each well. The plate was then incubated for 60 minutes in the dark prior to luminescence reading on a Pherastar FSX plate reader (BMG Labtech).

### Data analysis

Statistical analysis was performed using nonparametric testing in Prism 8 (GraphPad). Activity pIC_50_ values and *in vivo* binding pEC_50_ values were estimated using a 4-parameter fit (Dotmatics).

### X-ray crystallography and structure determination

Crystallography was performed by Peak Proteins Ltd. Truncated human PI5P4Kα was expressed in *E.coli* BL21(DE3) Gold using a pET28b vector. Expression was induced using 0.1 mM IPTG and the cells cultured at 18 °C for 16 h before harvesting by centrifugation. The protein comprised of residues Asp35 to Thr406 with the region between residues 308–314 deleted. Purification of TEV-cleaved protein was by both affinity and size exclusion (Superdex 75) chromatography. The structure of the ligand complex was generated by co-crystallisation of human PI5P4Kα in the presence of 36. Purified protein (12 mg ml^−1^, 20 mM HEPES pH 7.5, 150 mM NaCl, 0.5 mM TCEP) was incubated with 3 mM 36 (from 100 mM stock in DMSO) for 1 hour at 4 °C. Crystals were grown by mixing protein in equal parts with 3% (w/v) PEG1000 3% (v/v) PEG600, 3% (v/v) PEG 500ME, 3% (v/v) PEG400, 40 mM calcium chloride, 40 mM sodium formate, 100 mM Tris–HCl pH 8.0 at 20 °C. For X-ray data collection they were flash-frozen and X-ray diffraction data were collected (I04 beamline, Diamond Light Source synchrotron facility, Oxford, UK) at 100 K. Data were processed using the XDS and Aimless software. The phase information necessary to determine and analyse the structure was obtained by molecular replacement (PHASER, CCP4) using the previously solved structure of a human PI5P4Kα (pdb 2YBX) as the search model. Subsequent model building and refinement was performed according to standard protocols with the software packages CCP4 and COOT. TLS refinement (REFMAC5, CCP4) has been carried out, which resulted in lower R-factors and higher quality of the electron density map. The ligand parameterisation and the generation of the corresponding library files was carried out with ACEDRG (CCP4). The water model was built with the “Find waters”-algorithm of COOT by putting water molecules in peaks of the *F*_o_–*F*_c_ map contoured at 3.0*σ* followed by refinement with REFMAC5 and checking all waters with the validation tool of COOT. The criteria for the list of suspicious waters were: B-factor greater 80 Å^2^, 2*F*_o_–*F*_c_ map less than 1.2*σ*, distance to closest contact less than 2.3 Å or more than 3.5 Å. The suspicious water molecules and those in the active site (distance to inhibitor less than 10 Å) were checked manually. The occupancy of side chains, which were in negative peaks in the *F*_o_–*F*_c_ map (contoured at −3.0*σ*), were set to zero and subsequently to 0.5 if a positive peak occurred after the next refinement cycle. Parameterisation and the generation of the corresponding library files was carried out with ACEDRG (CCP4).

The Ramachandran Plot of the final model shows 96.5% of all residues in the most favoured region, 3.3% in the additionally allowed region. Statistics of the final structure and the refinement process are listed in Table S12.†

### Computational modelling

The virtual screening procedure was performed as previously described.^25^

QM energy calculations were carried out with Jaguar (release 2016-3, Schrodinger, https://www.schrodinger.com). The structures were optimised at the M06-2X/cc-pVTZ(-f) theory level in solution (water : PBF) starting from both the 36 crystal complex conformation and the small molecule 20 crystal structure, and energies of the two resulting conformations for the same ligand were then compared.

## Abbreviations

ATP: Adenosine triphosphate
LE: Ligand efficiency
LLE: Lipophilic ligand efficiency
MLM: Mouse liver microsomes
PI5P4K: Phosphatidylinositol 5-phosphate 4-kinase
QM: Quantum mechanics
SAR: Structure–activity relationship
WT: Wild-type

## Author contributions

The manuscript was written through contributions of all authors. All authors have given approval to the final version of the manuscript. Stephen Andrews and Jonathan Clarke were programme leaders, John Skidmore offered further project leadership; together all three designed the project plan. Henriëtte Willems developed the virtual screening workflow, selected VS hits and analogues for purchasing and docked compounds. Simon Edwards designed and synthesised the compounds. Helen Boffey and Stephen Chawner conducted studies with imanixil. Christopher Green and Tamara Romero developed the InCELL Pulse assay for PI5P4Kα. David Winpenny and Christopher Green ran ADP-Glo and InCELL Pulse assays and screened the compounds. Tamara Romero produced recombinant PI5P4K protein.

## Conflicts of interest

There is no conflict of interest to declare.

## Supplementary Material

MD-014-D3MD00039G-s001

MD-014-D3MD00039G-s002
