# Supplementary material for: Identification of ARUK2002821 as an isoform-selective PI5P4Kα inhibitor
Source: RSC Med Chem. 2023 Apr 17;14(5):934–46. doi: 10.1039/d3md00039g (PMC10211317; doi:10.1039/d3md00039g)
Supplement: MD-014-D3MD00039G-s001 [file MD-014-D3MD00039G-s001.pdf]

Electronic Supplementary Material (ESI) for RSC Medicinal Chemistry.
This journal is © The Royal Society of Chemistry 2023

Supporting information

Identification of ARUK2002821 as an isoform-selective PI5P4Kα inhibitor

Henriëtte M. G. Willems, Simon Edwards, Helen K. Boffey, Stephen J. Chawner, Christopher Green, Tamara Romero, David Winpenny, John Skidmore, Jonathan H. Clarke and Stephen P. Andrews*

Affiliations

The ALBORADA Drug Discovery Institute, University of Cambridge, Island Research Building, Cambridge Biomedical Campus, Hills Road, Cambridge, CB2 0AH, United Kingdom

Corresponding Author

*E-mail: spa26@cam.ac.uk

Table of contents

	Page
Abbreviations	S2
Chemical synthesis	S3
General experimental	S3
General procedures 1 and 2	S4
Synthesis of Imanixil (**7**)	S5
Synthesis procedures	S9
Data for the commercially available compound **20**	S34
Spectra for key compounds **20**, **36**, **39**, **43** and **45**	S35
Commercially available test compounds	S39
**Table S1.** Analogues of **10** for which PI5P4Kα and γ pIC_{50}s were determined	S40
**Table S2.** Analogues of **11** for which PI5P4Kα and γ pIC_{50}s were determined	S40

**Table S3.** Additional commercial analogues of **8** for which PI5P4Kα and γ pIC_{50}s were determined	S40
**Table S4.** ADP-Glo PI5P4K activity data for Imanixil (**7**)	S41
**Table S5.** Crystallographic data and structure refinement for Imanixil (**7**)	S41
**Table S6.** Crystallographic bond length data for Imanixil (**7**)	S42
**Table S7.** Crystallographic bond angle data for Imanixil (**7**)	S42
**Table S8.** Lipid kinase panel screening for Imanixil (**7**) at DiscoverX	S43
**Table S9.** Lipid kinase panel screening for Imanixil (**7**) at MRC PPU International Centre for Kinase Profiling	S44
**Table S10.** Lipid kinase panel screening for compound **36**	S44
**Table S11.** Premier Screen kinase panel screening for compound **36**	S44
**Table S12.** Crystallographic data and structure refinement for **36**-PI5P4Kα	S47
**Table S13.** Crystallographic data and structure refinement for Compound **20**	S47
**Table S14.** Crystallographic bond length data for Compound **20**	S48
**Table S15.** Crystallographic bond angle data for Compound **20**	S48
ADMET experimental methods	S49
Consideration of dihedral angles for **32**, **35** and **36**	S50
References	S51

Abbreviations: DCE: 1,2-dichloroethane, DCM: dichloromethane, DiPEA: Diisopropylethylamine, DMF: dimethylformamide, eq: equivalents, IPA: *iso*-propanol, MeCN: acetonitrile, MeOH: methanol, min: minutes, NMP: N-methyl-2-pyrrolidone, rt: room temperature.

Chemical Synthesis

General Experimental

Imanixil (**7**) and precursors were prepared by Wuxi AppTec, China, other final compounds were purchased as indicated or synthesised at ALBORADA Drug Discovery Institute. Reagents and solvents were of commercially available reagent grade quality and used without further purification. Reactions requiring anhydrous conditions were carried out in oven dried glassware under an atmosphere of N_2. Reactions were monitored by thin-layer chromatography (TLC) on silica gel 60 F_{254} aluminium or glass supported sheets, or by liquid chromatography-mass spectrometry (LC-MS). Flash column chromatography was carried out on a Biotage Isolera One system using normal phase (SiO_2) or reverse phase (C18) cartridges. Compounds were loaded in solution or adsorbed onto Celite® 545 or ISOLUTE® HM-N, and eluted using a linear gradient of the specified solvents. Purification by C18 reverse phase HPLC was carried using an Agilent 1260 Infinity machine and a Waters XBridge BEH C18 OBD column (130 Å, 5 µm, 30 mm × 100 mm) with a linear gradient of H_2O (with 0.1% NH_3) and MeCN (with 0.1% NH_3). LCMS analysis was performed on a Waters Aquity HClass UPLC system with a Aquity QDa for mass detection. NMR spectra were recorded on a Bruker Advance III (^1H = 300 MHz, ^{19}F = 282 MHz) spectrometer using the requisite solvent as a reference for internal deuterium lock. The chemical shift data for each signal are given as δ chemical shift (multiplicity, *J* values in Hz, integration) in units of parts per million (ppm) relative to tetramethylsilane (TMS) where δH (TMS) = 0.00 ppm. The multiplicity of each signal is indicated by: s (singlet), d (doublet), t (triplet), q (quartet), quin (quintet), or m (multiplet). Signals from exchangeable protons are not always detected. UPLC analysis of final compounds was performed on a Waters Aquity HClass UPLC system and is reported as method name, retention time, UV % purity. The method parameters are as follows:

Method	Column	Additive	Flow rate	Gradient (time, %MeCN in H_2O)
A	BEH C18 (130 Å, 1.7 µm, 2.1 mm × 50 mm)	10 mM NH_3	0.6 mL/min	0 min, 5%; 0.8 min, 5%; 3.3 min, 95%; 4.3 min, 95%; 4.5 min, 5%; 5.5 min, 5%.
B	HSS C18 (100 Å, 1.8 µm, 2.1 mm × 50 mm)	0.1% HCO_2H	0.6 mL/min	0 min, 5%; 0.8 min, 5%; 3.3 min, 95%; 4.3 min, 95%; 4.5 min, 5%; 5.5 min, 5%.
C	BEH C18 (130 Å, 1.7 µm, 2.1 mm × 50 mm)	10 mM NH_3	0.6 mL/min	0 min, 5%; 0.8 min, 5%; 8.3 min, 95%; 9.3 min, 95%; 9.5 min, 5%; 10.5 min, 5%.
D	HSS C18 (100 Å, 1.8 µm, 2.1 mm × 50 mm)	0.1% HCO_2H	0.6 mL/min	0 min, 5%; 0.8 min, 5%; 8.3 min, 95%; 9.3 min, 95%; 9.5 min, 5%; 10.5 min, 5%.

General Procedure 1[1]: Hydrazone formation (Scheme 1, reaction a):

A solution of the requisite hydrazine (Caution! toxic; 1.0 eq) and the requisite aldehyde (1.05 eq) in DMF (Caution! Carcinogenic; 0.7 M) was stirred at rt for the stated period of time. The reaction was poured into cold $NaHCO_3$ solution, stirred for 15 min, filtered and dried *in vacuo*. The hydrazone thus obtained was used without further purification.

General Procedure 2[1,2]: Cyclisation/Displacement (Scheme 1, reaction b):

A microwave flask was charged with the requisite hydrazone (1.0 eq), dissolved in MeCN (1 M) and heated at 185 °C for the stated time. The reaction was cooled and the requisite amine (1.1 eq) and DiPEA (3.0 eq) were added. The mixture was then sealed and heated thermally for the stated time and temperature. Upon cooling to rt the reaction mixture was concentrated *in vacuo* and purified by the stated method.

Scheme 1:

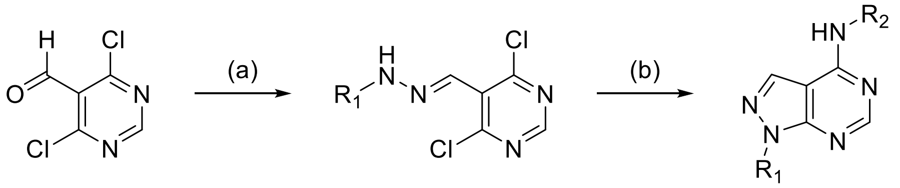

Reagents and conditions: (a) R1-$NHNH_2$, DMF, rt. (b) i) MeCN, 185 °C ii) R_2-NH_2, DiPEA, 125-140 °C.

Synthesis of Imanixil, 4-amino-2-(4,4-dimethyl-2-oxoimidazolidin-1-yl)-N-(3-(trifluoromethyl) phenyl)pyrimidine-5-carboxamide (7)[3,4]

2-Bromo-4,4-dimethyl-4,5-dihydrooxazole

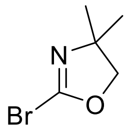

To a solution of 4,4-dimethyl-4,5-dihydrooxazole (19.0 g, 190 mmol, 1.0 eq) in THF (600 mL) was added *t*-BuLi (Caution! Flammable; 210 mmol, 1.1 eq) over a period of 5 min at -78 °C and the resulting yellow solution was stirred for 25 min. 1,2-Dibromo-1,1,2,2-tetrafluoro-ethane (Caution! toxic; 60 g, 230 mmol, 1.2 eq) was added and the solution was warmed to rt and then stirred for 6 h. The reaction mixture was quenched with sat. NH_4Cl and extracted with ethyl acetate (2 x 200 mL), dried (Na_2SO_4) and concentrated *in vacuo* to yield 2-bromo-4,4-dimethyl-4,5-dihydrooxazole (27.0 g, 152 mmol, 79% yield) as a yellow oil and was used in next step without further purification. ^{1}H NMR (400 MHz, $CDCl_3$) δ 4.15 (s, 2H), 1.36 (s, 6H).

1-Bromo-2-isocyanato-2-methylpropane

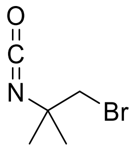

A solution of 2-bromo-4,4-dimethyl-4,5-dihydrooxazole (27.0 g, 152 mmol, 1.0 eq) in THF (300 mL) was stirred at 65 °C for 16 h. The reaction solution was concentrated *in vacuo* to yield 1-bromo-2-isocyanato-2-methylpropane (Caution! toxic; 26.0 g, 146 mmol, 96% yield) as a yellow oil which was used without further purification. ^{1}H NMR (400 MHz, $CDCl_3$) δ 3.48 (s, 2H), 1.50 (s, 6H).

Ethyl 2-(4,4-dimethyl-2-oxoimidazolidin-1-yl)-4-hydroxypyrimidine-5-carboxylate

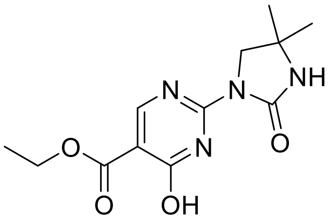

A suspension of ethyl 2-amino-4-hydroxypyrimidine-5-carboxylate (17.0 g, 93 mmol, 1.00 eq) in DMF (Caution! Carcinogenic; 200 mL) was heated to 100 °C with stirring for 1 h and then the mixture was cooled to 15 °C and stirred for a further 30 min at this temperature. 1-Bromo-2-isocyanato-2-methylpropane (Caution! toxic; 19.8 g, 111 mmol, 1.2 eq) was charged into the flask *via* syringe over 10 min. The reaction mixture was stirred at 100 °C for another 4 h. The reaction solution was poured into H_2O (1000 mL), stirred for 30 min and extracted with DCM (Caution! Carcinogenic; 3 x 200 mL). The combined organic phases were concentrated *in vacuo* and the crude product was crystallised from (petroleum ether:ethyl acetate = 3:1) (2 x 100 mL) to yield ethyl 2-(4,4-dimethyl-2-oxoimidazolidin-1-yl)-4-hydroxypyrimidine-5-carboxylate (13.2 g, 47.1 mmol, 50% yield) as a white solid. ^{1}H NMR (400 MHz, $CDCl_3$) δ 8.84 (s, 1H), 4.35 (q, J = 7.0 Hz, 2H), 3.86 (s, 2H), 1.42–1.35 (m, 9H) (exchangeable OH and NH protons not observed).

Ethyl 4-chloro-2-(4,4-dimethyl-2-oxoimidazolidin-1-yl)pyrimidine-5-carboxylate

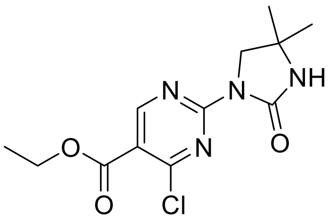

A solution of ethyl 2-(4,4-dimethyl-2-oxoimidazolidin-1-yl)-4-hydroxypyrimidine-5-carboxylate (13.2 g, 47 mmol, 1.0 eq) in thionyl chloride (Caution! toxic; 112 g, 942 mmol, 68 mL, 20 eq) was stirred at 100 °C for 4 h. The reaction solution was concentrated *in vacuo* to yield ethyl 4-chloro-2-(4,4-dimethyl-2-oxoimidazolidin-1-yl)pyrimidine-5-carboxylate (14.0 g, 47 mmol, 100% yield) as a white solid which was used without further purification.

Ethyl 4-amino-2-(4,4-dimethyl-2-oxoimidazolidin-1-yl)pyrimidine-5-carboxylate

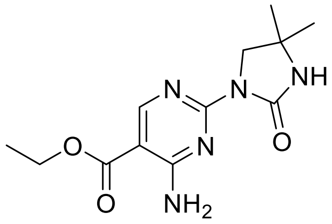

To a solution of ethyl 4-chloro-2-(4,4-dimethyl-2-oxoimidazolidin-1-yl)pyrimidine-5-carboxylate (12.2 g, 41 mmol, 1.0 eq) in THF (100 mL) was added a solution of NH_3 (696 mg, 41 mmol, 1.0 eq) in THF (100 mL). The reaction solution was stirred at 15 °C for 4 h, then was quenched with H_2O (100 mL) and extracted with DCM (Caution! Carcinogenic; 3 x 100 mL). The combined organic phases were washed with sat. aq. NaCl (100 mL) and concentrated *in vacuo* to yield ethyl 4-amino-2-(4,4-dimethyl-2-oxoimidazolidin-1-yl)pyrimidine-5-carboxylate (11.0 g, 39 mmol, 96% yield) as a white solid. ^{1}H NMR (400 MHz, MeOD-d_4) δ 8.71 (s, 1H), 5.51 (s, 1H), 4.35 (q, *J* = 7.0 Hz, 2H), 3.85 (s, 2H), 1.41–1.35 (m, 9H) (exchangeable 2 x NH protons not observed).

Lithium 4-amino-2-(4,4-dimethyl-2-oxoimidazolidin-1-yl)pyrimidine-5-carboxylate

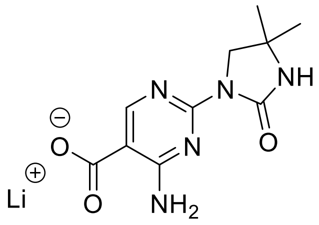

To a solution of ethyl 4-amino-2-(4,4-dimethyl-2-oxoimidazolidin-1-yl)pyrimidine-5-carboxylate (11.0 g, 39 mmol, 1.0 eq) in THF (200 mL) was added LiOH.H_2O (8.3 g, 197 mmol, 5.0 eq) in H_2O (20 mL). The mixture was stirred at 65 °C for 4 h, concentrated *in vacuo* and the product was recrystallised from H_2O (20 mL) to yield lithium 4-amino-2-(4,4-dimethyl-2-oxoimidazolidin-1-yl)pyrimidine-5-carboxylate (7.1 g, 28 mmol, 70% yield) as a white solid. ^{1}H NMR (400 MHz, D_2O) δ 8.38 (s, 1H), 3.69 (s, 2H), 1.22 (s, 6H) (exchangeable NH and NH_2 protons not observed).

4-Amino-2-(4,4-dimethyl-2-oxoimidazolidin-1-yl)pyrimidine-5-carbonyl chloride

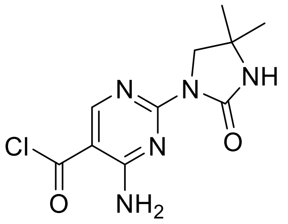

A mixture of lithium 4-amino-2-(4,4-dimethyl-2-oxoimidazolidin-1-yl)pyrimidine-5-carboxylate (6.5 g, 26 mmol, 1.0 eq) and $SOCl_2$ (Caution! toxic; 266 g, 162 mL, 2.2 mol, 87 eq) was stirred at 60 °C for 3 h. The reaction mixture was concentrated *in vacuo* to yield 4-amino-2-(4,4-dimethyl-2-oxoimidazolidin-1-yl)pyrimidine-5-carbonyl chloride (6.9 g, 26 mmol, 99% yield) as a yellow solid which was used without further purification.

Imanixil, 4-Amino-2-(4,4-dimethyl-2-oxoimidazolidin-1-yl)-N-(3-(trifluoromethyl)phenyl)pyrimidine-5-carboxamide hydrochloride (**7**)

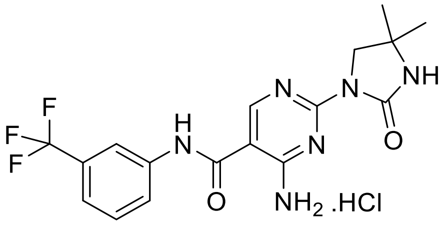

To a solution of 4-amino-2-(4,4-dimethyl-2-oxoimidazolidin-1-yl)pyrimidine-5-carbonyl chloride (6.0 g, 22 mmol, 1.0 eq) in DCM (Caution! Carcinogenic; 60 mL) was added 3-(trifluoromethyl)aniline (7.2 g, 5.6 mL, 45 mmol, 2.0 eq) dropwise, and the reaction solution was stirred at 15 °C for 0.5 h. The reaction solution was concentrated *in vacuo* and the product was recrystallised from 50 mL H_2O/MeCN (1:1) and 50 mL of petroleum ether/ethyl acetate (1:1) to yield Imanixil (**7**) (5.1 g, 13 mmol, 58% yield) as a hydrochloride salt, as a light-yellow solid. MS (ESI+) m/z calcd for $C_{17}H_{18}F_3N_6O_2^+$ $[M + H]^+$ 395.1, found 395.0. ^{1}H NMR (300 MHz, DMSO-d_6) δ 13.04 (s, 1H), 11.33 (s, 1H), 9.24 (s, 1H), 8.79 (s, 1H), 8.68 (s, 1H), 8.64 (s, 1H), 8.22 (s, 1H), 7.97 (d, *J* = 8.4 Hz, 1H), 7.62 (t, *J* = 8.0 Hz, 1H), 7.50 (d, *J* = 7.8 Hz, 1H), 3.74 (s, 2H), 1.33 (s, 6H). ^{13}C NMR (75 MHz, DMSO-d_6) δ 163.28, 162.63, 155.35, 150.75, 146.55, 139.65, 130.43, 129.76 (q, *J* = 31.2 Hz), 126.36, 124.72, 122.75, 121.01, 117.27 (q, *J* = 4.3 Hz), 104.65, 56.02, 52.12, 28.58. ^{19}F NMR (282 MHz, DMSO-d_6) δ -61.26. UPLC analysis (method D), 3.39 min, 100% purity.

Synthesis of N-(3-chloro-4-methoxy-phenyl)-1-(6-methyl-3-pyridyl)pyrazolo[3,4-d]pyrimidin-4-amine (27)

(E)-4,6-dichloro-5-((2-(6-methylpyridin-3-yl)hydrazineylidene)methyl)pyrimidine

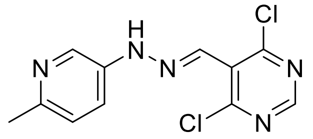

Following General Procedure 1, (6-methyl-3-pyridyl)hydrazine hydrochloride (Caution! toxic; 168 mg, 0.90 mmol) and 4,6-dichloro-5-pyrimidinecarbaldehyde (168 mg, 0.95 mmol) were stirred in DMF (Caution! Carcinogenic; 3.0 mL) overnight, yielding (E)-4,6-dichloro-5-((2-(6-methylpyridin-3-yl)hydrazineylidene)methyl)pyrimidine (200 mg, 0.71 mmol, 79% yield).

N-(3-chloro-4-methoxy-phenyl)-1-(6-methyl-3-pyridyl)pyrazolo[3,4-d]pyrimidin-4-amine (**27**)

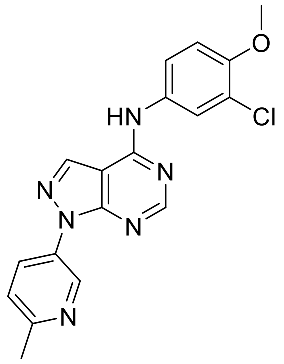

Following General Procedure 2, (E)-4,6-dichloro-5-((2-(6-methylpyridin-3-yl)hydrazineylidene)methyl)pyrimidine (100 mg, 0.35 mmol) in MeCN (3.0 mL) was stirred at 185 °C for 20 min, then 3-chloro-4-methoxyaniline (61 mg, 0.39 mmol) and DiPEA (137 mg, 0.18 mL, 1.1 mmol) were added and the mixture was stirred at 90 °C for 6 h. Purification by preparatory HPLC (elution gradient: 40-80% MeCN in H_2O with 0.1% NH_3) yielded N-(3-chloro-4-methoxy-phenyl)-1-(6-methyl-3-pyridyl)pyrazolo[3,4-d]pyrimidin-4-amine **27** (19 mg, 0.052 mmol, 15% yield) as a white solid. MS (ESI+) m/z calcd for $C_{18}H_{16}ClN_6O^+$ $[M + H]^+$ 367.1, found 367.2. ^1H NMR (300 MHz, DMSO-d_6) δ 10.28 (s, 1H), 9.23 (d, J = 2.5 Hz, 1H), 8.56 (s, 2H), 8.45 (dd, J = 8.4, 2.7 Hz, 1H), 8.08 (d, J = 2.5 Hz, 1H), 7.67 (dd, J = 8.9, 2.6 Hz, 1H), 7.48 (d, J = 8.5 Hz, 1H), 7.23 (d, J = 9.0 Hz, 1H), 3.88 (s, 3H), 2.55 (s, 3H). UPLC analysis (method D), 1.75 min, 100% purity.

Synthesis of N-(3-chloro-4-methoxy-phenyl)-1-[5-(trifluoromethyl)-2-pyridyl]pyrazolo[3,4-d] pyrimidin-4-amine (28)

(E)-4,6-dichloro-5-((2-(5-(trifluoromethyl)pyridin-2-yl)hydrazineylidene)methyl)pyrimidine

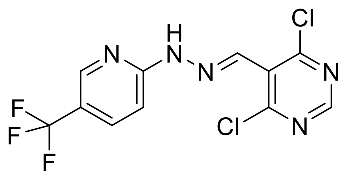

Following General Procedure 1, 2-hydrazinyl-5-(trifluoromethyl)pyridine (500 mg, 2.8 mmol) and 4,6-dichloro-5-pyrimidinecarbaldehyde (525 mg, 3.0 mmol) were stirred in DMF (Caution! Carcinogenic; 3.0 mL) overnight to yield (E)-4,6-dichloro-5-((2-(5-(trifluoromethyl)pyridin-2-yl)hydrazineylidene)methyl)pyrimidine (660 mg, 2.0 mmol, 70% yield).

N-(3-chloro-4-methoxy-phenyl)-1-[5-(trifluoromethyl)-2-pyridyl]pyrazolo[3,4-d]pyrimidin-4-amine (**28**)

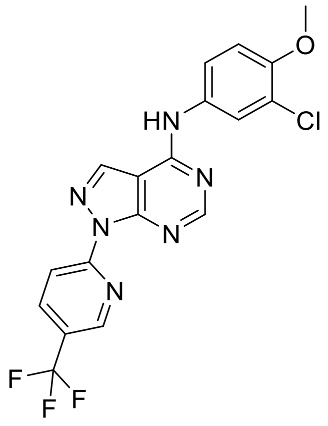

Following General Procedure 2, (E)-4,6-dichloro-5-((2-(5-(trifluoromethyl)pyridin-2-yl)hydrazineylidene)methyl)pyrimidine (100 mg, 0.29 mmol) in MeCN (4.0 mL) was heated at 185 ^{o}C for 25 min, then 3-chloro-4-methoxyaniline (51 mg, 0.38 mmol) and DiPEA (115 mg, 0.89 mmol) were added and the mixture was stirred at 85 ^{o}C overnight. Purification by preparatory HPLC (elution gradient: 40-80% MeCN in H_2O with 0.1% NH_3) yielded N-(3-chloro-4-methoxy-phenyl)-1-[5-(trifluoromethyl)-2-pyridyl]pyrazolo[3,4-d]pyrimidin-4-amine **28** (49 mg, 0.14 mmol, 39% yield) as a white solid. MS (ESI+) m/z calcd for $C_{18}H_{13}ClF_3N_6O^+$ [M + H]$^+$ 421.1, found 421.2. ^{1}H NMR (300 MHz, DMSO-d_6) δ 10.30 (s, 1H), 9.06 – 8.98 (m, 1H), 8.64 – 8.40 (m, 4H),

8.07 (d, *J* = 2.4 Hz, 1H), 7.66 (dd, *J* = 8.9, 2.6 Hz, 1H), 7.22 (d, *J* = 9.0 Hz, 1H), 3.88 (s, 3H). UPLC analysis (method C), 5.52 min, 100% purity.

Synthesis of N-(3-chloro-4-methoxy-phenyl)-1-cyclopropyl-pyrazolo[3,4-d]pyrimidin-4-amine (29)

(E)-4,6-dichloro-5-((2-cyclopropylhydrazineylidene)methyl)pyrimidine

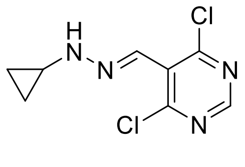

Following General Procedure 1, cyclopropylhydrazine hydrochloride (Caution! toxic; 1.0 g, 9.2 mmol) and 4,6-dichloro-5-pyrimidinecarbaldehyde (1.7 g, 9.7 mmol) were stirred in DMF (Caution! Carcinogenic; 10 mL) overnight. The crude product was purified by silica column chromatography (elution gradient: 0-100% ethyl acetate in hexane) to yield (E)-4,6-dichloro-5-((2-cyclopropylhydrazineylidene)methyl)pyrimidine (351 mg, 1.5 mmol, 17% yield) as a white powder.

N-(3-chloro-4-methoxy-phenyl)-1-cyclopropyl-pyrazolo[3,4-d]pyrimidin-4-amine (**29**)

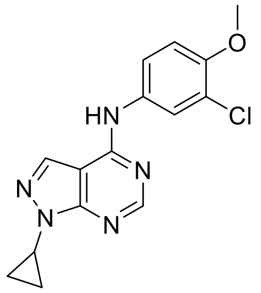

Following General Procedure 2, (E)-4,6-dichloro-5-((2-cyclopropylhydrazineylidene)methyl)pyrimidine (115 mg, 0.50 mmol) in MeCN (3.0 mL) was heated at 185 ºC for 20 min, then 3-chloro-4-methoxyaniline (86 mg, 0.55 mmol) and DiPEA (193 mg, 0.26 mL, 1.5 mmol) were added and the mixture was stirred at 120 ºC for 18 h. The reaction mixture was poured onto an SCX-2 cartridge, washed with DCM (Caution! Carcinogenic) and MeOH and eluted with 0.5 N methanolic ammonia. The basic fraction was concentrated *in vacuo* and further purified by preparatory HPLC (elution gradient: 20-60% MeCN in H_2O with 0.1% NH_3) to yield N-(3-chloro-4-methoxy-phenyl)-1-cyclopropyl-pyrazolo[3,4-d]pyrimidin-4-amine **29** (15 mg, 0.048 mmol, 10% yield) as a white solid.

MS (ESI+) m/z calcd for $C_{15}H_{15}ClN_5O^+$ $[M + H]^+$ 316.1, found 316.1. ^{1}H NMR (300 MHz, DMSO-d_6) δ 10.03 (s, 1H), 8.43 (s, 1H), 8.15 (s, 1H), 8.06 (d, *J* = 2.5 Hz, 1H), 7.65 (dd, *J* = 9.0, 2.6 Hz, 1H), 7.19 (d, *J* = 9.0 Hz, 1H), 3.86 (s, 3H), 3.32 (s, 1H), 1.24 – 1.13 (m, 2H), 1.13 – 1.04 (m, 2H). UPLC analysis (method D), 4.56 min, 96% purity.

Synthesis of 1-(2,4-dimethylphenyl)-N-(4-methoxyphenyl)pyrazolo[3,4-d]pyrimidin-4-amine (31) and N-[3-[[1-(2,4-dimethylphenyl)pyrazolo[3,4-d]pyrimidin-4-yl]amino]phenyl]acetamide (33)

(E)-4,6-dichloro-5-((2-(2,4-dimethylphenyl)hydrazineylidene)methyl)pyrimidine

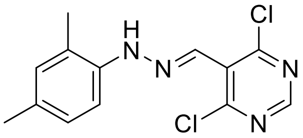

Following General Procedure 1, 2,4-dimethylphenyl hydrazine hydrochloride (Caution! toxic; 1.1 g, 6.4 mmol) and 4,6-dichloro-5-pyrimidinecarbaldehyde (1.2 g, 6.7 mmol) were stirred in DMF (Caution! Carcinogenic; 10 mL) overnight to yield (E)-4,6-dichloro-5-((2-(2,4-dimethylphenyl)hydrazineylidene)methyl)pyrimidine (1.62 g, 5.5 mmol, 86% yield).

1-(2,4-dimethylphenyl)-N-(4-methoxyphenyl)pyrazolo[3,4-d]pyrimidin-4-amine (**31**)

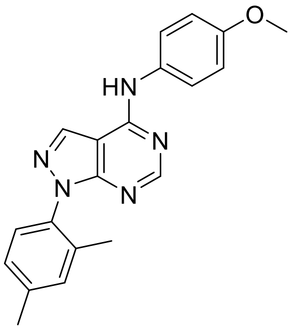

Following General Procedure 2, (E)-4,6-dichloro-5-((2-(2,4-dimethylphenyl)hydrazineylidene)methyl)pyrimidine (100 mg, 0.34 mmol) in MeCN (4.0 mL) was heated at 185 ^oC for 20 min, then *p*-anisidine (46 mg, 0.37 mmol) and DiPEA (131 mg, 0.18 mL, 1.0 mmol) were added and the mixture was stirred at 90 ^oC overnight. Purification by preparatory HPLC (elution gradient: 5-95% MeCN in

H_2O with 0.1% NH_3) yielded 1-(2,4-dimethylphenyl)-N-(4-methoxyphenyl)pyrazolo[3,4-d]pyrimidin-4-amine **31** (79 mg, 0.23 mmol, 68% yield) as a white solid. MS (ESI+) m/z calcd for $C_{20}H_{20}N_5O^+$ $[M + H]^+$ 346.2, found 346.3. ^1H NMR (300 MHz, DMSO-d_6) δ 10.08 (s, 1H), 8.42 (s, 1H), 8.32 (s, 1H), 7.69 (d, J = 8.3 Hz, 2H), 7.30 – 7.13 (m, 3H), 7.05 – 6.96 (m, 2H), 3.78 (s, 3H), 2.38 (s, 3H), 2.02 (s, 3H). UPLC analysis (method C), 5.22 min, 100% purity.

N-[3-[[1-(2,4-dimethylphenyl)pyrazolo[3,4-d]pyrimidin-4-yl]amino]phenyl]acetamide (**33**)

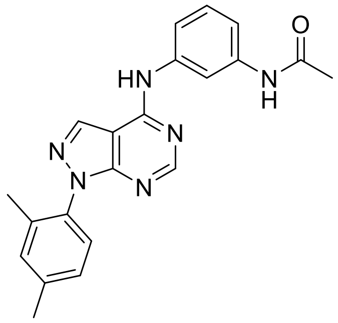

Following General Procedure 2, (E)-4,6-dichloro-5-((2-(2,4-dimethylphenyl)hydrazineylidene)methyl)pyrimidine (100 mg, 0.34 mmol) in MeCN (3.0 mL) was heated at 185 °C for 20 min, then N-(3-aminophenyl)acetamide (56 mg, 0.37 mmol) and DiPEA (131 mg, 0.18 mL, 1.0 mmol) were added and the mixture stirred at 120 °C overnight. Purification by preparatory HPLC (elution gradient: 40-80% MeCN in H_2O with 0.1% NH_3) yielded N-[3-[[1-(2,4-dimethylphenyl)pyrazolo[3,4-d]pyrimidin-4-yl]amino]phenyl]acetamide **33** (55 mg, 0.15 mmol, 44% yield) as a white solid. MS (ESI+) m/z calcd for $C_{21}H_{21}N_6O^+$ $[M + H]^+$ 373.2, found 373.3. ^1H NMR (300 MHz, DMSO-d_6) δ 10.22 (s, 1H), 10.03 (s, 1H), 8.51 (s, 1H), 8.39 (s, 1H), 8.16 (s, 1H), 7.68 – 7.59 (m, 1H), 7.36 – 7.24 (m, 4H), 7.21 – 7.16 (m, 1H), 2.38 (s, 3H), 2.07 (s, 3H), 2.03 (s, 3H). UPLC analysis (method C), 4.54 min, 99% purity.

Synthesis of 34 and 35 from (E)-4,6-dichloro-5-((2-(3,4-dimethylphenyl)hydrazineylidene)methyl) pyrimidine

(E)-4,6-dichloro-5-((2-(3,4-dimethylphenyl)hydrazineylidene)methyl)pyrimidine

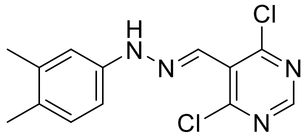

Following General Procedure 1, 3,4-dimethylphenyl hydrazine hydrochloride (Caution! toxic; 1.0 g, 5.8 mmol) and 4,6-dichloro-5-pyrimidinecarbaldehyde (1.08 g, 6.1 mmol) were stirred in DMF (Caution! Carcinogenic; 10 mL) overnight to yield (E)-4,6-dichloro-5-((2-(3,4-dimethylphenyl)hydrazineylidene)methyl)pyrimidine (1.5 g, 5.1 mmol, 88% yield).

1-(3,4-Dimethylphenyl)-N-(6-methoxy-3-pyridyl)pyrazolo[3,4-d]pyrimidin-4-amine (**34**)

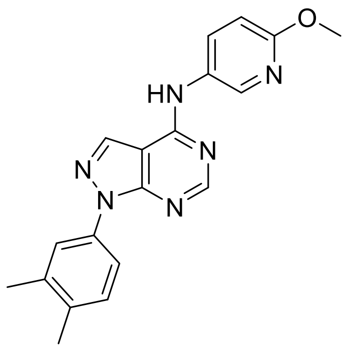

Following General Procedure 2, (E)-4,6-dichloro-5-((2-(3,4-dimethylphenyl)hydrazineylidene)methyl)pyrimidine (100 mg, 0.34 mmol) in MeCN (4.0 mL) was heated at 185 ^{o}C for 20 min, then 6-methoxypyridin-3-amine (46 mg, 0.37 mmol) and DiPEA (131 mg, 0.18 mL, 1.0 mmol) were added and the mixture was heated at 110 ^{o}C for 48 h. Purification by preparatory HPLC (elution gradient: 40-80% MeCN in H_2O with 0.1% NH_3) yielded 1-(3,4-dimethylphenyl)-N-(6-methoxy-3-pyridyl)pyrazolo[3,4-d]pyrimidin-4-amine **34** (17 mg, 0.049 mmol, 14% yield) as a white solid. MS (ESI+) m/z calcd for $C_{19}H_{19}N_6O^+$ $[M + H]^+$ 347.2, found 347.3. ^{1}H NMR (300 MHz, DMSO-d_6) δ 10.25 (s, 1H), 8.52 (s, 1H), 8.50 (d, J = 7.5 Hz, 1H), 8.14 (dd, J = 8.9, 2.8 Hz, 1H), 8.00 – 7.85 (m, 2H), 7.32 (d, J = 8.2 Hz, 1H), 6.91 (dd, J = 8.8, 0.7 Hz, 1H), 3.88 (s, 3H), 2.33 (s, 3H), 2.29 (s, 3H) (exchangeable NH proton not observed). UPLC analysis (method C), 5.47 min, 98% purity.

N-(5-chloro-6-methoxy-3-pyridyl)-1-(3,4-dimethylphenyl)pyrazolo[3,4-d]pyrimidin-4-amine (**35**)

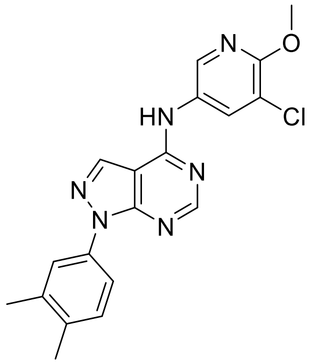

A microwave vial was charged with (E)-4,6-dichloro-5-((2-(3,4-dimethylphenyl)hydrazineylidene)methyl)pyrimidine (100 mg, 0.34 mmol), dissolved in MeCN (4.0 mL) and heated at 185 °C for 20 min. The reaction was cooled to rt and 5-chloro-6-methoxypyridin-3-amine (59 mg, 0.37 mmol) and DiPEA (131 mg, 1.0 mmol) were added. The mixture was then sealed and heated at 110 °C for 48 h. Upon cooling to rt the reaction mixture was concentrated *in vacuo* and purified by preparatory HPLC (elution gradient: 40-80% MeCN in H_2O with 0.1% NH_3) to yield N-(5-chloro-6-methoxy-3-pyridyl)-1-(3,4-dimethylphenyl)pyrazolo[3,4-d]pyrimidin-4-amine **35** (23 mg, 0.060 mmol, 18% yield) as a white solid. MS (ESI+) m/z calcd for $C_{19}H_{18}ClN_6O^+$ $[M + H]^+$ 381.1, found 381.3. ^{1}H NMR (300 MHz, DMSO-d_6) δ 10.39 (s, 1H), 8.58 – 8.44 (m, 4H), 7.97 – 7.87 (m, 2H), 7.32 (d, J = 8.3 Hz, 1H), 3.97 (s, 3H), 2.33 (s, 3H), 2.29 (s, 3H). UPLC analysis (method D), 6.56 min, 98% purity.

Synthesis of N-(3-chloro-4-methoxy-phenyl)-7-(3,4-dimethylphenyl)pyrrolo[2,3-d]pyrimidin-4-amine (36)

N-(3-chloro-4-methoxy-phenyl)-7H-pyrrolo[2,3-d]pyrimidin-4-amine

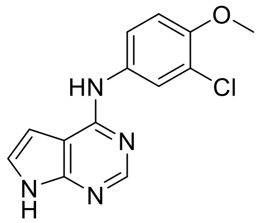

A microwave vial was charged with 6-chloro-7-deazapurine (500 mg, 3.3 mmol), 3-chloro-4-methoxyaniline (770 mg, 4.9 mmol) and DiPEA (631 mg, 4.9 mmol), dissolved in 1-butanol (5.0 mL) and heated at 130 °C for 40 h. Upon cooling to rt the reaction mixture was concentrated *in vacuo* and purified by silica gel chromatography

(elution gradient: 0-20% MeOH in DCM; Caution! Carcinogenic) to yield N-(3-chloro-4-methoxy-phenyl)-7H-pyrrolo[2,3-d]pyrimidin-4-amine (267 mg, 0.97 mmol, 30% yield) as a white solid. MS (ESI+) m/z calcd for $C_{13}H_{12}ClN_4O^+$ $[M + H]^+$ 275.1, found 275.1. UPLC analysis (method A), 2.59 min.

N-(3-chloro-4-methoxy-phenyl)-7-(3,4-dimethylphenyl)pyrrolo[2,3-d]pyrimidin-4-amine (**36**)

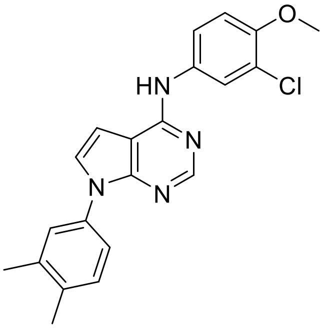

A microwave vial was charged with N-(3-chloro-4-methoxy-phenyl)-7H-pyrrolo[2,3-d]pyrimidin-4-amine (75 mg, 0.27 mmol), 4-iodo-*o*-xylene (76 mg, 0.33 mmol), potassium phosphate tribasic (127 mg, 0.60 mmol), *trans* N,N'-dimethylcyclohexane-1,2-diamine (5.8 mg, 0.041 mmol) and copper(l) iodide (3.6 mg 0.019 mmol), dissolved in 1,4-dioxane (Caution! Carcinogenic; 3.0 mL), sealed and heated at 100 ^{o}C for 18 h. Upon cooling to rt the reaction mixture was poured onto an SCX-2 cartridge, washed with DCM (Caution! Carcinogenic) and methanol, then eluted with 0.5 N methanolic ammonia. The basic fraction was concentrated *in vacuo* and purified by preparatory HPLC (elution gradient: 40-80% MeCN in H_2O with 0.1% NH_3) to yield N-(3-chloro-4-methoxy-phenyl)-7-(3,4-dimethylphenyl)pyrrolo[2,3-d]pyrimidin-4-amine **36** (36 mg, 0.095 mmol, 34% yield) as a white solid. MS (ESI+) m/z calcd for $C_{21}H_{20}ClN_4O^+$ $[M + H]^+$ 379.1, found 379.2. ^{1}H NMR (300 MHz, DMSO-d_6) δ 9.47 (s, 1H), 8.36 (s, 1H), 8.11 (d, J = 2.6 Hz, 1H), 7.72 (dd, J = 9.0, 2.6 Hz, 1H), 7.65 (d, J = 3.7 Hz, 1H), 7.59 (d, J = 2.0 Hz, 1H), 7.52 (dd, J = 8.2, 2.3 Hz, 1H), 7.29 (d, J = 8.3 Hz, 1H), 7.17 (d, J = 9.1 Hz, 1H), 6.97 (d, J = 3.7 Hz, 1H), 3.85 (s, 3H), 2.31 (s, 3H), 2.28 (s, 3H). ^{13}C NMR (75 MHz, DMSO) δ 153.75, 151.56, 149.99, 149.43, 137.23, 135.44, 134.62, 133.84, 130.02, 125.01, 124.49, 122.11, 120.91, 120.43, 120.32, 112.88, 104.58, 100.04, 56.22, 19.60, 18.98. UPLC analysis (method C), 6.27 min, 100% purity.

Synthesis of N-(3-chloro-4-methoxy-phenyl)-1-(3,4-dimethylphenyl)pyrrolo[3,2-c]pyridin-4-amine (37)

4-chloro-1-(3,4-dimethylphenyl)pyrrolo[3,2-c]pyridine

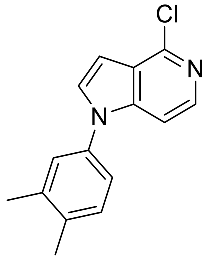

A microwave vial was charged with 4-chloro-1H-pyrrolo[3,2-c]pyridine (200 mg, 1.3 mmol), 4-iodo-*o*-xylene (335 mg, 1.4 mmol), potassium phosphate tribasic (611 mg, 2.9 mmol), *trans* N,N'-dimethylcyclohexane-1,2-diamine (37 mg, 0.26 mmol) and copper(l) iodide (25 mg, 0.13 mmol), dissolved in 1,4-dioxane (Caution! Carcinogenic; 4.0 mL), sealed and heated at 120 ºC for 18 h. Upon cooling to rt the reaction mixture was concentrated *in vacuo* and purified by silica gel chromatography (elution gradient: 0-50% ethyl acetate in hexane) to yield 4-chloro-1-(3,4-dimethylphenyl)pyrrolo[3,2-c]pyridine (185 mg, 0.72 mmol, 55% yield) as a white solid. MS (ESI+) m/z calcd for $C_{15}H_{14}ClN_2^+$ $[M + H]^+$ 257.1, found 257.1. UPLC analysis (method A), 3.51 min.

N-(3-chloro-4-methoxy-phenyl)-1-(3,4-dimethylphenyl)pyrrolo[3,2-c]pyridin-4-amine (**37**)

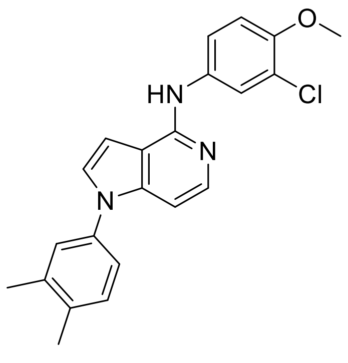

A microwave vial was charged with 4-chloro-1-(3,4-dimethylphenyl)pyrrolo[3,2-c]pyridine (80 mg, 0.31 mmol), 3-chloro-4-methoxyaniline (59 mg, 0.31 mmol) and 4 N HCl in dioxane (Caution! Carcinogenic; 0.078 mL, 0.31 mmol), dissolved in 1,4-dioxane (Caution! Carcinogenic; 3.0 mL) and heated at 120 ºC for 42 h. Upon cooling to rt the reaction mixture was concentrated *in vacuo* and purified by preparatory HPLC (elution gradient: 50-90% MeCN in H_2O with 0.1% NH_3) to yield N-(3-chloro-4-methoxy-phenyl)-1-(3,4-dimethylphenyl)pyrrolo[3,2-c]pyridin-4-amine **37** (75 mg, 0.20 mmol, 64 % yield) as a white solid. MS (ESI+) m/z calcd for $C_{22}H_{21}ClN_3O^+$

[M + H]$^+$ 378.1, found 378.2. ^{1}H NMR (300 MHz, DMSO-d_6) δ 8.82 (s, 1H), 8.19 (d, *J* = 2.6 Hz, 1H), 7.87 – 7.72 (m, 2H), 7.53 (d, *J* = 3.3 Hz, 1H), 7.39 – 7.22 (m, 3H), 7.16 – 7.07 (m, 2H), 6.92 (dd, *J* = 6.0, 0.8 Hz, 1H), 3.82 (s, 3H), 2.32 (s, 3H), 2.30 (s, 3H) UPLC analysis (method C), 6.84 min, 97% purity.

Synthesis of N-(3-chloro-4-methoxy-phenyl)-1-(3,4-dimethylphenyl)pyrrolo[2,3-b]pyridin-4-amine (38)

4-Chloro-1-(3,4-dimethylphenyl)pyrrolo[2,3-b]pyridine

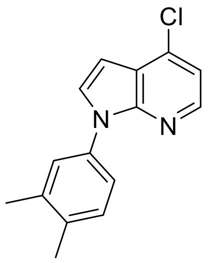

A microwave vial was charged with 4-chloro-1H-pyrrolo[2,3-b]pyridine (500 mg, 3.3 mmol), 4-iodo-*o*-xylene (907 mg, 3.9 mmol), potassium phosphate tribasic (1.5 g, 7.2 mmol), *trans* N,N'-dimethylcyclohexane-1,2-diamine (46 mg, 0.33 mmol), copper(l) iodide (43 mg, 0.23 mmol) and 1,4-dioxane (Caution! Carcinogenic; 10 mL), sealed and heated at 120 ºC for 18 h. Upon cooling rt the reaction mixture was concentrated *in vacuo* and purified by silica gel chromatography (elution gradient: 0-50% ethyl acetate in hexane) to yield 4-chloro-1-(3,4-dimethylphenyl)pyrrolo[2,3-b]pyridine (815 mg, 3.2 mmol, 97% yield) as a white solid. MS (ESI+) m/z calcd for $C_{22}H_{21}ClN_3O^+$ [M + H]$^+$ 257.1, found 257.1. UPLC analysis (method A), 3.67 min.

N-(3-chloro-4-methoxy-phenyl)-1-(3,4-dimethylphenyl)pyrrolo[2,3-b]pyridin-4-amine (**38**)

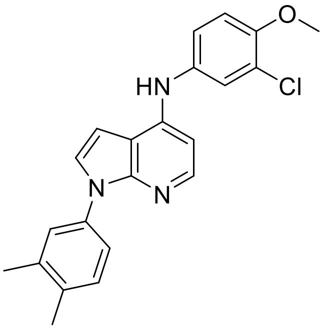

A microwave vial was charged with 4-chloro-1-(3,4-dimethylphenyl)pyrrolo[2,3-b]pyridine (100 mg, 0.39 mmol), 3-chloro-4-methoxyaniline (77 mg, 0.49 mmol), Xantphos (23 mg, 0.039 mmol), tris(dibenzylideneacetone)dipalladium(0) (18 mg, 0.019 mmol), Cs_2CO_3 (277 mg, 0.86 mmol) and 1,4-dioxane (Caution! Carcinogenic; 3.0 mL), sealed and degassed with bubbling nitrogen for 5 min. The reaction was heated at 125 ºC for 75 min. Upon cooling to rt the reaction mixture was poured onto an SCX-2 cartridge, washed with DCM (Caution! Carcinogenic) and methanol, and eluted with 0.5 N methanolic ammonia. The basic fraction was concentrated *in vacuo* and purified by preparatory HPLC (elution gradient: 5-95% MeCN in H_2O with 0.1% NH_3) to yield N-(3-chloro-4-methoxy-phenyl)-1-(3,4-dimethylphenyl)pyrrolo[2,3-b]pyridin-4-amine **38** (28 mg, 0.074 mmol, 19% yield) as a white solid. MS (ESI+) m/z calcd for $C_{22}H_{21}ClN_3O^+$ $[M + H]^+$ 378.1, found 378.2. ^1H NMR (300 MHz, DMSO-d_6) δ 8.70 (s, 1H), 7.96 (d, *J* = 5.5 Hz, 1H), 7.59 (ddd, *J* = 13.8, 7.5, 2.3 Hz, 3H), 7.36 (d, *J* = 2.5 Hz, 1H), 7.32 – 7.23 (m, 2H), 7.18 (d, *J* = 8.9 Hz, 1H), 6.81 (d, *J* = 3.7 Hz, 1H), 6.62 (d, *J* = 5.5 Hz, 1H), 3.87 (s, 3H), 2.30 (s, 3H), 2.27 (s, 3H). UPLC analysis (method C), 6.52 min, 97% purity.

Synthesis of 4-(3-chloro-4-methoxy-anilino)-7-(3,4-dimethylphenyl)-5H-pyrrolo[2,3-d]pyrimidin-6-one (39)

4-chloro-7-(3,4-dimethylphenyl)-5H-pyrrolo[2,3-d]pyrimidin-6-one

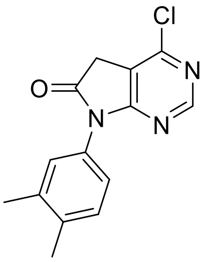

A microwave vial was charged with methyl (4,6-dichloro-5-pyrimidinyl)acetate (750 mg, 3.4 mmol), 3,4-dimethylaniline (452 mg, 3.7 mmol) and DiPEA (1.17 g, 1.58 mL, 10 mmol), dissolved in MeCN (8.0 mL) and heated at 170 °C for 90 min. Upon cooling to rt the reaction mixture was concentrated *in vacuo* and purified by silica gel chromatography (elution gradient: 0-35% ethyl acetate in hexane) to yield 4-chloro-7-(3,4-dimethylphenyl)-5H-pyrrolo[2,3-d]pyrimidin-6-one (225 mg, 0.82 mmol, 24% yield) as a white solid. MS (ESI+) m/z calcd for $C_{14}H_{13}ClN_3O^+$ $[M + H]^+$ 274.1, found 274.1. UPLC analysis (method A), 2.48 min.

4-(3-chloro-4-methoxy-anilino)-7-(3,4-dimethylphenyl)-5H-pyrrolo[2,3-d]pyrimidin-6-one (**39**)

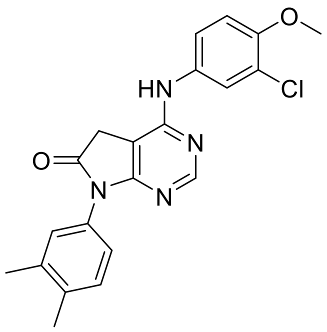

A microwave vial was charged with 4-chloro-7-(3,4-dimethylphenyl)-5H-pyrrolo[2,3-d]pyrimidin-6-one (90 mg, 0.33 mmol), 3-chloro-4-methoxyaniline (57 mg, 0.36 mmol), 4-methylbenzenesulfonic acid (5.7 mg, 0.033 mmol), NMP (1.0 mL) and diglyme (1.0 mL), sealed and heated at 180 °C for 2 h. Upon cooling to rt the reaction mixture was poured into H_2O, extracted with DCM (Caution! Carcinogenic; 3 x 20 mL), dried (Na_2SO_4), filtered, concentrated *in vacuo* and purified by preparatory HPLC (elution gradient: 30-70% MeCN in H_2O with 0.1% NH_3) to yield 4-(3-chloro-4-methoxy-anilino)-7-(3,4-dimethylphenyl)-5H-pyrrolo[2,3-d]pyrimidin-6-one **39** (51 mg, 0.13 mmol, 39% yield) as a white solid. MS (ESI+) m/z calcd for $C_{21}H_{20}ClN_4O_2^+$ $[M + H]^+$ 395.1, found 395.3. ^1H NMR (300 MHz, DMSO-d_6) δ 9.04 (s, 1H), 8.32 (s, 1H), 7.87 (d, *J* = 2.6 Hz, 1H), 7.54 (dd, *J* = 9.0, 2.6 Hz, 1H), 7.26 (d, *J* = 8.1 Hz, 1H), 7.19 – 7.09 (m, 3H), 3.84 (s, 3H), 3.67 – 3.60 (m, 2H), 2.27 (s, 3H), 2.26 (s, 3H). UPLC analysis (method D), 5.57 min, 100% purity.

Synthesis of N-(3-chloro-4-methoxyphenyl)-7-(3,4-dimethylphenyl)-5-methyl-7H-pyrrolo[2,3-d]pyrimidin-4-amine (40)

4-chloro-7-(3,4-dimethylphenyl)-5-methyl-pyrrolo[2,3-d]pyrimidine

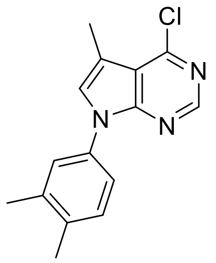

To a stirred solution of 4-chloro-5-methyl-1H-pyrrolo[2,3-d]pyrimidine (200 mg, 1.2 mmol), CuI (23 mg, 0.12 mmol), 4-iodo-*o*-xylene (305 mg, 0.19 mL, 1.3 mmol) and K_3PO_4 tribasic anhydrous (557 mg, 2.6 mmol) in 1,4-dioxane (Caution! Carcinogenic; 4.0 mL) was added *trans* N,N'-dimethylcyclohexane-1,2-diamine (34 mg, 0.038 mL, 0.24 mmol) and the reaction sealed under nitrogen and heated at 120 °C overnight. The reaction was filtered, concentrated *in vacuo* and purified by silica column chromatography (elution gradient: 0-40% ethyl acetate in hexane) to yield 4-chloro-7-(3,4-dimethylphenyl)-5-methyl-pyrrolo[2,3-d]pyrimidine (136 mg, 0.50 mmol, 42% yield). MS (ESI+) m/z calcd for $C_{15}H_{15}ClN_3^+$ $[M + H]^+$ 272.1, found 272.1. UPLC analysis (method A), 3.51 min.

N-(3-chloro-4-methoxyphenyl)-7-(3,4-dimethylphenyl)-5-methyl-7H-pyrrolo[2,3-d]pyrimidin-4-amine (**40**)

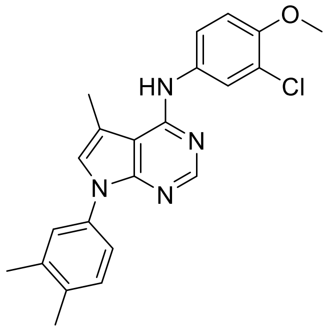

4-Chloro-7-(3,4-dimethylphenyl)-5-methyl-pyrrolo[2,3-d]pyrimidine (70 mg, 0.26 mmol), 3-chloro-4-methoxyaniline (49 mg, 0.31 mmol), DiPEA (100 mg, 0.13 mL, 0.77 mmol) and 4 N/1,4-dioxane (Caution! Carcinogenic; 0.065 mL, 0.26 mmol) in 1,4-dioxane (Caution! Carcinogenic; 3.0 mL) were sealed in a reaction vial and heated at 100 °C overnight. The reaction was opened and heated at 60 °C for 30 min to release the HCl before being basified with ammonia in methanol and concentrating *in vacuo*. The residue was further purified by

preparatory HPLC (elution gradient: 50-90% MeCN in H_2O with 0.1% NH_3) to yield N-(3-chloro-4-methoxyphenyl)-7-(3,4-dimethylphenyl)-5-methyl-7H-pyrrolo[2,3-d]pyrimidin-4-amine **40** (52 mg, 0.13 mmol, 51% yield). MS (ESI+) m/z calcd for $C_{22}H_{22}ClN_4O^+$ $[M + H]^+$ 393.1, found 393.3. ^1H NMR (300 MHz, DMSO-*d*$_6$) δ 8.26 (s, 1H), 8.15 (s, 1H), 7.85 (d, *J* = 2.6 Hz, 1H), 7.63 (dd, *J* = 8.9, 2.6 Hz, 1H), 7.57 (d, *J* = 2.0 Hz, 1H), 7.50 (dd, *J* = 8.1, 2.3 Hz, 1H), 7.43 (d, *J* = 1.1 Hz, 1H), 7.27 (d, *J* = 8.3 Hz, 1H), 7.15 (d, *J* = 9.0 Hz, 1H), 3.85 (s, 3H), 2.56 (d, *J* = 1.1 Hz, 3H), 2.29 (s, 3H), 2.27 (s, 3H). UPLC analysis (method C), 6.79 min, 100% purity.

Synthesis of N-(3-chloro-4-methoxyphenyl)-7-(2,4-dimethylphenyl)-7H-pyrrolo[2,3-d]pyrimidin-4-amine (41)

N-(3-chloro-4-methoxy-phenyl)-7H-pyrrolo[2,3-d]pyrimidin-4-amine

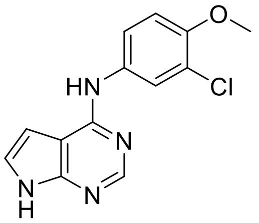

6-Chloro-7-deazapurine (500 mg, 3.3 mmol), 3-chloro-4-methoxyaniline (770 mg, 4.9 mmol) and DiPEA (631 mg, 0.84 mL, 4.9 mmol) were sealed in a reaction vial and the reaction heated at 120 °C overnight, followed by heating at 130 °C for a further 18 h. The reaction was concentrated *in vacuo* and purified by column chromatography (elution gradient: 0-20% MeOH in DCM; Caution! Carcinogenic) to yield N-(3-chloro-4-methoxy-phenyl)-7H-pyrrolo[2,3-d]pyrimidin-4-amine (267 mg, 0.97 mmol, 30% yield). MS (ESI+) m/z calcd for $C_{13}H_{12}ClN_4O^+$ $[M + H]^+$ 275.1, found 275.1. UPLC analysis (method A), 2.56 min.

N-(3-chloro-4-methoxyphenyl)-7-(2,4-dimethylphenyl)-7H-pyrrolo[2,3-d]pyrimidin-4-amine (**41**)

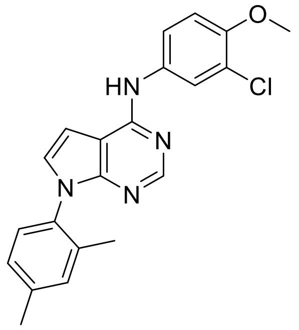

To a stirred solution of N-(3-chloro-4-methoxy-phenyl)-7H-pyrrolo[2,3-d]pyrimidin-4-amine (100 mg, 0.36 mmol), CuI (4.8 mg, 0.025 mmol), 4-iodo-*m*-xylene (106 mg, 0.065 mL, 0.46 mmol) and K_3PO_4 tribasic anhydrous (170 mg, 0.80 mmol) in 1,4-dioxane (Caution! Carcinogenic; 3.0 mL) was added *trans* N,N'-dimethylcyclohexane-1,2-diamine (7.8 mg, 8.6 μL, 0.055 mmol) and the reaction was sealed under nitrogen and heated at 130 °C for 72 h. Further *trans* N,N'-dimethylcyclohexane-1,2-diamine (52 mg, 57 μl, 0.37 mmol) and CuI (35 mg, 0.18 mmol) were added and the reaction stirred at 150 °C for 72 h. The reaction mixture was applied to an SCX-2 cartridge, washed with DCM (Caution! Carcinogenic) and MeOH and eluted with 2 M methanolic ammonia. The basic fraction was concentrated *in vacuo*, then purified by preparatory HPLC (elution gradient: 40-80% MeCN in H_2O with 0.1% NH_3) to yield N-(3-chloro-4-methoxyphenyl)-7-(2,4-dimethylphenyl)-7H-pyrrolo[2,3-d]pyrimidin-4-amine **41** (34 mg, 0.089 mmol, 24% yield). MS (ESI+) m/z calcd for $C_{21}H_{20}ClN_4O^+$ $[M + H]^+$ 379.1, found 379.3. ^1H NMR (300 MHz, Chloroform-*d*) δ 8.42 (s, 1H), 7.66 (d, *J* = 2.6 Hz, 1H), 7.47 (dd, *J* = 8.8, 2.6 Hz, 1H), 7.31 (s, 1H), 7.21 – 7.07 (m, 3H), 7.02 – 6.93 (m, 2H), 6.17 (d, *J* = 3.6 Hz, 1H), 3.94 (s, 3H), 2.38 (s, 3H), 2.04 (s, 3H). UPLC analysis (method C), 6.06 min, 98% purity.

Synthesis of N-(3-chloro-4-methoxyphenyl)-7-(4-methoxyphenyl)-7H-pyrrolo[2,3-d]pyrimidin-4-amine (42)

4-chloro-7-(4-methoxyphenyl)pyrrolo[2,3-d]pyrimidine

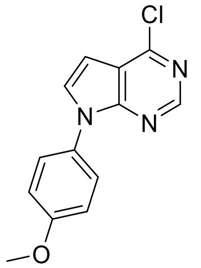

To a stirred solution of 6-chloro-7-deazapurine (500 mg, 3.3 mmol), CuI (43 mg, 0.23 mmol), 4-iodophenyl methyl ether (952 mg, 0.58 mL, 4.1 mmol) and K_3PO_4 tribasic anhydrous (1520 mg, 7.2 mmol) in 1,4-dioxane (Caution! Carcinogenic; 10 mL) was added *trans* N,N'-dimethylcyclohexane-1,2-diamine (70 mg, 0.077 mL, 0.49 mmol) and the reaction was sealed under nitrogen and heated at 120 ^{o}C overnight. The reaction was filtered and concentrated *in vacuo*, then purified by silica column chromatography (elution gradient: 0-50% ethyl acetate in hexane) to yield 4-chloro-7-(4-methoxyphenyl)pyrrolo[2,3-d]pyrimidine (640 mg, 2.5 mmol, 76% yield). MS (ESI+) m/z calcd for $C_{13}H_{11}ClN_4O^+$ $[M + H]^+$ 260.1, found 260.1. UPLC analysis (method A), 3.04 min.

N-(3-chloro-4-methoxyphenyl)-7-(4-methoxyphenyl)-7H-pyrrolo[2,3-d]pyrimidin-4-amine (**42**)

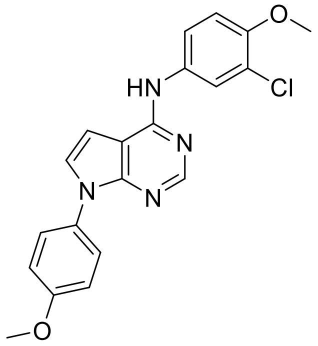

A mixture of 4-chloro-7-(4-methoxyphenyl)pyrrolo[2,3-d]pyrimidine (100 mg, 0.39 mmol), 3-chloro-4-methoxyaniline (67 mg, 0.42 mmol), Cs_2CO_3 (274 mg, 0.85 mmol), $Pd_2(dba)_3$ (18 mg, 0.019 mmol) and Xantphos (22 mg, 0.039 mmol) in 1,4-dioxane (Caution! Carcinogenic; 10 mL) was sealed in a microwave tube and degassed with nitrogen for 5 min. The reaction was heated in a microwave at 125 ^{o}C for 30 min. The reaction

mixture was filtered through a small pad of silica, concentrated *in vacuo* and purified by preparatory HPLC (elution gradient: 50-90% MeCN in H_2O with 0.1% NH_3) to yield N-(3-chloro-4-methoxyphenyl)-7-(4-methoxyphenyl)-7H-pyrrolo[2,3-d]pyrimidin-4-amine **42** (39 mg, 0.10 mmol, 27% yield). MS (ESI+) m/z calcd for $C_{20}H_{18}ClN_4O_2^+$ [M + H]$^+$ 381.1, found 381.2. ^{1}H NMR (300 MHz, DMSO-d_6) δ 9.47 (s, 1H), 8.34 (s, 1H), 8.11 (d, *J* = 2.6 Hz, 1H), 7.78 – 7.65 (m, 3H), 7.63 – 7.58 (m, 1H), 7.13 (dd, *J* = 21.4, 9.0 Hz, 3H), 6.96 (d, *J* = 3.7 Hz, 1H), 3.89 – 3.76 (m, 6H). UPLC analysis (method C), 5.61 min, 99% purity.

Synthesis of 43, 44, 45 and 46 from 4-chloro-7-(3,4-dimethylphenyl)pyrrolo[2,3-d]pyrimidine

4-chloro-7-(3,4-dimethylphenyl)pyrrolo[2,3-d]pyrimidine

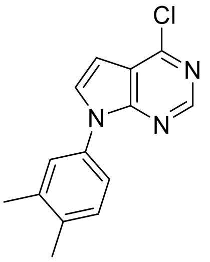

To a stirred solution of 6-chloro-7-deazapurine (1.0 g, 6.5 mmol), CuI (87 mg, 0.46 mmol), 4-iodo-*o*-xylene (1.9 g, 1.2 mL, 8.1 mmol) and K_3PO_4 tribasic anhydrous (3.0 mg, 14 mmol) in 1,4-dioxane (Caution! Carcinogenic; 10 mL) was added *trans* N,N'-dimethylcyclohexane-1,2-diamine (139 mg, 0.15 mL, 0.98 mmol) and the reaction was sealed under a nitrogen atmosphere and heated at 110 ºC for 48 h. The reaction mixture was filtered, concentrated *in vacuo* and purified by silica column chromatography (elution gradient: 0-40% ethyl acetate in hexane) to yield 4-chloro-7-(3,4-dimethylphenyl)pyrrolo[2,3-d]pyrimidine (1.1 g, 4.4 mmol, 67% yield). MS (ESI+) m/z calcd for $C_{14}H_{13}ClN_3^+$ [M + H]$^+$ 258.1, found 258.1. UPLC analysis (method A), 3.36 min,

N-(3-((7-(3,4-dimethylphenyl)-7H-pyrrolo[2,3-d]pyrimidin-4-yl)amino)phenyl)acetamide (**43**)

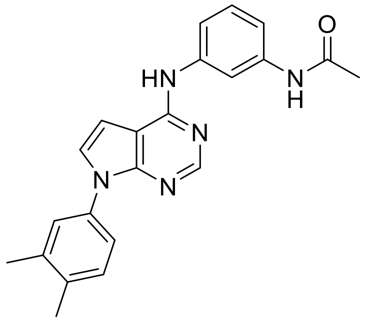

A microwave vial was charged with N-(3-aminophenyl)acetamide (61 mg, 0.41 mmol), 4-chloro-7-(3,4-dimethylphenyl)pyrrolo[2,3-d]pyrimidine (100 mg, 0.39 mmol) and DCE (Caution! Possible arcinogen; 3.0 mL) and the vial was sealed under a nitrogen atmosphere. The reaction was heated at 130 °C for 3 h, then at 135 °C for a further 3 h. The reaction was concentrated *in vacuo* and purified by preparatory HPLC (elution gradient: 30-70% MeCN in H_2O with 0.1% NH_3) to yield N-(3-((7-(3,4-dimethylphenyl)-7H-pyrrolo[2,3-d]pyrimidin-4-yl)amino)phenyl)acetamide **43** (54 mg, 0.14 mmol, 37% yield). MS (ESI+) m/z calcd for $C_{22}H_{22}N_5O^+$ $[M + H]^+$ 372.2, found 372.3. ^{1}H NMR (300 MHz, DMSO-d_6) δ 9.96 (s, 1H), 9.50 (s, 1H), 8.36 (s, 1H), 8.16 (d, J = 2.0 Hz, 1H), 7.65 (d, J = 3.7 Hz, 1H), 7.63-7.58 (m, 2H), 7.53 (dd, J = 8.1, 2.3 Hz, 1H), 7.29 (d, J = 8.2 Hz, 1H), 7.28 – 7.22 (m, 2H), 7.06 (d, J = 3.7 Hz, 1H), 2.31 (s, 3H), 2.28 (s, 3H), 2.06 (s, 3H). UPLC analysis (method C), 5.13 min, 100% purity.

Synthesis of N-(4-((7-(3,4-dimethylphenyl)-7H-pyrrolo[2,3-d]pyrimidin-4-yl)amino)phenyl)acetamide (44)

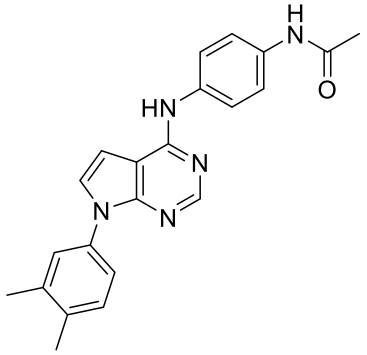

A stirred mixture of 4-chloro-7-(3,4-dimethylphenyl)pyrrolo[2,3-d]pyrimidine (30 mg, 0.12 mmol), 4'-aminoacetanilide (22 mg, 0.15 mmol), Cs_2CO_3 (83 mg, 0.26 mmol), $Pd_2(dba)_3$ (5.3 mg, 0.0058 mmol), and Xantphos (6.7 mg, 0.012 mmol) in 1,4-dioxane (Caution! Carcinogenic; 3.0 mL) was sealed in a microwave tube

and degassed with nitrogen for 5 min. The reaction was heated in a microwave at 125 °C for 30 min. The reaction was filtered through a small pad of silica, concentrated *in vacuo* and purified by preparatory HPLC (elution gradient: 40-80% MeCN in H_2O with 0.1% NH_3) to yield N-(4-((7-(3,4-dimethylphenyl)-7H-pyrrolo[2,3-d]pyrimidin-4-yl)amino)phenyl)acetamide **44** (15 mg, 0.041 mmol, 35% yield). MS (ESI+) m/z calcd for $C_{22}H_{22}N_5O^+$ [M + H]$^+$ 372.2, found 372.3. ^{1}H NMR (300 MHz, DMSO-*d*$_6$) δ 9.91 (s, 1H), 9.43 (s, 1H), 8.32 (s, 1H), 7.76 (d, *J* = 9.0 Hz, 2H), 7.66 – 7.47 (m, 5H), 7.29 (d, *J* = 8.2 Hz, 1H), 6.96 (d, *J* = 3.7 Hz, 1H), 2.31 (s, 3H), 2.28 (s, 3H), 2.04 (s, 3H). UPLC analysis (method C), 4.97 min, 97% purity.

Synthesis of 7-(3,4-dimethylphenyl)-N-(6-methoxypyridin-3-yl)-7H-pyrrolo[2,3-d]pyrimidin-4-amine (45)

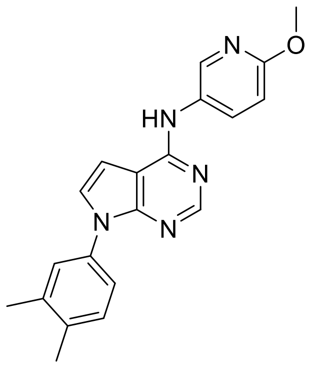

A stirred mixture of 4-chloro-7-(3,4-dimethylphenyl)pyrrolo[2,3-d]pyrimidine (37 mg, 0.14 mmol), 6-methoxypyridin-3-amine (36 mg, 0.29 mmol), K_3PO_4 (61 mg, 0.29 mmol) and XPhos Pd G2 (11 mg, 0.014 mmol) in toluene (3.0 mL) was sealed in a microwave tube and degassed with nitrogen for 5 min. The reaction was stirred at 150 °C for 36 h. The reaction was applied to an SCX-2 cartridge, washed with DCM (Caution! Carcinogenic) and MeOH and eluted with 2 M methanolic ammonia. The basic fraction was concentrated *in vacuo* and purified by preparatory HPLC (elution gradient: 40-80% MeCN in H_2O with 0.1% NH_3) to yield 7-(3,4-dimethylphenyl)-N-(6-methoxypyridin-3-yl)-7H-pyrrolo[2,3-d]pyrimidin-4-amine **45** (7.0 mg, 0.020 mmol, 14% yield). MS (ESI+) m/z calcd for $C_{20}H_{20}N_5O^+$ [M + H]$^+$ 346.2, found 346.3. ^{1}H NMR (300 MHz, Chloroform-*d*) δ 8.46 (s, 1H), 8.32 (dd, *J* = 2.8, 0.7 Hz, 1H), 7.94 (dd, *J* = 8.8, 2.8 Hz, 1H), 7.42 (d, *J* = 2.2 Hz, 1H), 7.36 (dd, *J* = 8.0, 2.3 Hz, 1H), 7.26 (s, 1H), 7.21 (d, *J* = 3.6 Hz, 1H), 7.00 (s, 1H), 6.84 (dd, *J* = 8.8, 0.7 Hz, 1H), 6.21 (d, *J* = 3.7 Hz, 1H), 3.99 (s, 3H), 2.35 (s, 3H), 2.33 (s, 3H). UPLC analysis (method C), 5.56 min, 96% purity.

Synthesis of N-(7-(3,4-dimethylphenyl)-7H-pyrrolo[2,3-d]pyrimidin-4-yl)isoxazol-4-amine (46)

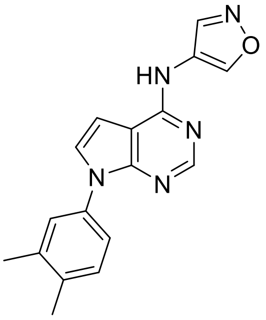

A stirred mixture of 4-chloro-7-(3,4-dimethylphenyl)pyrrolo[2,3-d]pyrimidine (100 mg, 0.39 mmol), 1,2-oxazol-4-amine hydrochloride (58 mg, 0.49 mmol), Cs_2CO_3 (400 mg, 1.2 mmol), $Pd_2(dba)_3$ (18 mg, 0.019 mmol) and Xantphos (22 mg, 0.039 mmol) in 1,4-dioxane (Caution! Carcinogenic; 3.0 mL) was sealed in a microwave tube and degassed with nitrogen for 5 min. The reaction was heated in a microwave at 120 °C for 30 min. The reaction mixture was passed through a pad of silica, concentrated *in vacuo* and purified by preparatory HPLC (elution gradient: 40-80% MeCN in H_2O with 0.1% NH_3) to yield N-(7-(3,4-dimethylphenyl)-7H-pyrrolo[2,3-d]pyrimidin-4-yl)isoxazol-4-amine **46** (16 mg, 0.052 mmol, 13% yield). MS (ESI+) m/z calcd for $C_{17}H_{16}N_5O^+$ [M + H]$^+$ 306.1, found 306.2. ^{1}H NMR (300 MHz, DMSO-d_6) δ 9.94 (s, 1H), 9.36 (s, 1H), 8.80 (s, 1H), 8.43 (s, 1H), 7.67 (d, J = 3.6 Hz, 1H), 7.59 (d, J = 2.0 Hz, 1H), 7.52 (dd, J = 8.1, 2.3 Hz, 1H), 7.30 (d, J = 8.1 Hz, 1H), 6.89 (d, J = 3.7 Hz, 1H), 3.23 (s, 3H), 2.29 (s, 3H). UPLC analysis (method D), 5.38 min, 96% purity.

Synthesis of ARUK2004789 7-(3-aminophenyl)-N-(4-methoxyphenyl)-7H-pyrrolo[2,3-d]pyrimidin-4-amine (47)

4-chloro-7-(3-nitrophenyl)pyrrolo[2,3-d]pyrimidine

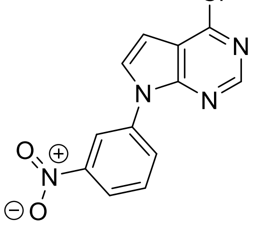

To a stirred solution of 6-chloro-7-deazapurine (500 mg, 3.3 mmol), CuI (43 mg, 0.23 mmol), 1-iodo-3-nitrobenzene (970 mg, 0.60 mL, 3.9 mmol) and K_3PO_4 tribasic anhydrous (1.5 g, 7.2 mmol) in 1,4-dioxane (Caution! Carcinogenic; 10 mL) was added *trans* N,N'-dimethylcyclohexane-1,2-diamine (46 mg, 0.051 mL, 0.33

mmol) and the reaction sealed under nitrogen and heated at 110 °C overnight. The reaction was filtered, concentrated *in vacuo* and purified by silica column chromatography (elution gradient: 0-100% ethyl acetate in hexane) to yield 4-chloro-7-(3-nitrophenyl)pyrrolo[2,3-d]pyrimidine (60 mg, 0.22 mmol, 6.7% yield). MS (ESI+) m/z calcd for $C_{12}H_8N_4O_2^+$ $[M + H]^+$ 275.0, found 275.1. UPLC analysis (method A), 3.40 min.

N-(3-chloro-4-methoxy-phenyl)-7-(3-nitrophenyl)pyrrolo[2,3-d]pyrimidin-4-amine

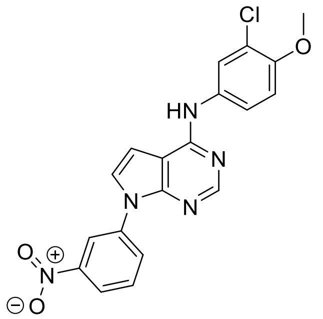

A stirred mixture of 4-chloro-7-(3-nitrophenyl)pyrrolo[2,3-d]pyrimidine (50 mg, 0.18 mmol), Cs_2CO_3 (130 mg, 0.40 mmol), $Pd_2(dba)_3$ (8.0 mg, 0.0087 mmol), Xantphos (11 mg, 0.019 mmol) and 3-chloro-4-methoxyaniline (36 mg, 0.23 mmol) in 1,4-dioxane (Caution! Carcinogenic; 3.0 mL) was sealed in a microwave tube and degassed with nitrogen for 5 min. The reaction was heated in a microwave at 125 °C for 55 min. The reaction was placed on an SCX-2 cartridge, washed with DCM (Caution! Carcinogenic) and MeOH and eluted with 2 M methanolic ammonia. The basic fraction was concentrated *in vacuo* and purified by preparatory HPLC (elution gradient: 40-80% MeCN in H_2O with 0.1% NH_3) to yield N-(3-chloro-4-methoxy-phenyl)-7-(3-nitrophenyl)pyrrolo[2,3-d]pyrimidin-4-amine (20 mg, 0.049 mmol, 27% yield). MS (ESI+) m/z calcd for $C_{19}H_{15}ClN_5O_3^+$ $[M + H]^+$ 396.1, found 396.2. ^1H NMR (300 MHz, DMSO-d_6) δ 9.60 (s, 1H), 8.94 (t, J = 2.2 Hz, 1H), 8.46 (s, 1H), 8.38 (ddd, J = 8.2, 2.2, 0.9 Hz, 1H), 8.22 (ddd, J = 8.3, 2.3, 0.9 Hz, 1H), 8.10 (d, J = 2.7 Hz, 1H), 7.95 (d, J = 3.8 Hz, 1H), 7.86 (t, J = 8.2 Hz, 1H), 7.72 (dd, J = 9.0, 2.6 Hz, 1H), 7.19 (d, J = 9.0 Hz, 1H), 7.08 (d, J = 3.8 Hz, 1H), 3.86 (s, 3H). UPLC analysis (method D), 5.55 min, 100% purity.

7-(3-aminophenyl)-N-(4-methoxyphenyl)-7H-pyrrolo[2,3-d]pyrimidin-4-amine (**47**)

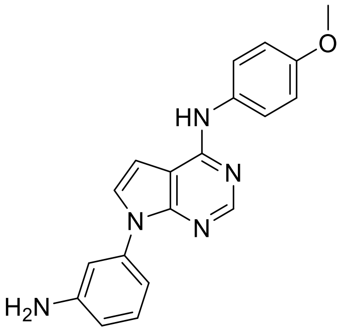

A stirred solution of N-(3-chloro-4-methoxy-phenyl)-7-(3-nitrophenyl)pyrrolo[2,3-d]pyrimidin-4-amine (15 mg, 0.038 mmol) in MeOH (10 mL) was evacuated *in vacuo* and back filled with nitrogen 3 times before 10% Pd/C (0.040 mmol) was added and the reaction again placed under nitrogen. The reaction was evacuated *in vacuo* and back filled with hydrogen gas 3 times and left under an atmosphere of hydrogen with stirring overnight. The reaction was filtered through Celite® and concentrated *in vacuo*. Purification by preparatory HPLC (elution gradient: 20-60% MeCN in H_2O with 0.1% NH_3) yielded 7-(3-aminophenyl)-N-(4-methoxyphenyl)-7H-pyrrolo[2,3-d]pyrimidin-4-amine **47** (2.2 mg, 0.0067 mmol, 18% yield). MS (ESI+) m/z calcd for $C_{19}H_{18}N_5O^+$ [M + H]$^+$ 332.2, found 332.2. ^{1}H NMR (300 MHz, DMSO-*d*$_6$) δ 9.33 (s, 1H), 8.27 (s, 1H), 7.75 – 7.68 (m, 2H), 7.52 (d, *J* = 3.7 Hz, 1H), 7.14 (t, *J* = 8.0 Hz, 1H), 7.02 (t, *J* = 2.1 Hz, 1H), 6.98 – 6.91 (m, 2H), 6.89 (d, *J* = 3.6 Hz, 1H), 6.82 (ddd, *J* = 7.9, 2.0, 0.8 Hz, 1H), 6.55 (ddd, *J* = 8.1, 2.1, 0.8 Hz, 1H), 5.36 (s, 2H), 3.76 (s, 3H). UPLC analysis (method C), 4.38 min, 100% purity.

Synthesis of N-(3-chloro-4-methoxyphenyl)-7-cyclopropyl-7H-pyrrolo[2,3-d]pyrimidin-4-amine (48)

4-Chloro-7-cyclopropyl-pyrrolo[2,3-d]pyrimidine

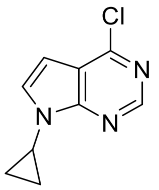

A solution of 6-chloro-7-deazapurine (1.0 g, 6.5 mmol), cyclopropyl boronic acid (1.12 mg, 13 mmol), 2,2'-bipyridine (1.0 g, 1.0 mL, 6.5 mmol), $Cu(OAc)_2$ (1.18 g, 6.5 mmol) and Na_2CO_3 (1.38 g, 13 mmol) in DCE (Caution! Possible arcinogen; 35 mL) was stirred at reflux for 42 h. The reaction was cooled to rt, filtered,

concentrated *in vacuo* and purified by silica column chromatography (elution gradient: 0-50% ethyl acetate in hexane) to give 4-chloro-7-cyclopropyl-pyrrolo[2,3-d]pyrimidine (603 mg, 3.1 mmol, 48% yield). MS (ESI+) m/z calcd for $C_9H_8ClN_3^+$ $[M + H]^+$ 194.1, found 194.1. UPLC analysis (method A), 2.63 min.

N-(3-chloro-4-methoxyphenyl)-7-cyclopropyl-7H-pyrrolo[2,3-d]pyrimidin-4-amine (**48**)

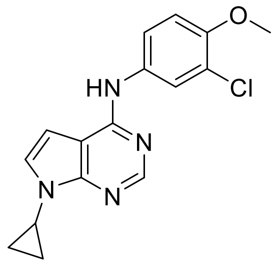

A stirred mixture of 4-chloro-7-cyclopropyl-pyrrolo[2,3-d]pyrimidine (100 mg, 0.52 mmol), 3-chloro-4-methoxyaniline (102 mg, 0.65 mmol), Cs_2CO_3 (367 mg, 1.1 mmol), $Pd_2(dba)_3$ (247, 0.026 mmol) and Xantphos (30 mg, 0.052 mmol) in 1,4-dioxane (Caution! Carcinogenic; 3.0 mL) was sealed in a microwave vial and degassed with nitrogen for 5 min. The reaction was heated in a microwave at 125 ºC for 60 min. The reaction was placed on an SCX-2 cartridge, washed with DCM (Caution! Carcinogenic) and MeOH and eluted with 2 M methanolic ammonia. The basic fraction was concentrated *in vacuo* and purified by preparatory HPLC (elution gradient: 20-60% MeCN in H_2O with 0.1% NH_3) to yield N-(3-chloro-4-methoxyphenyl)-7-cyclopropyl-7H-pyrrolo[2,3-d]pyrimidin-4-amine **48** (28 mg, 0.088 mmol, 17% yield). MS (ESI+) m/z calcd for $C_{16}H_{16}ClN_4O^+$ $[M + H]^+$ 315.1, found 315.2. ^1H NMR (300 MHz, DMSO-d_6) δ 9.31 (s, 1H), 8.31 (s, 1H), 8.09 (d, *J* = 2.6 Hz, 1H), 7.69 (dd, *J* = 9.0, 2.6 Hz, 1H), 7.23 (d, *J* = 3.6 Hz, 1H), 7.14 (d, *J* = 9.1 Hz, 1H), 6.70 (d, *J* = 3.6 Hz, 1H), 3.83 (s, 3H), 3.55 (tt, *J* = 6.5, 4.6 Hz, 1H), 1.01 (ddt, *J* = 6.0, 4.3, 2.6 Hz, 4H). UPLC analysis (method C), 4.86 min, 96% purity.

Synthesis of 7-((1H-imidazol-4-yl)methyl)-N-(3-chloro-4-methoxyphenyl)-7H-pyrrolo[2,3-d]pyrimidin-4-amine (49)

4-chloro-7-(1H-imidazol-4-ylmethyl)pyrrolo[2,3-d]pyrimidine

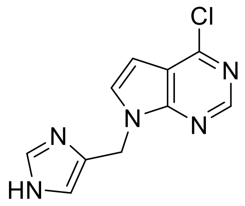

To a stirred solution of 6-chloro-7-deazapurine (250 mg, 1.6 mmol) in DMF (Caution! Carcinogenic; 5.0 mL) at 0 ºC was added NaH 60% w/w (Caution! Flammable, corrosive; 157 mg, 3.9 mmol) and the reaction was stirred for 10 min before 4-(chloromethyl)-1H-imidazole hydrochloride (Caution! toxic; 311 mg, 2.0 mmol) was added and the reaction allowed to warm to rt slowly and stirred for a further 1 h. The reaction was diluted with H_2O and DCM (Caution! Carcinogenic), extracted with 3:1 $CHCl_3$:IPA, dried ($MgSO_4$) and concentrated *in vacuo* to give 4-chloro-7-(1H-imidazol-4-ylmethyl)pyrrolo[2,3-d]pyrimidine (327 mg, 1.4 mmol, 86% yield) which was used without further purification. MS (ESI+) m/z calcd for $C_{10}H_9ClN_5^+$ $[M + H]^+$ 234.1, found 234.2. UPLC analysis (method B), 1.93 min.

7-((1H-imidazol-4-yl)methyl)-N-(3-chloro-4-methoxyphenyl)-7H-pyrrolo[2,3-d]pyrimidin-4-amine (**49**)

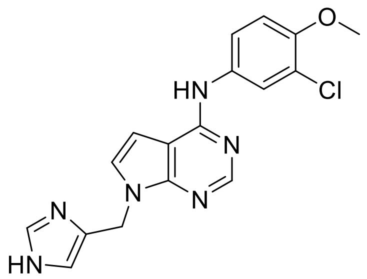

A stirred mixture of 3-chloro-4-methoxyaniline (662 mg, 4.2 mmol), 4-chloro-7-(1H-imidazol-4-ylmethyl)pyrrolo[2,3-d]pyrimidine (327 mg, 1.4 mmol) and DCE (Caution! Possible arcinogen; 10 mL) was sealed in a microwave vial under a nitrogen atmosphere. The reaction was heated to 130 ºC for 4 h. The reaction mixture was diluted with H_2O and DCM (Caution! Carcinogenic), and the aqueous phase was concentrated *in vacuo* and purified by preparatory HPLC (elution gradient: 10-50% MeCN in H_2O with 0.1% NH_3) to yield 7-((1H-imidazol-4-yl)methyl)-N-(3-chloro-4-methoxyphenyl)-7H-pyrrolo[2,3-d]pyrimidin-4-amine **49** (74 mg,

0.21 mmol, 15% yield). MS (ESI+) m/z calcd for $C_{17}H_{16}ClN_6O^+$ [M + H]$^+$ 355.1, found 355.2. ^{1}H NMR (300 MHz, DMSO-d_6) δ 11.97 (s, 1H), 9.32 (s, 1H), 8.32 (s, 1H), 8.10 (d, *J* = 2.6 Hz, 1H), 7.70 (dd, *J* = 9.0, 2.6 Hz, 1H), 7.58 (d, *J* = 1.0 Hz, 1H), 7.28 (d, *J* = 3.5 Hz, 1H), 7.14 (d, *J* = 9.1 Hz, 1H), 7.01 (s, 1H), 6.73 (d, *J* = 3.5 Hz, 1H), 5.24 (s, 2H), 3.84 (s, 3H). UPLC analysis (method D), 2.91 min, 100% purity.

Synthesis of N-(3-chloro-4-methoxyphenyl)-7-(1-methyl-1H-pyrazol-3-yl)-7H-pyrrolo[2,3-d]pyrimidin-4-amine (50)

4-chloro-7-(1-methylpyrazol-3-yl)pyrrolo[2,3-d]pyrimidine

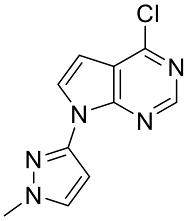

A solution of 6-chloro-7-deazapurine (200 mg, 1.3 mmol), 1-methyl-3-(4,4,5,5-tetramethyl-1,3,2-dioxaborolan-2-yl)-1H-pyrazole (542 mg, 2.6 mmol), 2,2'-bipyridine (203 mg, 0.21 mL, 1.3 mmol), $Cu(OAc)_2$ (237 mg, 1.3 mmol) and Na_2CO_3 (276 mg, 2.6 mmol) in DCE (Caution! Possible arcinogen; 10 mL) was stirred in a microwave vial at 110 ºC for 24 h. The reaction mixture was cooled to rt, washed with Cu_2SO_4 solution, extracted with DCM (Caution! Carcinogenic), dried ($MgSO_4$) and concentrated *in vacuo*. The material was purified by silica column chromatography (elution gradient: 0-100% ethyl acetate in hexane) to yield 4-chloro-7-(1-methylpyrazol-3-yl)pyrrolo[2,3-d]pyrimidine (142 mg, 0.61 mmol, 47% yield). MS (ESI+) m/z calcd for $C_{10}H_9ClN_5^+$ [M + H]$^+$ 234.1, found 234.1. UPLC analysis (method A), 2.90 min.

N-(3-chloro-4-methoxyphenyl)-7-(1-methyl-1H-pyrazol-3-yl)-7H-pyrrolo[2,3-d]pyrimidin-4-amine (**50**)

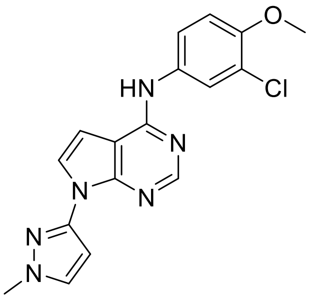

A stirred mixture of 4-chloro-7-(1-methylpyrazol-3-yl)pyrrolo[2,3-d]pyrimidine (50 mg, 0.22 mmol), 3-chloro-4-methoxyaniline (42 mg, 0.27 mmol), Cs_2CO_3 (153 mg, 0.47 mmol), $Pd_2(dba)_3$ (10 mg, 0.011 mmol) and Xantphos (12 mg, 0.021 mmol) in 1,4-dioxane (Caution! Carcinogenic; 3.0 mL) was sealed in a microwave tube and degassed with nitrogen for 5 min. The reaction was heated in a microwave at 125 °C for 45 min. The reaction was applied to an SCX-2 cartridge, washed with DCM (Caution! Carcinogenic) and MeOH and eluted with 2 M methanolic ammonia. The basic fraction was concentrated *in vacuo* and purified by preparatory HPLC (elution gradient: 40-80% MeCN in H_2O with 0.1% NH_3) to yield N-(3-chloro-4-methoxyphenyl)-7-(1-methyl-1H-pyrazol-3-yl)-7H-pyrrolo[2,3-d]pyrimidin-4-amine **50** (19 mg, 0.054 mmol, 25% yield). MS (ESI+) m/z calcd for $C_{17}H_{16}ClN_6O^+$ $[M + H]^+$ 355.1, found 355.2. ^1H NMR (300 MHz, DMSO-d_6) δ 9.49 (s, 1H), 8.40 (s, 1H), 8.09 (d, J = 2.6 Hz, 1H), 7.81 (d, J = 2.1 Hz, 1H), 7.74 – 7.66 (m, 2H), 7.17 (d, J = 9.1 Hz, 1H), 6.95 (d, J = 3.7 Hz, 1H), 6.92 (d, J = 2.3 Hz, 1H), 3.88 (s, 3H), 3.85 (s, 3H). UPLC analysis (method C), 5.49 min, 100% purity.

Data for the commercially available compound 20

1-(4-chloro-2-methylphenyl)-N-(3-chloro-4-methoxyphenyl)-1H-pyrazolo[3,4-d]pyrimidin-4-amine **20**

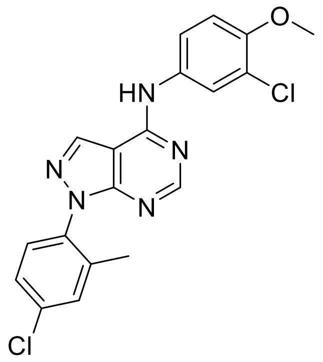

MS (ESI+) m/z calcd for $C_{19}H_{16}Cl_2N_5O^+$ $[M + H]^+$ 400.1, found 400.1. ^1H NMR (300 MHz, DMSO-d_6) δ 10.23 (s, 1H), 8.48 (s, 1H), 8.41 (s, 1H), 8.06 (s, 1H), 7.67 (dd, J = 8.9, 2.5 Hz, 1H), 7.58 (d, J = 1.1 Hz, 1H), 7.45 (d, J = 1.9 Hz, 2H), 7.21 (d, J = 9.1 Hz, 1H), 3.87 (s, 3H), 2.09 (s, 3H). ^{13}C NMR (75 MHz, DMSO) δ 156.13, 154.43, 153.85, 150.98, 137.41, 135.55, 133.52, 133.33, 132.50, 130.69, 129.46, 126.57, 123.17, 121.40, 120.59, 112.94, 101.06, 56.26, 17.63. UPLC analysis (method C), 5.88 min, 100% purity.

Spectra for key compounds 20, 36, 39, 43, and 45

1-(4-chloro-2-methylphenyl)-N-(3-chloro-4-methoxyphenyl)-1H-pyrazolo[3,4-d]pyrimidin-4-amine (**20**)

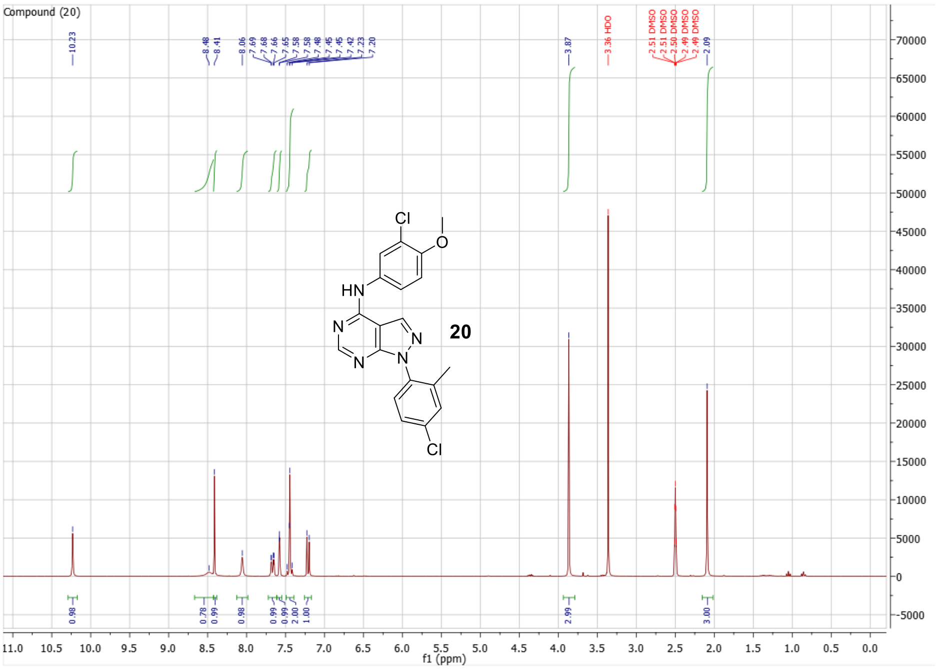

^{1}H NMR (300 MHz, DMSO-d_6) δ 10.23 (s, 1H), 8.48 (s, 1H), 8.41 (s, 1H), 8.06 (s, 1H), 7.67 (dd, J = 8.9, 2.5 Hz, 1H), 7.58 (d, J = 1.1 Hz, 1H), 7.45 (d, J = 1.9 Hz, 2H), 7.21 (d, J = 9.1 Hz, 1H), 3.87 (s, 3H), 2.09 (s, 3H).

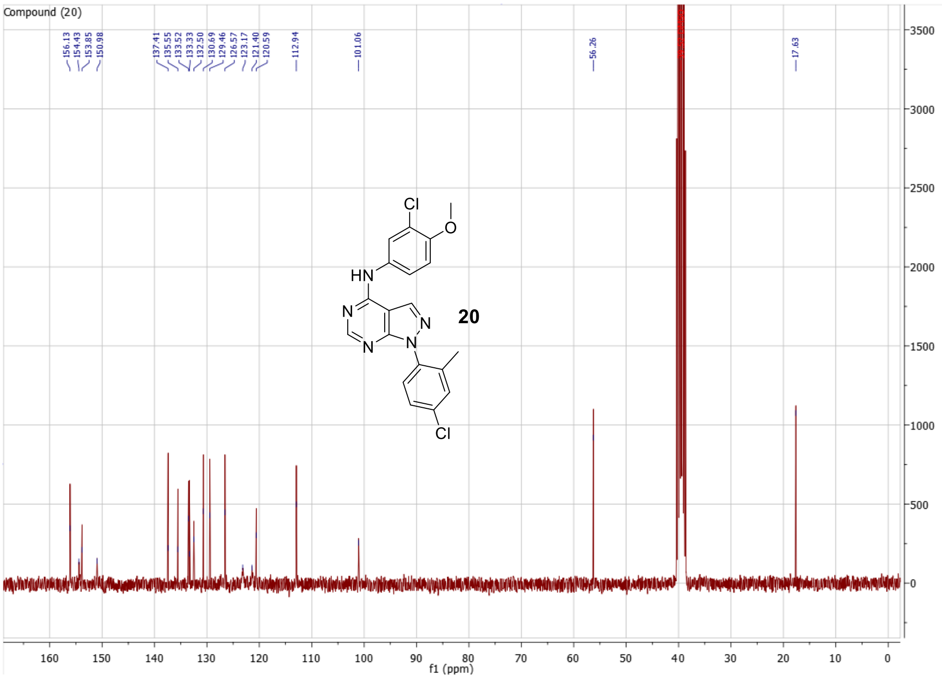

^{13}C NMR (75 MHz, DMSO) δ 156.13, 154.43, 153.85, 150.98, 137.41, 135.55, 133.52, 133.33, 132.50, 130.69, 129.46, 126.57, 123.17, 121.40, 120.59, 112.94, 101.06, 56.26, 17.63.

N-(3-chloro-4-methoxy-phenyl)-7-(3,4-dimethylphenyl)pyrrolo[2,3-d]pyrimidin-4-amine (**36**)

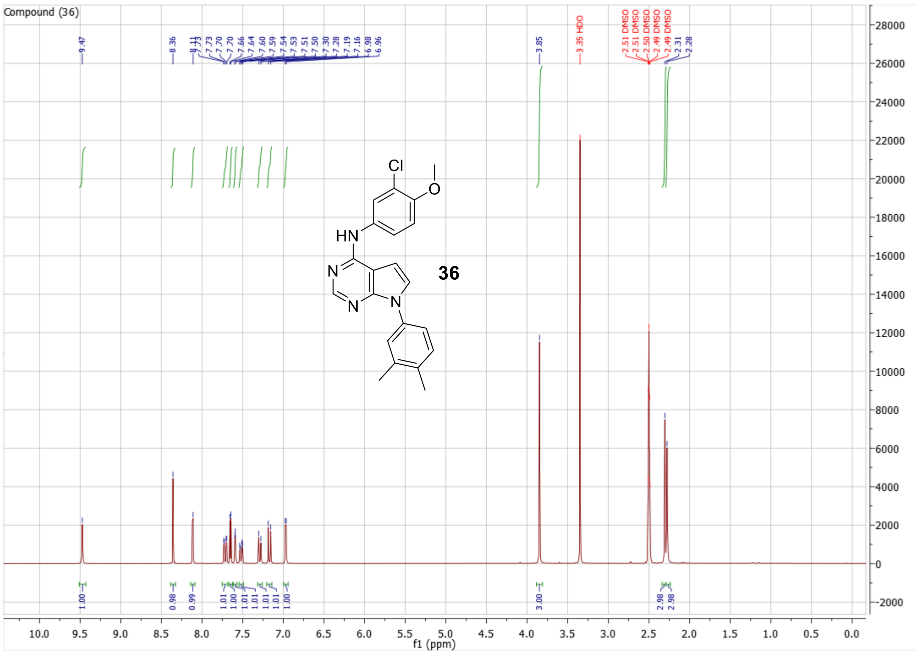

^{1}H NMR (300 MHz, DMSO-d_6) δ 9.47 (s, 1H), 8.36 (s, 1H), 8.11 (d, J = 2.6 Hz, 1H), 7.72 (dd, J = 9.0, 2.6 Hz, 1H), 7.65 (d, J = 3.7 Hz, 1H), 7.59 (d, J = 2.0 Hz, 1H), 7.52 (dd, J = 8.2, 2.3 Hz, 1H), 7.29 (d, J = 8.3 Hz, 1H), 7.17 (d, J = 9.1 Hz, 1H), 6.97 (d, J = 3.7 Hz, 1H), 3.85 (s, 3H), 2.31 (s, 3H), 2.28 (s, 3H).

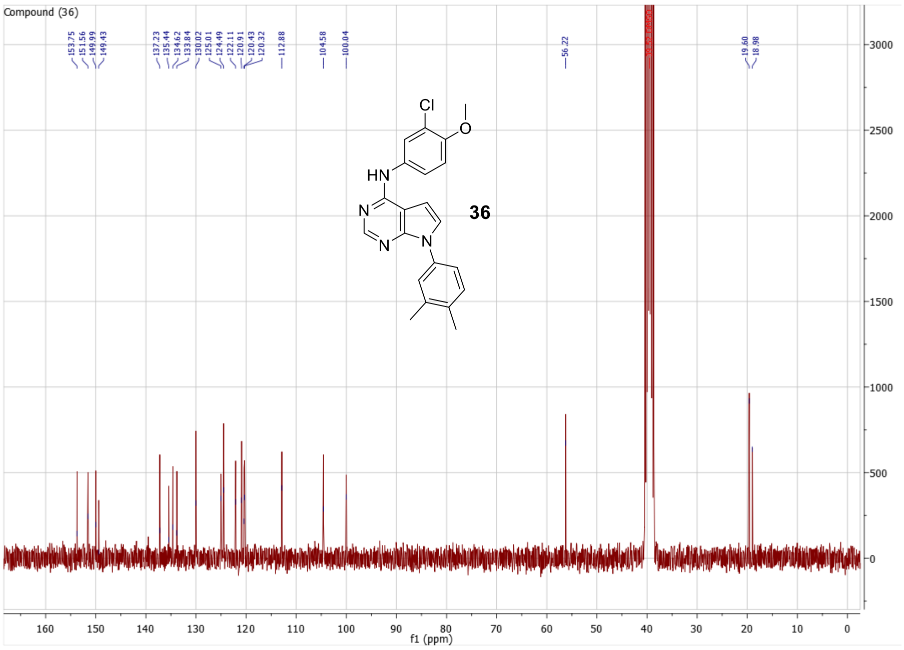

^{13}C NMR (75 MHz, DMSO) δ 153.75, 151.56, 149.99, 149.43, 137.23, 135.44, 134.62, 133.84, 130.02, 125.01, 124.49, 122.11, 120.91, 120.43, 120.32, 112.88, 104.58, 100.04, 56.22, 19.60, 18.98.

4-(3-chloro-4-methoxy-anilino)-7-(3,4-dimethylphenyl)-5H-pyrrolo[2,3-d]pyrimidin-6-one (**39**)

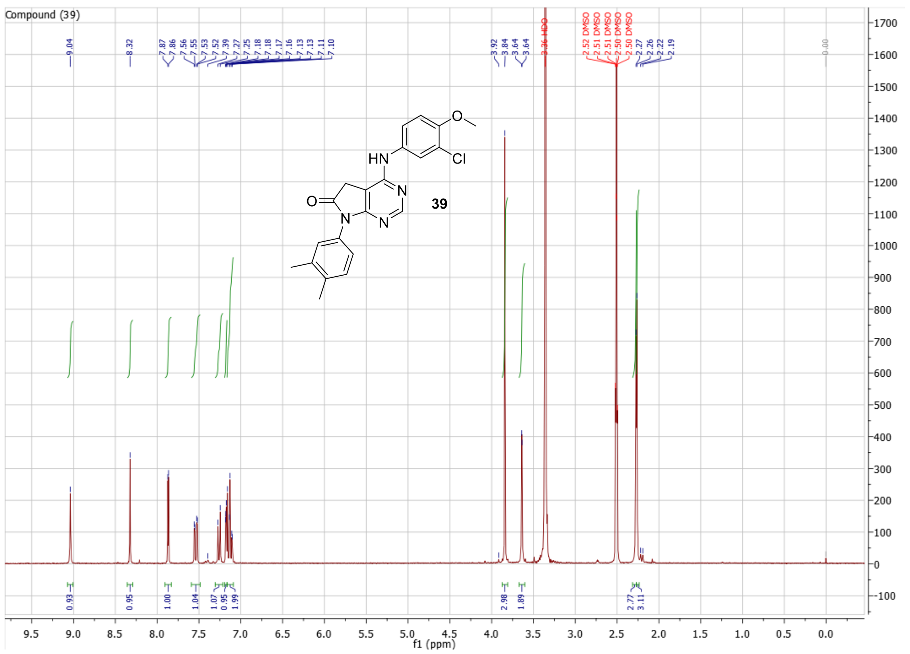

^{1}H NMR (300 MHz, DMSO-d_6) δ 9.04 (s, 1H), 8.32 (s, 1H), 7.87 (d, *J* = 2.6 Hz, 1H), 7.54 (dd, *J* = 9.0, 2.6 Hz, 1H), 7.26 (d, *J* = 8.1 Hz, 1H), 7.18 (d, *J* = 2.4 Hz, 1H), 7.13 (dd, *J* = 8.6, 6.8 Hz, 2H), 3.84 (s, 3H), 3.67 – 3.60 (m, 2H), 2.27 (s, 3H), 2.26 (s, 3H).

N-(3-((7-(3,4-dimethylphenyl)-7H-pyrrolo[2,3-d]pyrimidin-4-yl)amino)phenyl)acetamide (**43**)

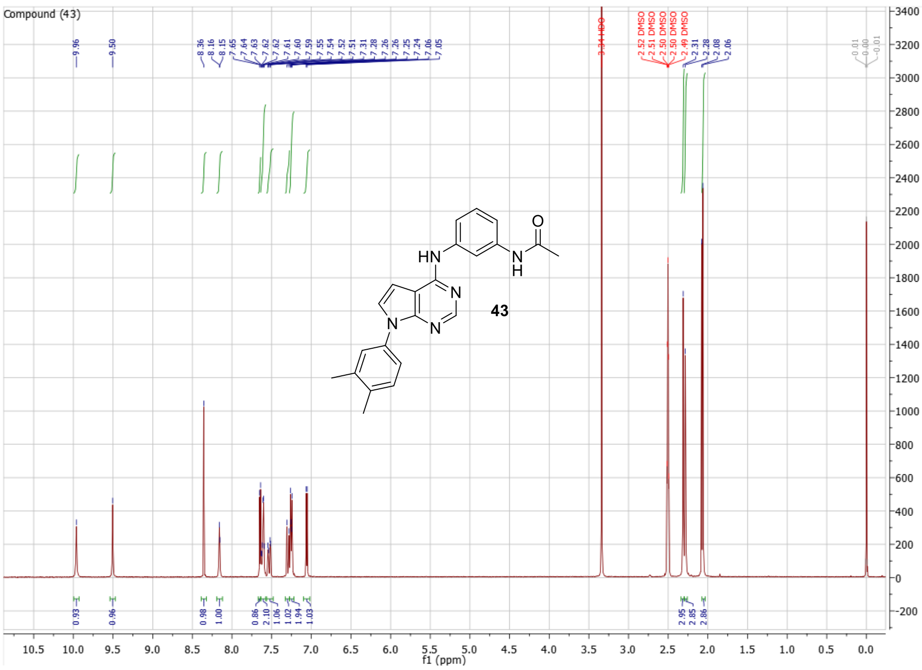

^{1}H NMR (300 MHz, DMSO-d_6) δ 9.96 (s, 1H), 9.50 (s, 1H), 8.36 (s, 1H), 8.16 (d, *J* = 2.0 Hz, 1H), 7.65 (d, *J* = 3.7 Hz, 1H), 7.63-7.58 (m, 2H), 7.53 (dd, *J* = 8.1, 2.3 Hz, 1H), 7.29 (d, *J* = 8.2 Hz, 1H), 7.28 – 7.22 (m, 2H), 7.06 (d, *J* = 3.7 Hz, 1H), 2.31 (s, 3H), 2.28 (s, 3H), 2.06 (s, 3H).

7-(3,4-dimethylphenyl)-N-(6-methoxypyridin-3-yl)-7H-pyrrolo[2,3-d]pyrimidin-4-amine (**45**)

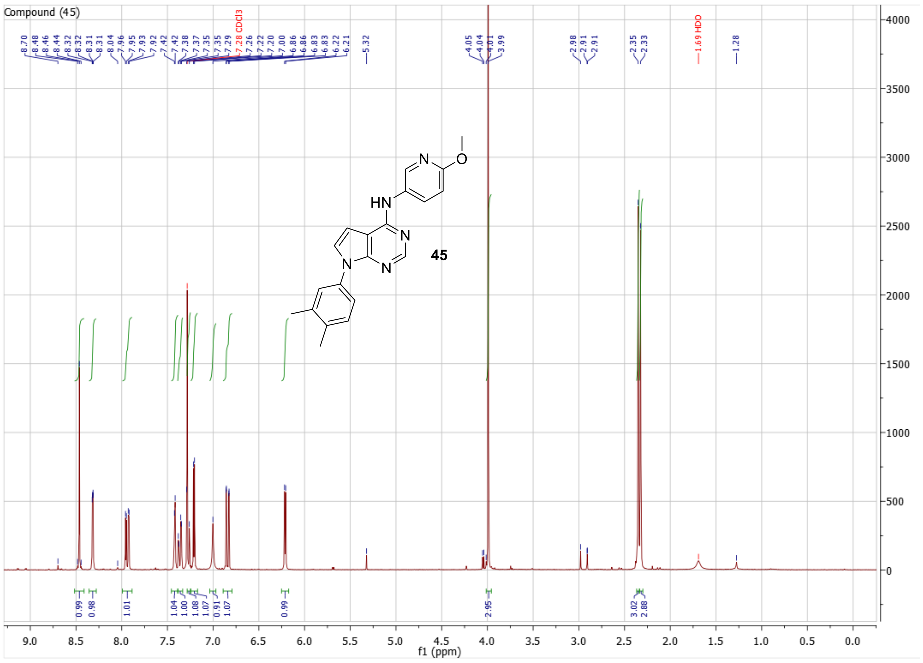

^{1}H NMR (300 MHz, Chloroform-*d*) δ 8.46 (s, 1H), 8.32 (dd, *J* = 2.8, 0.7 Hz, 1H), 7.94 (dd, *J* = 8.8, 2.8 Hz, 1H), 7.42 (d, *J* = 2.2 Hz, 1H), 7.36 (dd, *J* = 8.0, 2.3 Hz, 1H), 7.26 (s, 1H), 7.21 (d, *J* = 3.6 Hz, 1H), 7.00 (s, 1H), 6.84 (dd, *J* = 8.8, 0.7 Hz, 1H), 6.21 (d, *J* = 3.7 Hz, 1H), 3.99 (s, 3H), 2.35 (s, 3H), 2.33 (s, 3H).

Commercially available final compounds

The following compounds were purchased from the supplier indicated and used without further purification.

Compound	Supplier	Supplier reference
**8**	BioAscent	BCC0079802
**9**	Enamine	Z228679384
**10**	BioAscent	BCC0099025
**11**	BioAscent	BCC0100181
**12**	BioAscent	BCC0004551
**13**	Enamine	Z54200819
**14**	BioAscent	BCC0003114
**15**	BioAscent	BCC0001953
**16**	ChemDiv	C499-0447
**17**	Molport	K405-3569
**18**	Molport	STL336331
**19**	Molport	STL336152
**20**	Enamine	Z1041113980
**21**	Molport	STK944279
**22**	Chemtellect Inc	CS-74-038259
**23**	Enamine	Z1041114034
**24**	STK859322	STK859322
**25**	Molport	STL336454
**26**	Enamine	Z1041113988
**30**	BioAscent	BCC0045193
**32**	Molport	K405-3091

Table S1. Analogues of **10** for which PI5P4Kα and γ pIC_{50}s were determined. Another 52 compounds from the BioAscent collection with substructure similarity to **10** were tested at single point only and found to show <30% inhibition at 10 µM.

PI5P4Kα pIC_{50}	PI5P4Kγ+ pIC_{50}	SMILES	Supplier Reference	Supplier
5.0	5.0	Fc1ccc2n(cnc2c1)C1CCCN(C1)C(=O)c1cc(n[nH]1)-c1ccco1	BCC0098020	BioAscent
4.8	< 4.4	Cc1cc2nc(cc(n2n1)C(F)(F)F)C1CCCN(C1)C(=O)c1cc(on1)-c1ccco1	BCC0100858	BioAscent
< 4.4	< 4.4	O=C(NC1CCCC1)c1cc([nH]n1)-c1ccco1	BCC0014827	BioAscent
< 4.4	< 4.4	Cc1cc(no1)NC(=O)c1cc([nH]n1)-c1ccco1	BCC0014803	BioAscent
< 4.3	< 4.3	Cc1nnc(s1)C1CCCN(C1)C(=O)c1cc(n[nH]1)-c1ccco1	F5493-0522	Life chemicals
< 4.3	< 4.4	Clc1cnccc1OC1CCCN(C1)C(=O)c1cc(n[nH]1)-c1ccco1	F6480-1186	Life chemicals
< 4.3	< 4.3	O=C(N1CCCC(C1)Cc1nc(no1)C1CC1)c1cc(n[nH]1)-c1ccco1	F6497-0421	Life chemicals
< 4.3	< 4.3	Cn1ccc(n1)C1CCCN(C1)C(=O)c1cc(n[nH]1)-c1ccco1	F6554-0634	Life chemicals

Table S2. Analogues of **11** for which PI5P4Kα and γ pIC_{50}s were determined. Another 30 compounds from the BioAscent collection with substructure similarity to **11** were tested at single point only and found to show <40% inhibition at 10 µM.

PI5P4Kα pIC_{50}	PI5P4Kγ+ pIC_{50}	SMILES	Supplier Reference	Supplier
4.8	5.0	Fc1ccccc1CNc1ncc(c(n1)-c1ccco1)-c1cnccn1	BCC0099334	BioAscent
< 4.4	4.7	COc1ccc(cc1)OCCCNc1ncc(c(n1)-c1ccco1)-c1cnccn1	BCC0100112	BioAscent
5.4	5.5	Cc1noc(c1CCNc1ncc(c(n1)-c1ccco1)-c1cnccn1)C	BCC0100189	BioAscent
< 4.4	< 4.4	COc1cccc(c1)CNc1ncc(c(n1)-c1ccco1)-c1cc(no1)C	BCC0099140	BioAscent
5.2	4.9	Fc1ccc(cc1)CCNc1ncc(c(n1)-c1ccco1)-c1cnccn1	BCC0100757	BioAscent
4.9	4.4	Cc1cc(on1)-c1cnc(nc1-c1ccco1)NCc1cccc(c1)F	BCC0099144	BioAscent
< 4.8	5.8	Clc1ccccc1CNc1ncc(c(n1)-c1ccco1)-c1cnccn1	BCC0099207	BioAscent
4.6	< 4.4	CN(Cc1ccccc1)c1ncc(c(n1)-c1ccco1)-c1cc(no1)C	BCC0099237	BioAscent
< 4.4	< 4.7	Clc1ccccc1CCNc1ncc(c(n1)-c1ccco1)-c1cnccn1	BCC0099337	BioAscent
< 4.6	NA	CN1CCC(CC1)Nc1ncc(c(n1)-c1scnc1C)-c1ccc(cc1)F	Amb20119498	Ambinter
< 4.6	NA	Cn1ccnc1-c1nc(ncc1-c1ccc(cc1)F)NC1CCCC1	Amb20118393	Ambinter
< 4.6	NA	Cn1ccnc1-c1nc(ncc1-c1cccc(c1)F)NC1CCN(CC1)Cc1ccccc1	Amb13895326	Ambinter

Table S3. Additional commercial analogues of **8** for which PI5P4Kα and γ pIC_{50}s were determined.

PI5P4Kα pIC_{50}	PI5P4Kγ+ pIC_{50}	SMILES	Supplier Reference	Supplier
< 4.5	4.6	Clc1ccc(cc1)Nc1ncnc2n(ncc12)Cc1ccccc1	BCC0079216	BioAscent
< 4.3		Clc1ccc(cc1)Cn1ncc2c(ncnc12)NCCN1CCOCC1	BCC0084467	BioAscent
4.2	< 4.1	Cc1ccc(c(c1)C)-n1ncc2c(ncnc12)NCc1ccco1	BCC0088554	BioAscent
< 4.6		Cc1ccc(cc1)Nc1ncnc2n(ncc12)C	BCC0093607	BioAscent
4.8	4.7	C1COc2cc(ccc2O1)Nc1ncnc2n(ncc12)-c1ccccc1	BCC0091042	BioAscent
< 4.4	< 4.4	COc1cccc(c1)Nc1ncnc2n(ncc12)C	BCC0087286	BioAscent
4.6	5.1	CC(C)Nc1ncnc2n(ncc12)-c1ccc(cc1)Cl	BCC0088579	BioAscent
< 4.4	< 4.4	COc1ccc(cc1)Nc1ncnc2n(ncc12)C	BCC0003196	BioAscent
< 4.1	< 4.1	Cc1ccc(c(c1)C)-n1ncc2c(ncnc12)N1CCCC1	BCC0060184	BioAscent
< 4.3	< 4.3	Cn1ncc2c(ncnc12)Nc1ccc(c(c1)F)F	Z204264234	Enamine
< 4.4	4.6	CC(=O)Nc1ccc(cc1Cl)Nc1ncnc2n(ncc12)C	Z229452868	Enamine
4.5	< 4.3	Cc1ccc(cc1C)Nc1ncnc2n(ncc12)C	Z223814982	Enamine
< 4.3	< 4.3	Cc1ccc(cc1C)Nc1ncnc2n(ncc12)-c1cc(ccc1C)Cl	Z1041113974	Enamine
5.6	< 4.3	Clc1cccc(c1)Nc1ncnc2n(ncc12)-c1ccccc1	Z961104450	Enamine
< 4.3	< 4.3	Cn1ncc2c(ncnc12)Nc1cccc(c1)CN1CCSCC1	Z1127123360	Enamine
4.4	< 4.3	Cn1ncc2c(ncnc12)Nc1ccc(c(c1)F)OC(F)F	Z2241126335	Enamine
< 4.4	4.9	Cn1ncc2c(ncnc12)Nc1cc(c(c(c1)F)N1CCCC1)F	Z2241124305	Enamine
< 4.3	< 4.3	Cn1ncc2c(ncnc12)Nc1cccc(c1)Cn1cncn1	Z2241113362	Enamine
< 4.3	< 4.3	CC(=O)N1CCc2cc(ccc21)Nc1ncnc2n(ncc12)C	Z2241092261	Enamine
4.9	5.5	Cc1cc(ccc1N1CCCC1=O)Nc1ncnc2n(ncc12)C	Z229387906	Enamine
5.1	7	Cn1ncc2c(ncnc12)Nc1ccc(cc1)N1CCCC1=O	Z223819888	Enamine

4.8	6.4	COc1cc(ccc1N1CCCC1=O)Nc1ncnc2n(ncc12)C	Z229248342	Enamine
< 4.3	< 4.3	Cc1ccc(cc1C)-n1ncc2c(ncnc12)Nc1cccc(c1)Cl	K405-3091	Molport
< 4.3	< 4.3	COc1ccc(cc1)-n1ncc2c(ncnc12)Nc1ccc(c(c1)Cl)Cl	K402-0238	Molport
< 4.3	< 4.3	Fc1ccc(cc1Cl)Nc1ncnc2n(ncc12)-c1ccc(cc1)Cl	K402-0256	Molport
< 4.3	< 4.3	CC(=O)Nc1ccc(cc1)Nc1ncnc2n(ncc12)-c1ccccc1	STK895676	Molport
5.8	< 4.3	COc1cccc(c1)Nc1ncnc2n(ncc12)-c1ccc(cc1)C	STK846763	Molport
< 4.3	< 4.3	CN(C)c1nc(c2cnn(c2n1)-c1ccccc1)Nc1cccc(c1)Cl	STK890531	Molport
< 4.3	< 4.3	Clc1cccc(c1)Nc1nc(nc2n(ncc12)-c1ccccc1)N1CCOCC1	STK890534	Molport
6.0	< 4.3	Cc1ccc(cc1-n1ncc2c(ncnc12)Nc1cccc(c1)Cl)Cl	STK944236	Molport
< 4.5	< 4.3	Cc1ccc(cc1Cl)Nc1ncnc2n(ncc12)-c1ccccc1	STK889794	Molport
< 5.8	< 4.3	COc1ccc(cc1Cl)Nc1ncnc2n(ncc12)-c1ccc(cc1)F	STK859117	Molport
< 4.3	< 4.3	N(c1ccccc1)c1ncnc2n(ncc12)-c1ccccc1	STK731132	Molport
< 4.3	< 4.3	Cc1ccc(cc1Cl)-n1ncc2c(ncnc12)Nc1cccc(c1)Cl	STK944294	Molport
< 4.3	< 4.3	CNc1nc(c2cnn(c2n1)-c1ccccc1)Nc1cccc(c1)Cl	STK890512	Molport
< 4.3	< 4.3	COc1ccc(cc1Nc1ncnc2n(ncc12)-c1ccc(c(c1)Cl)C)Cl	STK944287	Molport
6.1	< 4.3	COc1ccc(cc1)-n1ncc2c(ncnc12)Nc1cccc(c1)Cl	STK889900	Molport
< 4.3	< 4.5	Cc1cccc(c1)-n1ncc2c(ncnc12)NCC1CCCO1	BCC0088601	BioAscent
< 4.5	4.7	Cn1ncc2c(ncnc12)Nc1ccc(cc1)-c1nccs1	BCC0010467	BioAscent
5.6	< 4.5	Cc1nc(cs1)-c1ccc(o1)CNc1ncnc2n(ncc12)C	BCC0011074	BioAscent
< 4.3	< 4.5	COc1ccc(cc1)C(=O)C1CCN(CC1)c1ncnc2n(ncc12)C	BCC0017566	BioAscent
5.1	NA	Clc1ccc(cc1)-n1ncc2c(ncnc12)NCc1cccnc1	K402-0947	ChemDiv
< 4.6	NA	Cn1ncc2c(ncnc12)NCCc1ccc(cc1)Cl	K405-2657	ChemDiv

Table S4. Potency data for **7** against PI5P4K isoforms using ADP-Glo^{TM} assays

**Kinase**	**pIC$_{50}$**
PI5P4Kα	**<4.3**
PI5P4Kβ	**<4.3**
PI5P4Kγ+	**<4.3**

Table S5. Crystallographic data and structure refinement for **7** (CSD: 2237386).

Crystal Habitus	Colourless block
Device Type	Bruker D8-QUEST PHOTON-100
Empirical formula	C17 H18 F3 N6 O2 +, Cl -
Formula weight	430.82
Temperature/K	180 (2)
Crystal system	Monoclinic
Space group	P 21/n
a/Å	6.6397(2)
b/Å	10.0511(3)
c/Å	28.1099(9
α/°	90
β/°	93.840(2)
γ/°	90
Volume/Å^3	1871.74(10)
Z	4
ρ_{calc}g/cm^3	1.529
μ/mm^{-1}	2.334
F(000)	888
Crystal size/mm^3	0.10 × 0.06 × 0.06
Absorption correction	Multi-scan
Radiation	CuK\α (λ = 1.5418)
2Θ range for data collection/°	3.15 to 66.83°
Completeness to theta	0.998
Index ranges	-7 ≤ h ≤ 7, -811≤ k ≤ 11, -33 ≤ l ≤ 30

Reflections collected	17933
Data/restraints/parameters	3304/0/282
Goodness-of-fit on F^2	1.060
Final R indexes [I>=2σ (I)]	R1 = 0.0348, wR2 = 0.0847
Final R indexes [all data]	R1 = 0.0422, wR2 = 0.0894

Table S6. Crystallographic bond lengths data for compound **7.**

Atom	Atom	Length/A	Atom	Atom	Length/A
F1	C7	1.327(2)	C2	C3	1.390(2)
F2	C7	1.349(2)	C2	H2A	0.95
F3	C7	1.332(2)	C3	C4	1.389(3)
O1	C8	1.233(2)	C3	C7	1.493(3)
O2	C13	1.233(2)	C4	C5	1.386(3)
N1	C8	1.349(2)	C4	H4A	0.95
N1	C1	1.414(2)	C5	C6	1.381(3)
N1	H1	0.84(2)	C5	H5A	0.95
N2	C10	1.352(2)	C6	H6A	0.95
N2	C11	1.357(2)	C8	C9	1.488(2)
N2	H2	0.83(3)	C9	C10	1.358(3)
N3	C11	1.312(2)	C9	C12	1.438(2)
N3	C12	1.355(2)	C10	H10A	0.95
N4	C12	1.319(2)	C14	C15	1.548(2)
N4	H41	0.85(3)	C14	H14A	0.99
N4	H42	0.89(3)	C14	H14B	0.99
N5	C11	1.358(2)	C15	C17	1.515(3)
N5	C13	1.410(2)	C15	C16	1.520(3)
N5	C14	1.468(2)	C16	H16A	0.98
N6	C13	1.323(2)	C16	H16B	0.98
N6	C15	1.468(2)	C16	H16C	0.98
N6	H6	0.86(2)	C17	H17A	0.98
C1	C2	1.387(2)	C17	H17B	0.98
C1	C6	1.393(3)	C17	H17C	0.98

Table S7. Crystallographic bond angles data for compound **7.**

Atom	Atom	Atom	Angle/°	Atom	Atom	Atom	Angle/°
C8	N1	C1	126.80(16)	O1	C8	C9	121.48(16)
C8	N1	H1	118.6(15)	N1	C8	C9	114.22(15)
C1	N1	H1	113.4(16)	C10	C9	C12	116.74(16)
C10	N2	C11	119.56(17)	C10	C9	C8	121.59(16)
C10	N2	H2	123.2(16)	C12	C9	C8	121.61(16)
C11	N2	H2	117.3(16)	N2	C10	C9	120.73(16)
C11	N3	C12	117.82(15)	N2	C10	H10A	119.6
C12	N4	H41	119.3(16)	C9	C10	H10A	119.6
C12	N4	H42	121.9(15)	N3	C11	N2	123.71(16)
H41	N4	H42	118(2)	N3	C11	N5	118.37(16)
C11	N5	C13	126.14(15)	N2	C11	N5	117.92(16)
C11	N5	C14	121.87(15)	N4	C12	N3	116.63(16)
C13	N5	C14	111.99(14)	N4	C12	C9	122.17(16)
C13	N6	C15	115.72(16)	N3	C12	C9	121.16(16)
C13	N6	H6	123.8(15)	O2	C13	N6	128.67(18)
C15	N6	H6	120.3(15)	O2	C13	N5	124.50(16)
C2	C1	C6	119.96(16)	N6	C13	N5	106.84(16)
C2	C1	N1	122.66(16)	N5	C14	C15	103.44(15)
C6	C1	N1	117.29(16)	N5	C14	H14A	111.1
C1	C2	C3	118.82(16)	C15	C14	H14A	111.1

C1	C2	H2A	120.6	N5	C14	H14B	111.1
C3	C2	H2A	120.6	C15	C14	H14B	111.1
C4	C3	C2	121.86(17)	H14A	C14	H14B	109
C4	C3	C7	119.34(16)	N6	C15	C17	109.53(17)
C2	C3	C7	118.77(16)	N6	C15	C16	109.88(16)
C5	C4	C3	118.30(17)	C17	C15	C16	111.47(18)
C5	C4	H4A	120.8	N6	C15	C14	102.01(14)
C3	C4	H4A	120.8	C17	C15	C14	112.37(17)
C6	C5	C4	120.84(18)	C16	C15	C14	111.17(17)
C6	C5	H5A	119.6	C15	C16	H16A	109.5
C4	C5	H5A	119.6	C15	C16	H16B	109.5
C5	C6	C1	120.21(17)	H16A	C16	H16B	109.5
C5	C6	H6A	119.9	C15	C16	H16C	109.5
C1	C6	H6A	119.9	H16A	C16	H16C	109.5
F1	C7	F3	107.62(17)	H16B	C16	H16C	109.5
F1	C7	F2	104.87(17)	C15	C17	H17A	109.5
F3	C7	F2	105.11(15)	C15	C17	H17B	109.5
F1	C7	C3	113.29(16)	H17A	C17	H17B	109.5
F3	C7	C3	113.17(16)	C15	C17	H17C	109.5
F2	C7	C3	112.10(17)	H17A	C17	H17C	109.5
O1	C8	N1	124.30(16)	H17B	C17	H17C	109.5

Table S8. Lipid kinase selectivity screening for **7** at 10 μM against a kinase panel of 22 lipid kinase targets generated using KINOMEscan™ technology[a] at DiscoverX.

**Kinase**	**% activity remaining**
PIK3C2B	**93**
PIK3C2G	**100**
PIK3CA	**100**
PIK3CA(C420R)	**100**
PIK3CA(E542K)	**100**
PIK3CA(E545A)	**100**
PIK3CA(E545K)	**100**
PIK3CA(H1047L)	**100**
PIK3CA(H1047Y)	**95**
PIK3CA(I800L)	**100**
PIK3CA(M1043I)	**100**
PIK3CA(Q546K)	**100**
PIK3CB	**100**
PIK3CD	**100**
PIK3CG	**100**
PIK4CB	**98**
PIKFYVE	**100**
PIP5K1A	**99**
PIP5K1C	**100**
PIP5K2B (PI5P4KB, PI5P4Kβ)	**100**
PIP5K2C (PI5P4KC)	**100**
VPS34	**100**

[a] Data were generated at Eurofins Discovery using DiscoverX KINOMEscan™ technology. Streptavidin-coated magnetic beads were treated with biotinylated small molecule ligands for 30 minutes at room temperature to generate affinity resins for kinase assays. The liganded beads were blocked with excess biotin and washed with blocking buffer (SeaBlock (Pierce), 1% BSA, 0.05% Tween 20, 1 mM DTT) to remove unbound ligand and to reduce non-specific binding. Binding reactions were assembled by combining kinases, liganded affinity beads, and test compounds in 1x binding buffer (20% SeaBlock, 0.17x PBS, 0.05% Tween 20, 6 mM DTT). Test compounds were prepared as 111X stocks in 100% DMSO. Kds were determined using an 11-point 3-fold compound dilution series with three DMSO control points. All compounds for Kd measurements are distributed by acoustic transfer (non-contact dispensing) in 100% DMSO. The compounds were then diluted directly into the assays such that the final concentration of DMSO was 0.9%. All reactions performed in polypropylene 384-well plate. Each was a final volume of 0.02 ml. The assay plates were incubated at room temperature with shaking for

1 hour and the affinity beads were washed with wash buffer (1x PBS, 0.05% Tween 20). The beads were then re-suspended in elution buffer (1x PBS, 0.05% Tween 20, 0.5 µM nonbiotinylated affinity ligand) and incubated at room temperature with shaking for 30 minutes. The kinase concentration in the eluates was measured by qPCR.

Table S9. Lipid kinase selectivity screening for **7** at 10 µM against a kinase panel of 15 protein kinase targets using ADP-Glo™ assay at the MRC PPU International Centre for Kinase Profiling, University of Dundee.

Kinase	% activity remaining	s.d.
PI3K g	**79**	8
PI3Ka E524K + p85	**93**	4
PIK4CB	**94**	0
DGK b	**97**	8
SPHK1	**98**	12
CHK b	**99**	1
SPHK2	**99**	5
DGK g	**100**	7
PI4K2a	**100**	1
CHK a	**101**	2
DGK z	**101**	9
PIP5K2A (PI5P4Kα)	**104**	4
PI3K a	**106**	7
PI3Ka E545K + p85	**107**	0
PI3K b	**109**	3

Table S10. lipid kinase selectivity screen at 1 µM for compound **36**. Conducted by MRC PPU International Centre for Kinase Profiling, using a 1 µM concentration of **36** against a panel of 16 lipid kinases.

protein	residual activity (%)	s.d.
PI3K beta	83.1	0.0
PI3K gamma	100.3	1.9
PI3K alpha	83.8	9.2
PI3Ka E524K + p85	101.5	2.3
PI3Ka E545K + p85	97.6	5.4
PI4K2a	95.7	4.3
PIK4Cb	100.6	5.1
CHK beta	93.4	5.2
CHK alpha	87.8	2.0
PIP5K2a	15.6	1.1
SPHK2	81.7	4.0
DGK beta	100.4	4.5
DGK gamma	105.5	1.2
DGK zeta	100.1	4.4
SPHK1	92.9	0.1

Table S11. premier kinase screen at 1 µM for compound **36**. Conducted by MRC PPU International Centre for Kinase Profiling, using a 1 µM concentration of **36** against a panel of 140 enzymes.

MRC PPU Gene Symbol	Entrez Gene Symbol	Residual activity (%)
ERK1	MAPK3	62
JAK3	JAK3	63
DYRK3	DYRK3	68
TTBK1	TTBK1	68
PDGFRA	PDGFRA	70
MLK1	MAP3K9	70
TTBK2	TTBK2	70

PINK	PINK1	73
PIM1	PIM1	75
IKKb	IKBKB	75
HIPK3	HIPK3	76
MARK4	MARK4	76
EIF2AK3	EIF2AK3	76
RSK2	RPS6KA3	77
PAK4	PAK4	78
EPH-B3	EPHB3	79
MAP4K5	MAP4K5	80
HER4	ERBB4	80
PKA	PRKAC	80
Aurora A	AURKA	81
MKK2	MAP2K2	82
CHK1	CHEK1	82
MLK3	MAP3K11	83
EPH-A2	EPHA2	83
TrkA	NTRK1	85
EF2K	EEF2K	85
JNK1	MAPK8	85
MST2	STK3	86
NEK2a	NEK2	86
PRK2	PKN2	86
PIM3	PIM3	87
TGFBR1	TGFBR1	87
OSR1	OXSR1	87
TTK	TTK	87
MNK1	MKNK1	87
TESK1	TESK1	88
S6K1	RPS6KB1	89
JNK3	MAPK10	89
IKKe	IKBKE	89
DYRK1A	DYRK1A	89
ABL	ABL1	89
PKBa	AKT1	90
PLK1	PLK1	90
HIPK2	HIPK2	90
MNK2	MKNK2	90
PRAK	MAPKAPK5	90
PAK6	PAK6	90
TAK1	MAP3K7	91
RIPK2	RIPK2	92
CK1γ2	CSNK1D	92
TBK1	TBK1	92
ULK2	ULK2	92
MELK	MELK	92
CK1δ	CSNK1G2	92
MAP4K3	MAP4K3	92
STK33	STK33	93
PKD1	PKD1	93
Src	SRC	93
AMPK (hum)	PRKAA1 + PRKAB2 + PRKAG1	94
PHK	PHK	94
CDK9-Cyclin T1	CDK9 + CCNT1	94
PAK5	PAK5	94
Lck	LCK	95
ERK5	MAPK7	95
FGF-R1	FGFR1	95
TLK1	TLK1	96
IRR	INSRR	96
ROCK 2	ROCK2	96
MPSK1	STK16	96

CHK2	CHEK2	96
CAMKKb	CAMKK2	96
PKCa	PRKCG	96
PIM2	PIM2	97
SGK1	SGK1	97
EPH-B2	EPHB2	97
CK2	CSNK2A1	97
BRSK1	BRSK1	97
SYK	SYK	97
TSSK1	TSSK1B	98
YES1	YES1	98
p38a MAPK	MAPK14	98
IRAK1	IRAK1	98
PAK2	PAK2	99
CAMK1	CAMK1	99
BTK	BTK	99
ZAP70	ZAP70	99
SmMLCK	MYLK	99
EPH-B4	EPHB4	99
SRPK1	SRPK1	99
MST4	STK26	99
VEG-FR	FLT1	99
CDK2-Cyclin A	CDK2 + CCNA2	100
p38g MAPK	MAPK13	101
LKB1	STK11	101
EPH-A4	EPHA4	101
MAPKAP-K3	MAPKAPK3	101
ULK1	ULK1	102
GSK3b	GSK3B	102
p38b MAPK	MAPK11	103
MSK1	RPS6KA5	103
PKBb	AKT2	103
NEK6	NEK6	104
CSK	CSK	104
HIPK1	HIPK1	104
MARK3	MARK3	104
TIE2	TEK	105
WNK1	WNK1	105
GCK	GCK	105
JNK2	MAPK9	106
ASK1	MAP3K5	107
PKCγ	PRKCZ	107
CLK2	CLK2	107
MINK1	MINK1	107
DYRK2	DYRK2	108
IR	INSR	108
SIK2	SIK2	109
RSK1	RPS6KA1	109
MAPKAP-K2	MAPKAPK2	109
ERK2	MAPK1	110
ERK8	MAPK15	110
Aurora B	AURKB	110
PKCz	PRKCA	111
BRSK2	BRSK2	111
TAO1	TAOK1	111
MKK1	MAP2K1	112
MARK1	MARK1	113
IRAK4	IRAK4	113
DDR2	DDR2	115
MKK6	MAP2K6	115
MEKK1	MAP3K1	115
DAPK1	DAPK1	118
IGF-1R	IGF1R	119
SIK3	SIK3	123

MST3	STK24	124
BRK	PTK6	126
EPH-B1	EPHB1	126
MARK2	MARK2	127
p38d MAPK	MAPK12	128
PDK1	PDPK1	133
NUAK1	NUAK1	138

Table S12. Data collection and refinement statistics for X-ray crystal structures of PI5P4Kα bound to **36.**

PDB ID	8C8C
Protein/Ligand	PI5P4Kα/**36**
Wavelength [Å]	0.9795
Space group	C 2 2 2_1
Cell dimensions	
a; b; c; [Å]	70.66; 84.94; 128.35
α; β; γ; [◦]	90.0; 90.0; 90.0
Resolution [Å]	2.10 (2.16-2.10) [a]
Unique reflections	19434 (368) [2]
Multiplicity	1.9 (1.9)[2]
Completeness [%]	84.7 (19.6) [2]
R_{sym} [%]	4.0(53.2)[2]
R_{meas} [%]	5.6 (75.3)[2]
Mean(I)/sd	10.3 (1.4)[2]
CC(1/2)	0.996 (0.766)
Number of reflections (free)	32790(1640)
R_{cryst} [%]	21.7
R_{free}[%]	28.4
Total number of atoms:	
Protein	2429
Water	130
Ligand	27
Deviation from ideal geometry:	
Bond lengths [Å]	0.012
Bond angles [◦]	1.79

[a]values in parenthesis refer to the highest resolution bin.

Table S13. Crystallographic data and structure refinement for compound **20** (CSD: 2237387)**.**

Crystal Habitus	Colourless block
Device Type	Bruker D8-QUEST PHOTON-100
Empirical formula	C19 H15 Cl2 N5 O
Formula weight	400.26
Temperature/K	180 (2)
Crystal system	Monoclinic
Space group	P 21/c
a/Å	11.3629(3)
b/Å	7.1919(2)
c/Å	22.2901(6)
α/°	90
β/°	101.2060(10)
γ/°	90
Volume/$Å^3$	1786.84(8)
Z	4
ρ_{calc}g/cm^3	1.488
μ/mm^{-1}	3.440

F(000)	824
Crystal size/mm^3	0.22 × 0.10 × 0.05
Absorption correction	Multi-scan
Radiation	CuK\α (λ = 1.5418)
2Θ range for data collection/°	3.97 to 70.32°
Completeness to theta	0.999
Index ranges	-13 ≤ h ≤ 13, -8 ≤ k ≤ 8, -27 ≤ l ≤ 27
Reflections collected	30283
Data/restraints/parameters	3416/0/250
Goodness-of-fit on F^2	1.061
Final R indexes [I>=2σ (I)]	R1 = 0.0306, wR2 = 0.0746
Final R indexes [all data]	R1 = 0.0360 wR2 = 0.0772

Table S14. Crystallographic bond lengths data for compound **20.**

Atom	Atom	Length/A	Atom	Atom	Length/A
Cl1	C13	1.7400(15)	C12	H12A	0.95
Cl2	C21	1.7405(16)	C13	C14	1.399(2)
O1	C14	1.3621(19)	C14	C15	1.386(2)
O1	C17	1.433(2)	C15	C16	1.393(2)
N1	C6	1.3415(19)	C15	H15A	0.95
N1	C2	1.349(2)	C16	H16A	0.95
C2	N3	1.325(2)	C17	H17A	0.98
C2	H2A	0.95	C17	H17B	0.98
N3	C4	1.3489(19)	C17	H17C	0.98
C4	N9	1.3562(19)	C18	C19	1.380(2)
C4	C5	1.397(2)	C18	C23	1.395(2)
C5	C7	1.416(2)	C19	C20	1.389(2)
C5	C6	1.421(2)	C19	H19A	0.95
C6	N10	1.3515(19)	C20	C21	1.382(2)
C7	N8	1.3213(19)	C20	H20A	0.95
C7	H7A	0.95	C21	C22	1.379(2)
N8	N9	1.3744(17)	C22	C23	1.395(2)
N9	C18	1.4329(18)	C22	H22A	0.95
N10	C11	1.4153(19)	C23	C24	1.502(2)
N10	H10	0.84(2)	C24	H24A	0.98
C11	C16	1.391(2)	C24	H24B	0.98
C11	C12	1.395(2)	C24	H24C	0.98
C12	C13	1.377(2)			

Table S15. Crystallographic bond angle data for compound **20.**

Atom	Atom	Atom	Angle/°	Atom	Atom	Atom	Angle/°
C14	O1	C17	116.87(13)	C14	C15	C16	121.65(15)
C6	N1	C2	117.96(13)	C14	C15	H15A	119.2
N3	C2	N1	129.67(14)	C16	C15	H15A	119.2
N3	C2	H2A	115.2	C11	C16	C15	120.19(15)
N1	C2	H2A	115.2	C11	C16	H16A	119.9
C2	N3	C4	110.83(13)	C15	C16	H16A	119.9
N3	C4	N9	125.83(13)	O1	C17	H17A	109.5
N3	C4	C5	126.92(14)	O1	C17	H17B	109.5
N9	C4	C5	107.21(12)	H17A	C17	H17B	109.5
C4	C5	C7	104.54(13)	O1	C17	H17C	109.5
C4	C5	C6	115.62(13)	H17A	C17	H17C	109.5
C7	C5	C6	139.73(14)	H17B	C17	H17C	109.5
N1	C6	N10	120.64(13)	C19	C18	C23	122.11(14)
N1	C6	C5	118.92(13)	C19	C18	N9	118.66(13)
N10	C6	C5	120.38(13)	C23	C18	N9	119.18(13)

N8	C7	C5	111.26(13)	C18	C19	C20	119.96(15)
N8	C7	H7A	124.4	C18	C19	H19A	120
C5	C7	H7A	124.4	C20	C19	H19A	120
C7	N8	N9	106.11(12)	C21	C20	C19	118.36(15)
C4	N9	N8	110.87(11)	C21	C20	H20A	120.8
C4	N9	C18	127.07(12)	C19	C20	H20A	120.8
N8	N9	C18	121.87(11)	C22	C21	C20	121.76(15)
C6	N10	C11	129.01(13)	C22	C21	Cl2	119.03(13)
C6	N10	H10	114.2(13)	C20	C21	Cl2	119.20(13)
C11	N10	H10	116.0(13)	C21	C22	C23	120.52(15)
C16	C11	C12	118.55(14)	C21	C22	H22A	119.7
C16	C11	N10	124.76(14)	C23	C22	H22A	119.7
C12	C11	N10	116.70(13)	C18	C23	C22	117.28(15)
C13	C12	C11	120.62(14)	C18	C23	C24	121.60(14)
C13	C12	H12A	119.7	C22	C23	C24	121.12(15)
C11	C12	H12A	119.7	C23	C24	H24A	109.5
C12	C13	C14	121.55(14)	C23	C24	H24B	109.5
C12	C13	Cl1	118.60(11)	H24A	C24	H24B	109.5
C14	C13	Cl1	119.83(12)	C23	C24	H24C	109.5
O1	C14	C15	125.38(14)	H24A	C24	H24C	109.5
O1	C14	C13	117.21(14)	H24B	C24	H24C	109.5
C15	C14	C13	117.41(14)				

ADMET experimental methods

MDR1-MDCK Permeability (bi-directional): was performed by Cyprotex Discovery. Briefly, compounds were administered at 10 µM (1% DMSO final) to the apical or basolateral side of a confluent monolayer of MDR1-MDCK cells, then incubated at 37 °C for 60 minutes before appearance on the opposite side of the monolayer was determined LC-MS/MS. The efflux ratio (ER) is calculated from the ratio of B-A to A-B permeabilities.

Turbidimetric aqueous solubility: analysis was performed by Cyprotex Discovery. Compound in DMSO at 10 mM was serially diluted to solutions of 0.1, 0.3, 1 and 3 mM in DMSO, then further diluted 1 in 100 in buffer (0.01 M PBS, pH 7.4, 1% DMSO final). 7 replicate wells of each dilution were equilibrated for 2 hours at 37 °C before absorbance is measured at 620 nm. The solubility is estimated from the concentration of test compound that produces an increase in absorbance above vehicle control (1% DMSO in buffer).

Mouse Microsomal stability: analysis was performed by Cyprotex Discovery. Briefly, test compounds in DMSO were incubated at a concentration of 1 µM (0.25% DMSO final) with mouse hepatic microsomes (0.5 mg protein/mL) in the presence of NADPH (1 mM) at 37 °C. Aliquots were taken at time intervals (0, 5, 15, 30 and 45 min) and stopped by transferring into acetonitrile, then analysed using generic LC-MS/MS conditions for compound remaining, allowing the determination of the half-life for the compound.

Consideration of dihedral angles for 32, 35 and 36

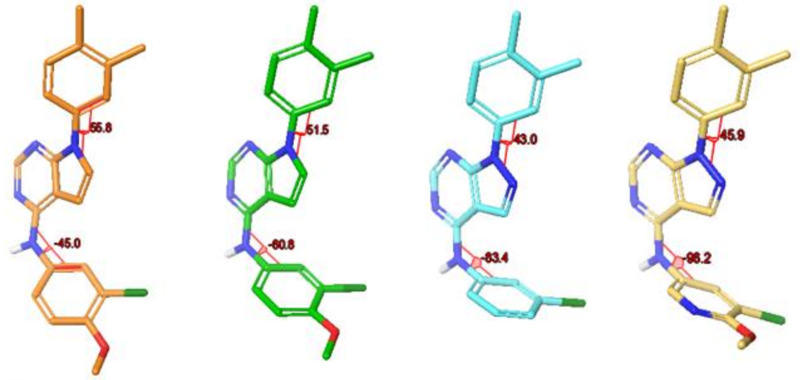

36 *8C8C crystal* | **36** *Ab initio* optimized | **32** *Ab initio* optimized | **35** *Ab initio* optimized

The ω angle has a different ground state for **36**, **32** and **35** in the 'bound' conformation (main manuscript figure 4A; ψ = -7.5). In the crystal structure this is -45 degrees. QM optimization of the crystal structure conformation of **36** changes this ω angle to -61 degrees (energy difference between X-tal state and optimized state 9.6 kcals). This optimized conformation of **36** still fits into the active site. However, the ω angles of **32** and **35** change to -83 and -96 degrees, respectively, on optimization. **32** and **35** do not fit into the crystal structure in that conformation as shown below:

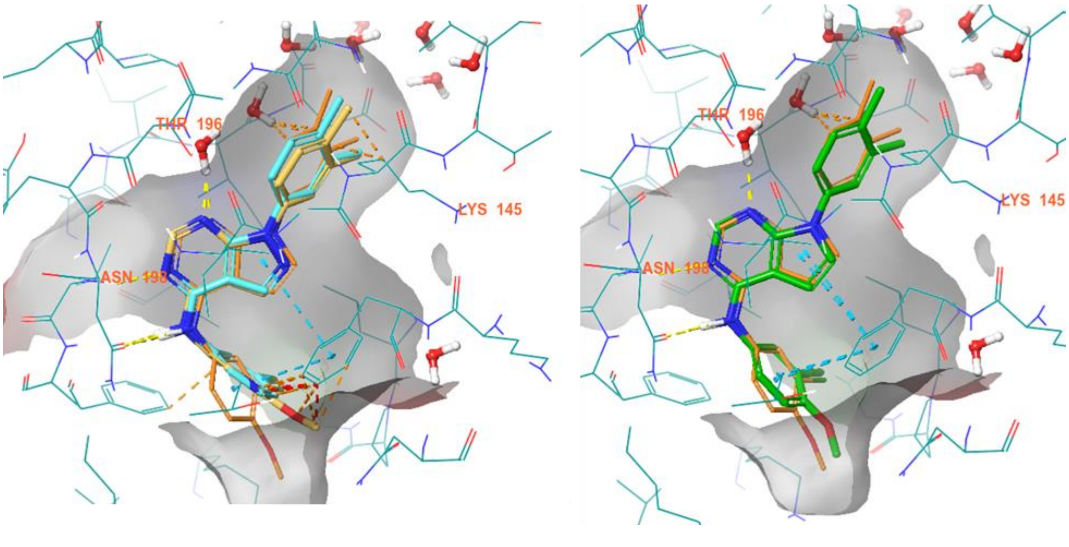

Left: **32** (cyan) and **35** (yellow) in their optimized conformation clash with Phe134 (indicated by orange and red dotted lines). Right: Ab initio optimized **36** does not show this clash. All ligands started from the same geometry and the same ab initio optimization was used.

References

(1) Abdou, N. S.; Serya, R. A. T.; Esmat, A.; Tolba, M. F.; Ismail, N. S. M.; Abouzid, K. A. M. Synthesis and in Vitro Antiproliferative Activity of Novel Pyrazolo[3,4-d]Pyrimidine Derivatives. *Medchemcomm* **2015**, *6* (8), 1518–1534.

(2) Das, J.; Moquin, R. v.; Pitt, S.; Zhang, R.; Shen, D. R.; McIntyre, K. W.; Gillooly, K.; Doweyko, A. M.; Sack, J. S.; Zhang, H.; Kiefer, S. E.; Kish, K.; McKinnon, M.; Barrish, J. C.; Dodd, J. H.; Schieven, G. L.; Leftheris, K. Pyrazolo-Pyrimidines: A Novel Heterocyclic Scaffold for Potent and Selective P38α Inhibitors. *Bioorg Med Chem Lett* **2008**, *18* (8), 2652–2657.

(3) Voss, M. D.; Czechtizky, W.; Li, Z.; Rudolph, C.; Petry, S.; Brummerhop, H.; Langer, T.; Schiffer, A.; Schaefer, H. L. Discovery and Pharmacological Characterization of a Novel Small Molecule Inhibitor of Phosphatidylinositol-5-Phosphate 4-Kinase, Type II, Beta. *Biochem Biophys Res Commun* **2014**, *449* (3), 327–331.

(4) Kampe, K-D,; Granzer, E.; Leineweber, D.; Rackur, G.; Boeger, H. G. 4-Amino-2-ureidopyrimidine-5-carboxamides, a process for thepreparation thereof, pharmaceuticals containing these compounds, and the use thereof. **2002**, CA2089954C.
